# Global disparities in dengue, climate, and extreme weather research: a bibliometric analysis of alignment with disease burden and climate-driven vector suitability

**DOI:** 10.1186/s41182-026-01110-6

**Published:** 2026-11-13

**Authors:** Chunyue Ai, Yi Teng, Guangju Mo, Wenyu Wang, Meshack Kipkogei Biwott, Jing Li, Qiyong Liu

**Affiliations:** 1 School of Public Health, Shandong Second Medical University, No. 7166, Baotong West Street, Weifang, 261053 China; 2 National Key Laboratory of Intelligent Tracking and Forecasting for Infectious Diseases, National Institute for Communicable Disease Control and Prevention, Chinese Center for Disease Control and Prevention & Chinese Academy of Preventive Medicine, No. 155 Changbai Road, Changping District, Beijing, 102206 China

**Keywords:** Dengue fever, Extreme weather, Climate change, Disease burden, Bibliometric analysis, Vector suitability

## Abstract

**Background:**

Dengue is a climate-sensitive vector-borne disease that imposes a substantial public health burden in tropical and subtropical regions. Temperature, rainfall, and extreme weather can affect mosquito ecology, human exposure, and the environmental suitability for transmission. This study characterized the global research landscape of dengue and extreme weather and examined whether country-level research output corresponded to dengue burden and climate-driven vector suitability.

**Methods:**

The Web of Science Core Collection was searched on 22 May 2026. Publication trends, collaboration networks, journals, authors, co-cited references, citation bursts, keyword patterns, and thematic evolution were examined using CiteSpace, VOSviewer, and the bibliometrix R package. Country-level publication output was linked to estimates from the Global Burden of Disease 2023 dataset, the socio-demographic index for 2021, and mean index–*p* values for 1990–2022. Associations between publication output and dengue burden were assessed using Spearman’s rank correlation among countries classified as dengue-endemic.

**Results:**

A total of 290 publications from 2008 to 2026 met the eligibility criteria. Annual publication output increased overall and remained comparatively high after 2020. The United States contributed the largest number of publications, followed by China, the United Kingdom, Australia, and Brazil. The literature connected tropical medicine and infectious disease epidemiology with environmental health, climate science, vector ecology, modeling, and public health. Major themes included climate change, temperature, rainfall, the El Niño–Southern Oscillation, extreme weather, Aedes vectors, transmission modeling, risk prediction, and early warning. Among 80 countries classified as dengue-endemic, publication output was positively correlated with the age-standardized incidence rate (ASIR) in 2023 (Spearman’s ρ = 0.32, *P* = 0.004, *n* = 80) and the disability-adjusted life-year (DALY) rate in 2023 (ρ = 0.36, *P* < 0.001, *n* = 80). In contrast, its association with the estimated annual percentage change (EAPC) in ASIR from 1990 to 2023 was not statistically significant (ρ = 0.20, *P* = 0.070, *n* = 80). Several dengue-endemic countries with comparatively high disease burden or long-term climate-driven vector suitability had no eligible Web of Science-indexed publications in the present dataset.

**Conclusions:**

Research on dengue and extreme weather has expanded, and publication output showed modest positive associations with contemporary dengue burden among dengue-endemic countries. However, research output was not significantly associated with the long-term trend in dengue incidence, and substantial geographic disparities remained. The separate assessment of burden, research output, and climate-driven vector suitability identified geographic areas that may warrant closer consideration when research capacity, surveillance support, and equitable international partnerships are developed.

**Supplementary Information:**

The online version contains supplementary material available at https://doi.org/10.1186/s41182-026-01110-6.

## Introduction

Dengue fever (DF), an acute mosquito-borne infectious disease caused by dengue virus (DENV), is primarily transmitted by *Aedes aegypti* and *Aedes albopictus*. It is one of the most rapidly expanding arboviral diseases worldwide and has become a major public health concern in tropical and subtropical regions. According to the World Health Organization (WHO), as of April 30, 2024, more than 7.6 million dengue cases had been reported globally, including approximately 3.4 million confirmed cases, over 16,000 severe cases, and more than 3,000 deaths. Active dengue transmission was reported in 90 countries and territories in 2024, indicating a sustained global threat [1]. Recent global estimates indicate that dengue continues to impose a substantial disease burden, while climate-driven suitability for transmission has expanded in several regions [2, 3].

The global expansion of dengue is shaped by multiple interacting factors, including urbanization, population mobility, environmental change, vector distribution, and public health capacity. Climate change has been recognized by WHO as an important driver of dengue transmission [1]. The Intergovernmental Panel on Climate Change (IPCC) reported with high confidence that dengue is closely associated with climatic variables at both global and regional scales, with temperature, rainfall, humidity, and extreme weather events playing important roles in dengue transmission [4]. Modeling studies further indicate that climate change may alter the geographical distribution and transmission suitability of Aedes-borne viruses, including dengue, Zika, and chikungunya [5, 6].

The impact of climate change on dengue transmission is reflected not only in long-term warming and altered precipitation patterns, but also in the increasing frequency, intensity, and spatial extent of extreme weather events. Typhoons, heavy rainfall, floods, droughts, and persistent high temperatures can modify mosquito breeding habitats, vector abundance, seasonal dynamics, and human exposure patterns, thereby reshaping dengue transmission risk. Beyond their direct effects on disease ecology, climate-related hazards may also increase pressure on public health systems through broader impacts on population health, social vulnerability, and emergency response capacity [4, 7].

Meteorological factors play a central role in dengue transmission. Higher temperatures can shorten the extrinsic incubation period of DENV, increase mosquito biting frequency, and expand environmentally suitable areas for Aedes vectors, thereby enhancing transmission potential [8, 9]. Mechanistic modeling studies have further shown that temperature influences mosquito development, survival, reproduction, biting rate, viral replication, and overall vectorial capacity, resulting in temperature-dependent suitability for dengue and other Aedes-borne viruses [10]. Rainfall and hydrological conditions may also affect mosquito breeding and dengue incidence. Several studies have reported nonlinear or delayed associations between rainfall, hydrological anomalies, and dengue risk [11, 12]. In addition, climate warming may disrupt rainfall distribution and increase the availability of Aedes breeding habitats, further increasing the likelihood of dengue outbreaks [13]. Large-scale climate oscillations, such as the El Niño–Southern Oscillation (ENSO), have also been associated with interannual and temporal fluctuations in dengue incidence across different regions [14, 15].

In recent years, research on dengue transmission has increasingly focused on meteorological factors, climate variability, climate change, and extreme weather events. Existing studies have examined the effects of temperature, rainfall, humidity, ENSO, floods, droughts, heatwaves, and hydrometeorological anomalies on dengue incidence, vector ecology, outbreak risk, and future spatial distribution. For example, a modeling study in Barbados quantified the nonlinear and delayed effects of climate indicators, including drought and extreme rainfall, on dengue risk [16]. In parallel, climate-informed prediction models and early warning systems have been developed to support dengue risk assessment, outbreak preparedness, and public health decision-making [17, 18].

Despite these advances, the literature on dengue fever and extreme weather remains dispersed across different disciplines, regions, and methodological frameworks [5, 19]. Relevant studies span tropical medicine, infectious disease epidemiology, environmental health, climate science, vector ecology, mathematical modeling, and public health. However, a comprehensive bibliometric overview of global research trends, collaboration patterns, knowledge structure, research hotspots, and thematic evolution in this interdisciplinary field remains limited.

Bibliometric methods can be used to examine publication growth, collaboration networks, co-citation structures, and changes in research themes [20–23]. Although such methods have been applied in public health, infectious disease research, and climate-related health studies, little attention has been paid to whether the geographic distribution of research on dengue and weather corresponds to disease burden or climatic suitability for transmission. The present study, therefore, combined science-mapping approaches with country-level epidemiological and environmental indicators to examine both the development of the literature and its geographic alignment with dengue-related public health needs.

This study aimed to characterize the global development and knowledge structure of research on dengue and extreme weather and to assess whether the geographic distribution of research activity corresponded to dengue burden and climate-driven vector suitability. Specifically, we examined publication trends, collaboration patterns, influential sources, co-citation structures, and thematic evolution; quantified associations between country-level publication output and dengue burden; and identified countries in which high disease burden and climatic suitability coincided with limited indexed research output.

## Methods

### Data source and search strategy

A bibliometric analysis was performed to characterize global research trends, collaboration patterns, knowledge structure, research hotspots, and future directions in studies on dengue fever and extreme weather. Publications were retrieved from the Web of Science Core Collection (WoSCC), a commonly used database for bibliometric research. To minimize the influence of subsequent database updates, all records were searched and exported on May 22, 2026. The publication period was set from 2008 to 2026.

The search strategy combined dengue-related terms with terms related to extreme weather and climate. Dengue-related terms included “dengue”, “dengue fever”, “dengue virus”, “DENV”, “*Aedes aegypti*”, and “*Aedes albopictus*”. Weather- and climate-related terms included “extreme weather”, “climate change”, “climate variability”, “temperature”, “rainfall”, “precipitation”, “flood”, “drought”, “heatwave”, “storm”, “cyclone”, “hurricane”, “typhoon”, “El Niño”, and “ENSO”. The search was conducted in topic fields, including title, abstract, author keywords, and Keywords Plus. The full search strategy and retrieval date are provided in Supplementary File Methods S1.

Only English-language articles and reviews were included. Other document types, including meeting abstracts, letters, editorials, corrections, book chapters, and proceedings papers, were excluded. Two reviewers independently screened the titles and abstracts against the eligibility criteria. Disagreements were resolved through discussion, with consultation of a third reviewer when consensus could not be reached.

### Literature screening and eligibility criteria

**Fig. 1:**
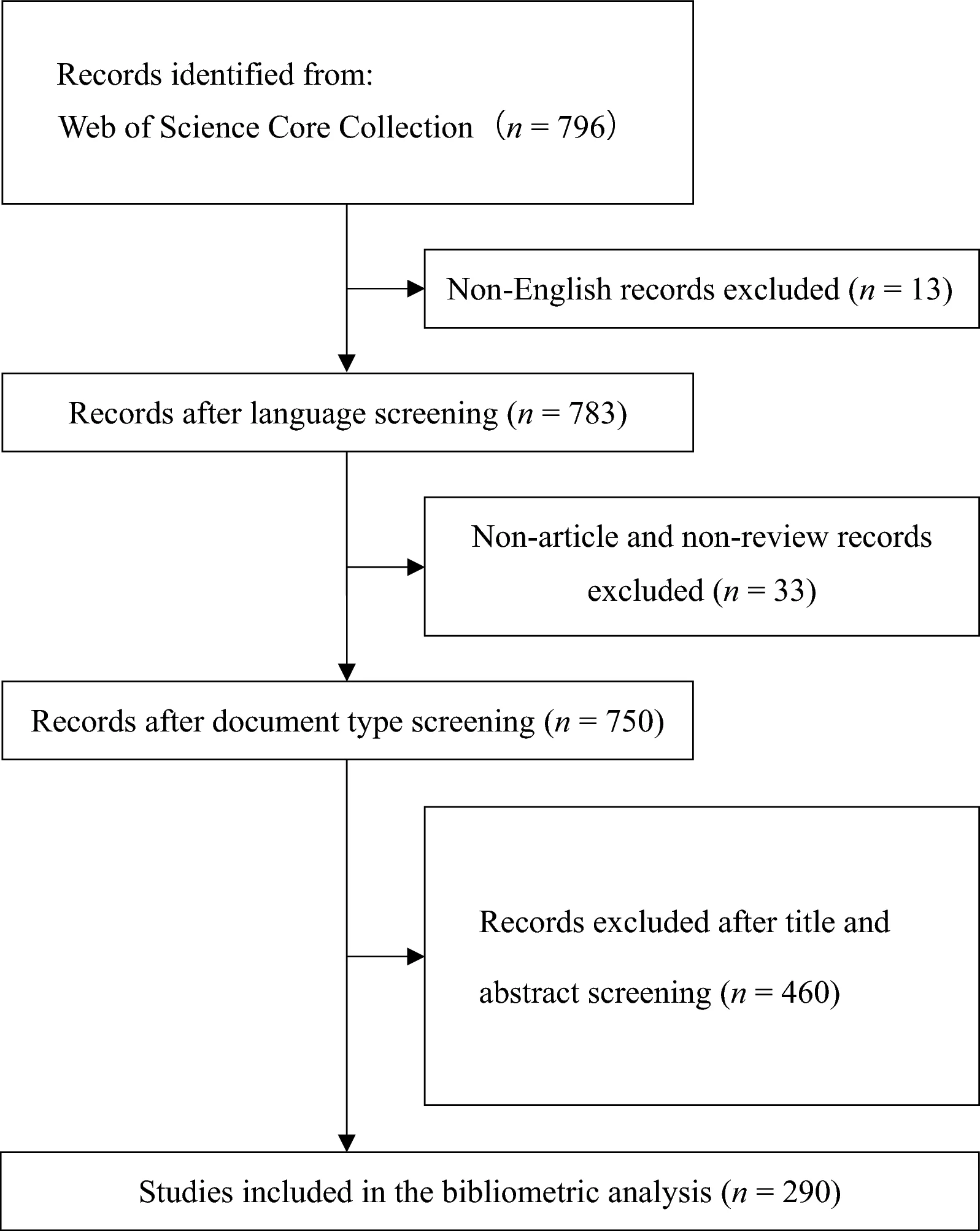
Flow diagram of the literature search and screening process

The literature screening process is presented in Fig. 1. A total of 796 records were initially identified from WoSCC. After excluding 13 non-English publications and 33 records that were not articles or reviews, the remaining records were screened by title and abstract. Of these, 460 publications were excluded, because they were not directly related to dengue fever, dengue virus, Aedes vectors, extreme weather, climate variability, climate change, or meteorological factors. Finally, 290 publications were included in the bibliometric analysis.

Studies were included if they substantively investigated the relationship between dengue or the target vectors (*Aedes aegypti *or *Aedes albopictus*) and climate change, climate variability, extreme weather events, weather conditions, or meteorological factors. Relevant outcomes included dengue incidence, transmission, outbreaks, disease burden, spatial or temporal risk, forecasting, and climate-related changes in vector distribution, abundance, survival, development, or population dynamics. The presence of dengue- and climate-related terms alone was not considered sufficient for inclusion.

Records were excluded if they met any of the following criteria: (1) not substantively related to dengue or the target Aedes vectors; (2) vector-only studies without a dengue-related outcome or context; (3) no relevant climate or meteorological exposure; (4) dengue mentioned only as background or as an example; (5) temperature or heat representing a technical, laboratory, or biomedical condition rather than a meteorological exposure; (6) vector-control or intervention studies without assessment of a climate–dengue association; (7) general climate-health, disaster, or other-disease studies without dengue-specific analysis; (8) clinical, diagnostic, immunological, pathogenesis, or therapeutic dengue studies; or (9) dengue epidemiology, transmission, or social-factor studies without climate-exposure analysis.

Two reviewers independently re-screened all records at the title and abstract level using the revised operational eligibility criteria. Disagreements were resolved through discussion and, when necessary, consultation with a third reviewer. The complete list of excluded records, including bibliographic information, primary exclusion reasons, and record-specific justifications, is provided in Supplementary Table S1.

### Data extraction and preprocessing

The full records and cited references of the included publications were exported from WoSCC in plain text format. The extracted information included publication year, title, authors, institutions, countries or regions, journal, abstract, author keywords, Keywords Plus, cited references, and citation information.

Before analysis, the dataset was checked and cleaned, and duplicate and irrelevant records were removed. When necessary, author names, institution names, country or region names, and keywords were standardized to reduce inconsistencies caused by spelling variants, abbreviations, and different naming formats. Similar keywords, such as “climate change” and “climatic change”, as well as abbreviated species names, such as “*Ae. aegypti*” and “*Aedes aegypti*”, were merged where appropriate. The cleaned dataset was then imported into Microsoft Excel, VOSviewer, CiteSpace, and the R package bibliometrix for bibliometric analysis and visualization.

### Bibliometric analysis and visualization

Bibliometric analyses were conducted using Microsoft Excel 2024, VOSviewer version 1.6.20, CiteSpace version 6.4.2, and the bibliometrix package in R version 4.5.2. Microsoft Excel was used to summarize annual publication output and describe temporal publication trends from 2008 to 2026.

VOSviewer was used to construct and visualize collaboration, co-citation, and keyword co-occurrence networks. Country and institutional collaboration networks were generated to examine global cooperation patterns. Journal and co-cited journal networks were used to identify major publication sources and knowledge sources. Author and co-cited author networks were used to identify productive authors, influential scholars, and collaboration patterns. Co-cited reference networks were constructed to explore the intellectual base of the field. Keyword co-occurrence networks were used to identify major research hotspots. For keyword co-occurrence analysis, the minimum number of occurrences of a keyword was set to 5. Of the 688 keywords identified, 28 met the threshold and were included in the final keyword co-occurrence network. In the VOSviewer maps, nodes represented analytical units, such as countries, institutions, journals, authors, references, or keywords. Node size indicated publication output, occurrence frequency, or co-citation frequency, depending on the analysis. Links between nodes represented collaboration, co-citation, or co-occurrence relationships, and thicker links indicated stronger associations.

CiteSpace was used to perform citation burst analysis and to generate the dual-map overlay of journals. In CiteSpace, the time slicing was set from 2008 to 2026, with 1 year per slice. Link strength within each time slice was normalized using the cosine similarity measure, with higher values indicating stronger relationships between nodes. For citation burst and co-citation analyses, cited references and cited journals were selected as node types. The g-index was used as the selection criterion, with k = 25. Citation bursts were detected using Kleinberg’s burst-detection algorithm. Burst strength is an algorithm-derived value that reflects the intensity of an abrupt increase in citations to a reference during a specific period; a higher value indicates a more pronounced citation surge. Citation burst analysis was applied to identify references that received rapidly increasing citations during specific periods, thereby indicating emerging topics and research frontiers. The dual-map overlay was used to visualize citation paths between citing and cited journals and to examine interdisciplinary knowledge flows.

The bibliometrix package was used for thematic evolution and topic trend analyses. Trend topic analysis, stacked time-series analysis of high-frequency keywords, time-slice heatmap analysis, and alluvial diagram analysis were performed to examine the temporal evolution of research hotspots. These analyses were used to examine temporal changes in keyword frequency and thematic relationships.

### Funding-agency analysis

Funding-agency information was extracted from the Funding Agency (FU) field of the Web of Science records. Agency names were standardized by merging abbreviations, spelling variants, and alternative forms of the same organization. When the same funding agency appeared more than once in a publication, it was counted only once for that publication. Funding-agency frequency was calculated as the number of publications acknowledging each agency. The corresponding percentages were calculated using both all included publications and publications with available funding-agency information as denominators.

### Integration of bibliometric output with dengue burden and vector suitability

Country-level research output was derived from the author affiliation addresses reported in the Web of Science records. A full-counting approach was used, whereby each country represented in a publication received one publication credit, irrespective of the number of participating authors or institutions from that country. Duplicate publication–country combinations were removed. Countries included in the epidemiological dataset but with no eligible publication were assigned a publication count of zero. Country names were standardized and linked across datasets using ISO 3166–1 alpha-3 codes.

Country-level dengue burden estimates were obtained from the Global Burden of Disease 2023 Results Tool [2]. The indicators included the age-standardized incidence rate (ASIR) and age-standardized disability-adjusted life-year (DALY) rate in 2023. For the DALY rate, the point estimate and corresponding lower and upper bounds of the 95% uncertainty interval (UI) were extracted for each country. Temporal changes in incidence were represented by country-specific estimates of the estimated annual percentage change (EAPC) in ASIR from 1990 to 2023. Countries with missing or non-finite EAPC values or with fewer than 5 years of data contributing to the estimate were excluded from the EAPC analysis. The socio-demographic index (SDI) for 2021 was used as a descriptive indicator of national socioeconomic development.

For the correlation analyses, the analytical sample was restricted to dengue-endemic countries. Because no single universally accepted official list of dengue-endemic countries was available, endemicity was operationally defined using the dengue risk categories presented in the CDC Yellow Book dengue risk maps [78]. Countries were included when frequent or continuous dengue risk was indicated at the national level or across a substantial part of the national territory. Countries with only focal subnational transmission, sporadic or uncertain risk, or no evidence of risk were excluded from the primary analysis. Country names were standardized using ISO 3166-1 alpha-3 codes, and all country-level classifications were manually reviewed. The classification criteria and individual country-level decisions are provided in Supplementary Table S1.

Climate-driven suitability for dengue vector transmission was characterized using index-P, a mechanistic suitability index based on the effects of temperature and humidity on mosquito and viral traits [3, 33]. Annual country-level index-P estimates were used to calculate the mean value over 1990–2022, which represented long-term climatic suitability for dengue vector transmission. The resulting estimates were linked to the bibliometric and epidemiological datasets using ISO 3166-1 alpha-3 codes.

For visualization, publication counts were transformed as log10(count + 1), while ASIR and DALY rates were transformed as log10(rate + 0.01). These transformations reduced right skewness and accommodated zero values. These transformations reduced right skewness and accommodated zero values. Associations between country-level publication output and ASIR, DALY rate, and EAPC were assessed using Spearman’s rank correlation coefficients among countries classified as dengue-endemic. The effective sample size was calculated and reported separately for each correlation after excluding countries with missing or non-finite values for either variable. The EAPC analysis additionally required a valid estimate based on at least five annual observations. Spearman’s correlation coefficients were calculated using the original values; logarithmic transformations were applied only for visualization.

To assess the robustness of the association to uncertainty in the GBD burden estimates, sensitivity analyses were performed by replacing the point estimate of the 2023 age-standardized DALY rate with the corresponding lower and upper bounds of its 95% UI. Spearman’s rank correlations between publication output and each alternative DALY estimate were calculated using the same set of 80 dengue-endemic countries.

To avoid constructing a composite score, dengue burden, long-term index-P, and eligible publication output were presented as separate indicators among dengue-endemic countries. Countries with no eligible publications were identified descriptively but were not interpreted as having no dengue research or as representing a definitive dengue-specific research gap.

## Results

### Publication trends

**Fig. 2:**
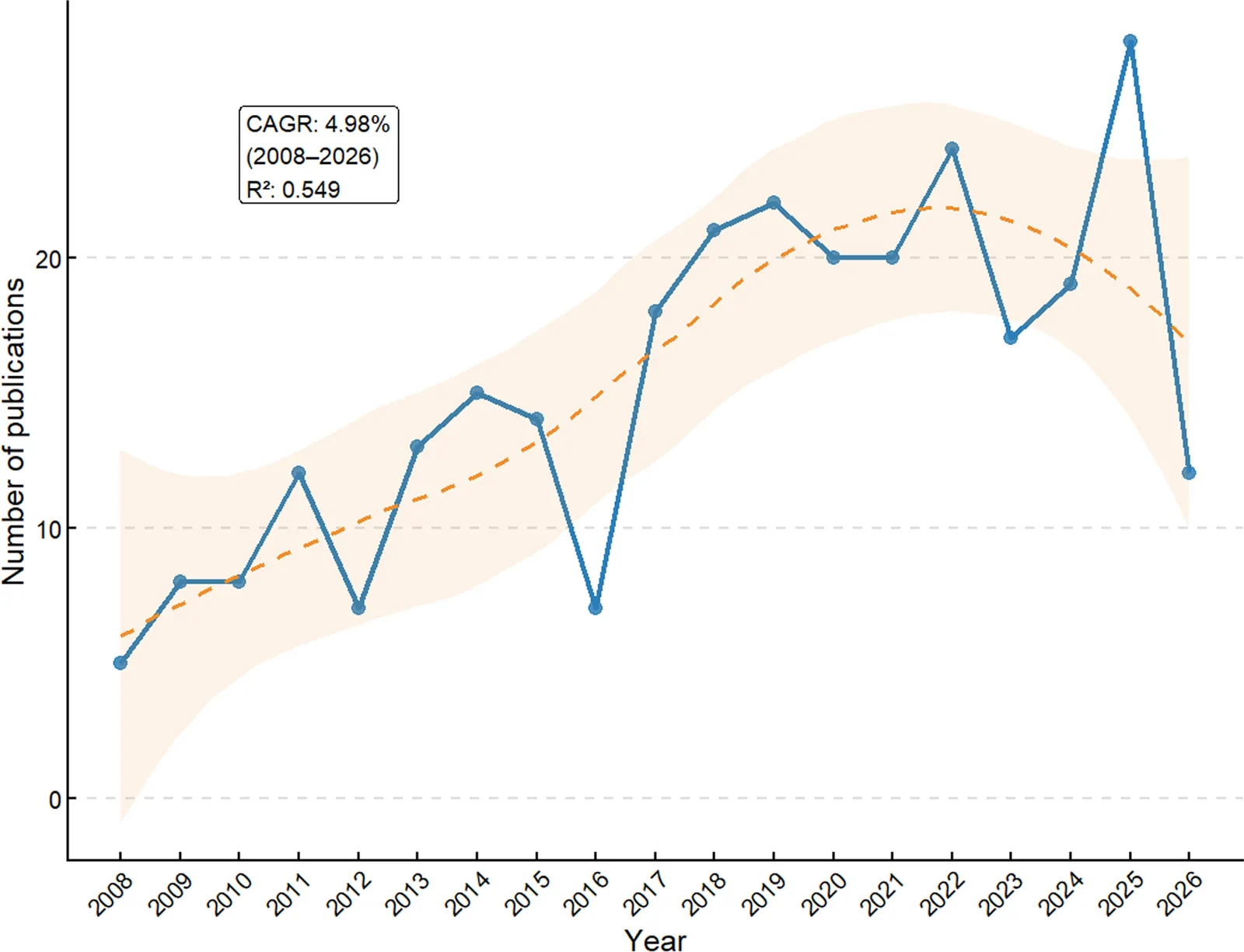
Annual publication output and trend from 2008 to 2026. The literature search was conducted on May 23, 2026; therefore, data for 2026 were incomplete

A total of 290 publications on dengue and extreme weather, published between 2008 and 2026, met the eligibility criteria. Annual publication output increased overall, although year-to-year fluctuations were observed (Fig. 2). Between 2008 and 2012, the number of publications ranged from 5 to 12 per year. It subsequently increased from 13 publications in 2013 to 22 in 2019 and remained comparatively high after 2020. The annual output reached 24 publications in 2022 and a maximum of 28 in 2025. Because the search was completed on 22 May 2026, the publication count for 2026 represented only a partial year and was not directly comparable with the counts for complete calendar years.

### Country and institutional analysis

The 290 publications involved authors from 66 countries and yielded 551 unique publication–country combinations under the full-counting approach. The United States contributed to 113 publications (39.0%), followed by China with 54 (18.6%), the United Kingdom with 47 (16.2%), Australia with 38 (13.1%), and Brazil with 25 (8.6%) (Table 1). Because each participating country received one publication credit, the sum of the country-level counts exceeded the total number of included publications.

**Table 1:** Leading countries contributing to research on dengue and extreme weather

Rank	Country	Publications(n)	Percentage(%)
1	United States	113	39.0
2	China	54	18.6
3	United Kingdom	47	16.2
4	Australia	38	13.1
5	Brazil	25	8.6
6	Singapore	17	5.9
7	Germany	15	5.2
8	Spain	13	4.5
9	France	12	4.1
10	Vietnam	11	3.8

Country-level publication output was calculated using a full-counting approach. Each publication was counted once for every participating country, regardless of the number of authors or institutions from that country. Percentages were calculated using all 290 included publications as the denominator. Because internationally co-authored publications were assigned to each participating country, the percentages were not mutually exclusive and may sum to more than 100%

**Fig. 3:**
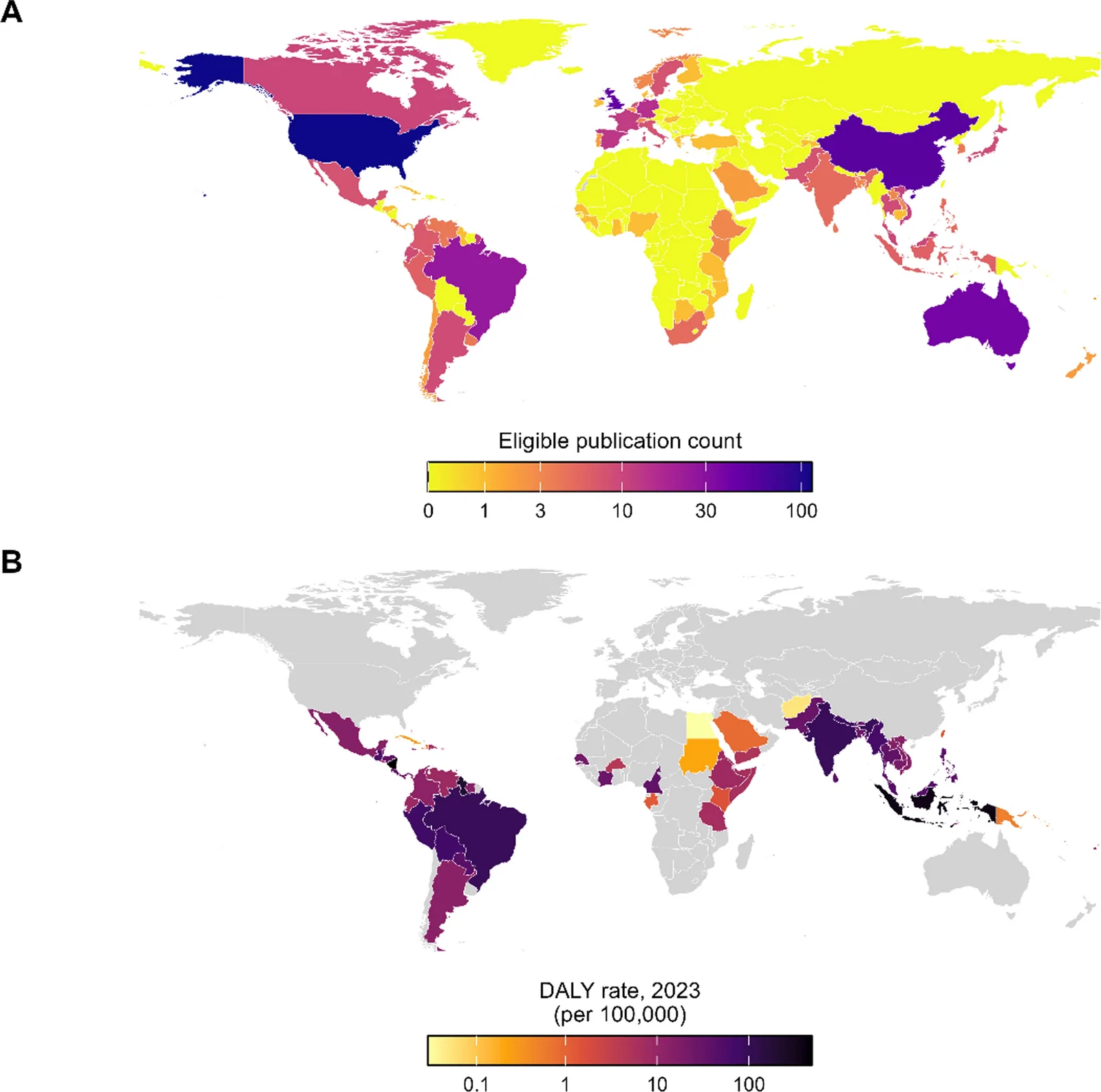
Global distribution of indexed research output and dengue burden. **A** Country-level number of eligible publications on dengue and extreme weather. Each publication–country combination was counted once, and countries represented in the epidemiological dataset but without eligible publications were assigned a publication count of zero. **B** Age-standardized dengue disability-adjusted life-year (DALY) rate in 2023 among countries classified as dengue-endemic. Non-endemic countries and countries without sufficient data are shown in gray. Country-specific point estimates of the 2023 age-standardized dengue DALY rate are displayed; the corresponding 95% UIs are provided in Supplementary Table S2

The geographic distribution of research output was uneven. The largest publication counts were observed in North America, Western Europe, East Asia, Oceania, and parts of South America and Southeast Asia (Fig. 3A). Dengue DALY rates varied substantially across endemic countries, with comparatively high rates observed in parts of Central America, the Caribbean, South and Southeast Asia, and sub-Saharan Africa (Fig. 3B).

**Fig. 4:**
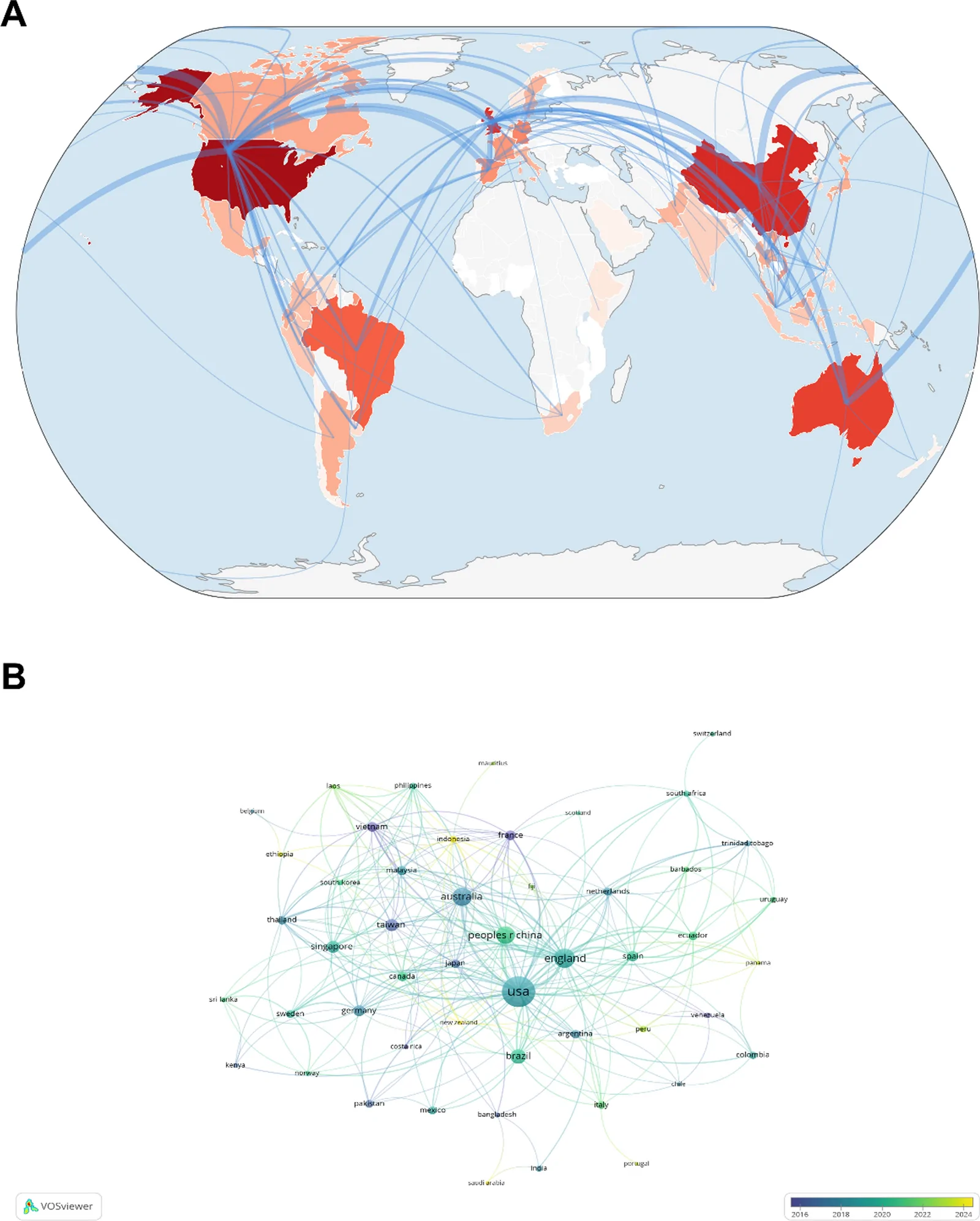
Country-level collaboration analysis

The country collaboration network was concentrated around a limited number of high-output countries (Fig. 4). The United States had collaborative links with China, Australia, the United Kingdom, Brazil, and several countries in Asia and Europe. China was also connected with the United States, Australia, the United Kingdom, and other research partners. Brazil, Singapore, Vietnam, Malaysia, Thailand, India, and Bangladesh were represented in the network, whereas many countries with lower publication counts had fewer links or were not represented.

**Fig. 5:**
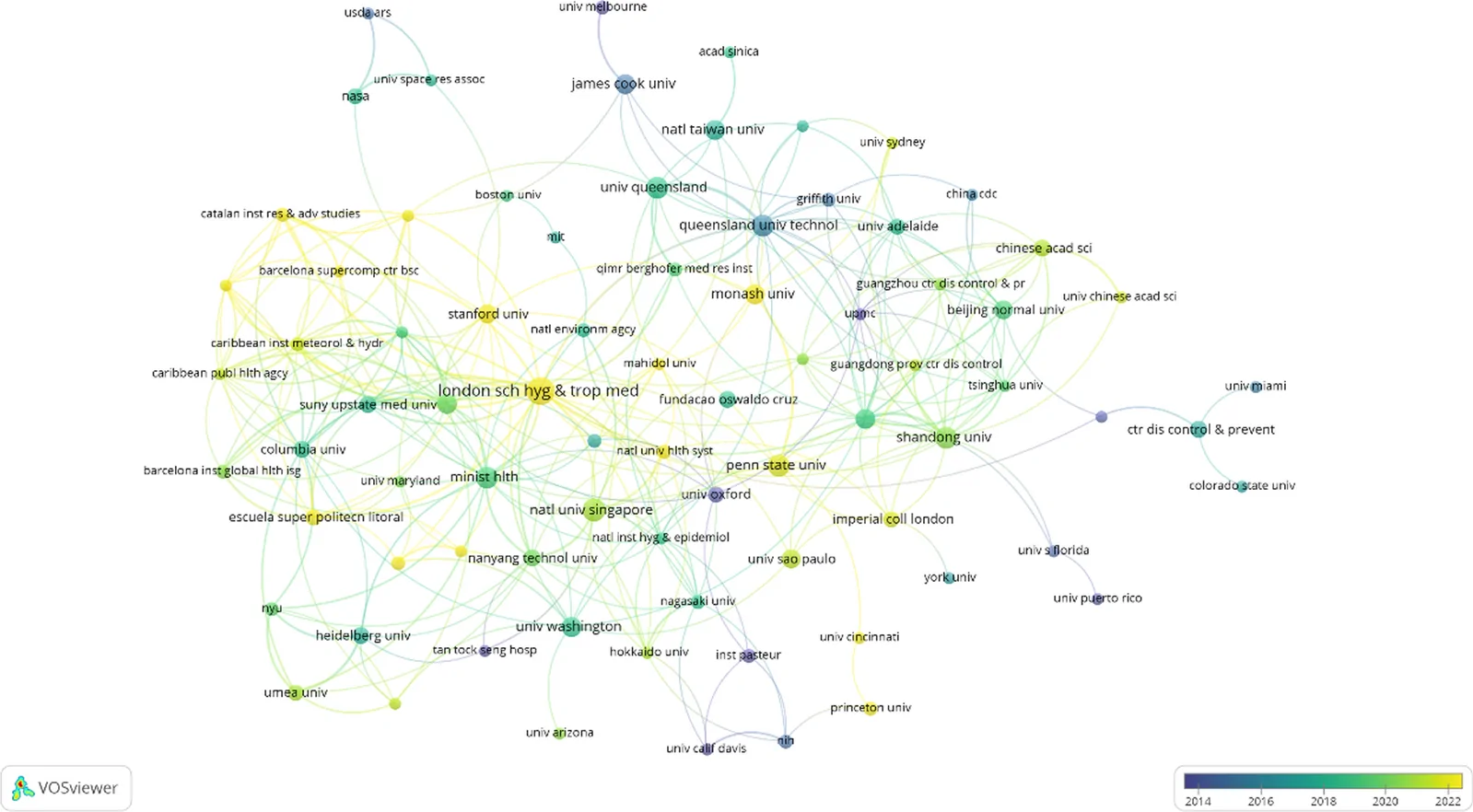
Visualization of institutional collaboration in research on dengue fever and extreme weather

At the institutional level, the University of London had the highest count, with 33 contributions, followed by the London School of Hygiene and Tropical Medicine with 30, the Pennsylvania Commonwealth System of Higher Education with 26, Pennsylvania State University with 25, and the National University of Singapore with 24. The institutional collaboration network contained several interconnected clusters (Fig. 5). The London School of Hygiene and Tropical Medicine had links with multiple institutions, while the National University of Singapore, Pennsylvania State University, Shandong University, the University of Queensland, the University of Sydney, and Queensland University of Technology were also prominent nodes.

### Funding-agency analysis

Funding-agency information was available for 222 of the 290 included publications (76.6%). The most frequently acknowledged funders were the National Institutes of Health (NIH; *n* = 25, 11.3% of publications with funding information), the National Science Foundation (NSF; *n* = 19, 8.6%), the National Natural Science Foundation of China (NSFC; *n* = 18, 8.1%), and the UK Medical Research Council (MRC; *n* = 16, 7.2%). The US Centers for Disease Control and Prevention (CDC) also ranked among the leading funders (*n* = 9, 4.1%).

### Alignment of research output with dengue burden and vector suitability

**Fig. 6:**
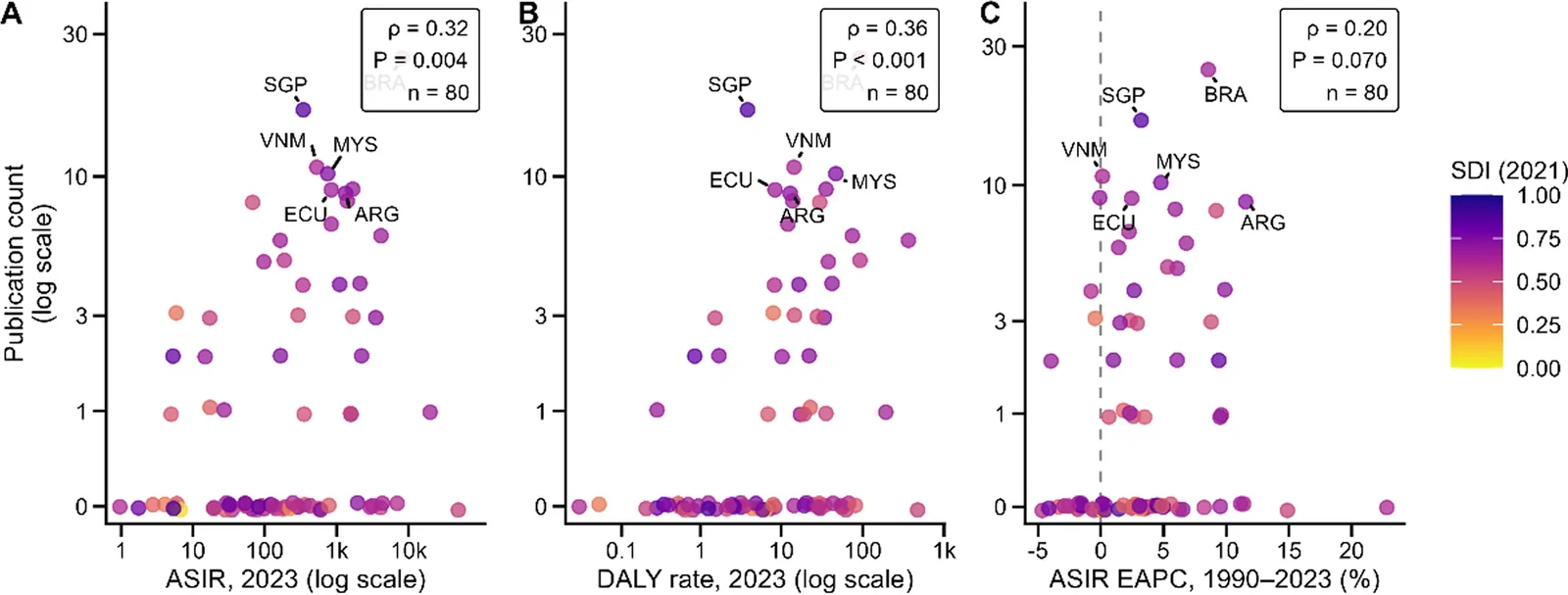
Associations between national research output and dengue disease burden among dengue-endemic countries. **A** Association between publication count and the age-standardized incidence rate (ASIR) in 2023. **B** Association between publication count and the disability-adjusted life-year (DALY) rate in 2023. **C** Association between publication count and the estimated annual percentage change (EAPC) in ASIR from 1990 to 2023. Spearman’s rank correlation coefficients were calculated using the original, untransformed values; logarithmic transformations were applied only for visualization in panels A and B and to publication counts in panel C. Each point represents one dengue-endemic country, and point color indicates the 2021 socio-demographic index (SDI). The effective sample size (n) is reported separately for each analysis

Among the 80 countries classified as dengue-endemic, country-level publication output was positively correlated with the ASIR in 2023 (Spearman’s ρ = 0.32, *P* = 0.004, *n* = 80) and the DALY rate in 2023 (ρ = 0.36, *P* < 0.001, *n* = 80). In contrast, the association between publication output and the EAPC in ASIR from 1990 to 2023 was weaker and did not reach statistical significance (ρ = 0.20, *P* = 0.070, *n* = 80) (Fig. 6). In sensitivity analyses accounting for uncertainty in the GBD burden estimates, the positive association between publication output and the 2023 DALY rate remained statistically significant when either the lower bound (Spearman’s ρ = 0.40, P < 0.001, n = 80) or the upper bound (ρ = 0.31, P = 0.005, n = 80) of the 95% UI was used, indicating that the direction of the association was robust to uncertainty in the GBD estimates (Supplementary Table S3). These findings indicated that research output was more closely aligned with the contemporary level of dengue burden than with its long-term temporal trend.

Descriptive comparison of the separate indicators showed that several dengue-endemic countries with comparatively high DALY rates had no eligible Web of Science-indexed publications in the present dataset. These countries were regarded as under-represented in the retrieved literature rather than as having no dengue-related research. Geographic mapping showed that indexed publication output was concentrated in a relatively small group of countries, whereas dengue burden remained heterogeneously distributed across endemic regions (Fig. 3).

**Fig. 7:**
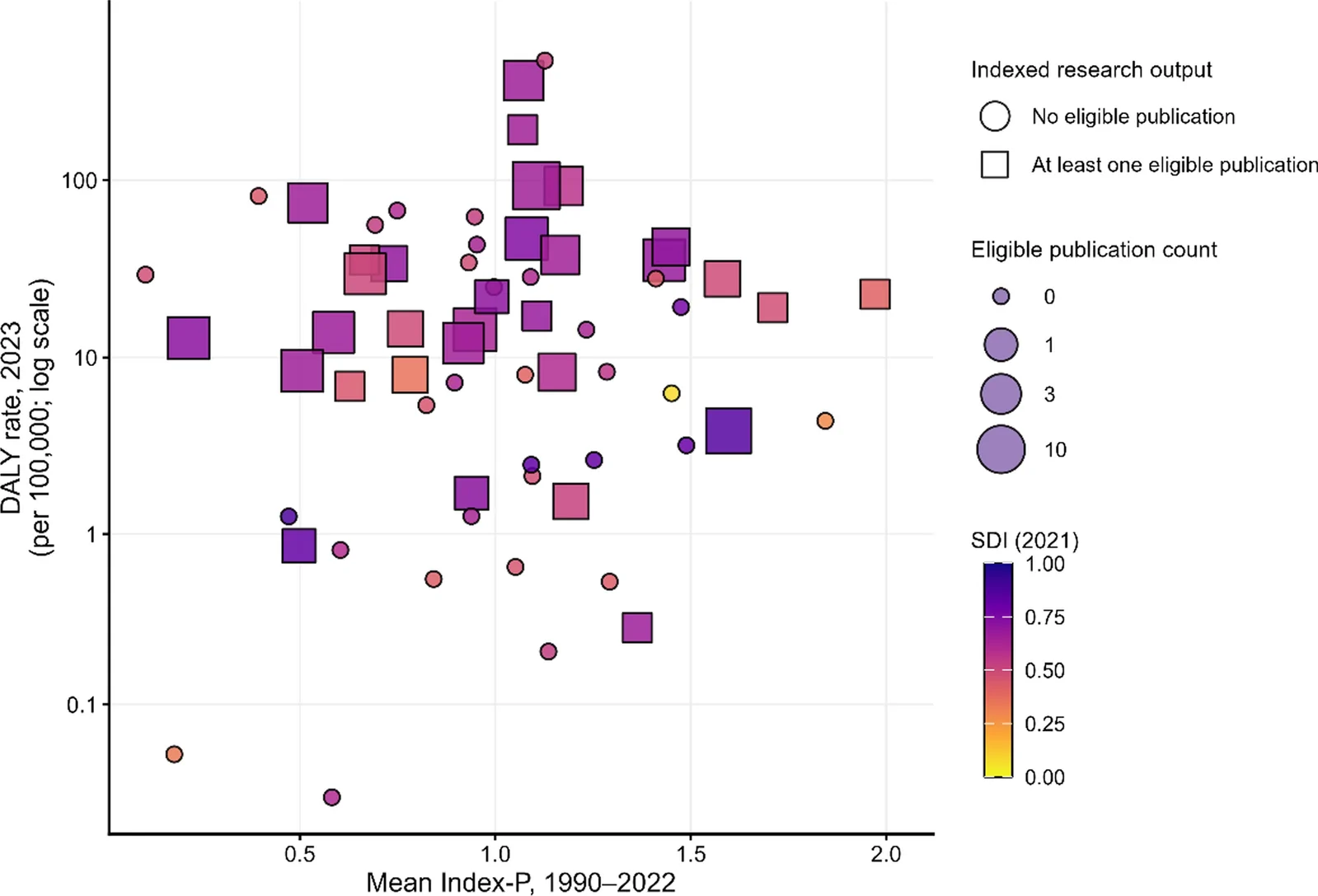
Dengue by Country-level dengue burden, climate-driven vector suitability, and indexed publication output among dengue-endemic countries. The x-axis represents the mean index-P over 1990–2022, and the y-axis represents the age-standardized dengue DALY rate in 2023. Point size indicates the number of eligible web of science-indexed publications, and point shape distinguishes countries with zero publications from those with at least one eligible publication. Point color represents the socio-demographic index in 2021. The analysis was restricted to dengue-endemic countries with complete DALY, index-P, publication, and SDI data (n = 63). Country-specific DALY point estimates are displayed; the corresponding 95% UIs are provided in Supplementary Table S3

The revised descriptive analysis presented long-term index-P, DALY rate, and eligible publication output as separate indicators among dengue-endemic countries (Fig. 7). Among the 63 dengue-endemic countries with complete data for the 2023 DALY rate, long-term index-P, and publication output, there was substantial heterogeneity in disease burden, climate-driven vector suitability, and indexed research activity (Fig. 7). Several countries combined relatively high DALY rates and high long-term climatic suitability with few or no eligible publications. However, these indicators were presented separately, without constructing a composite research-gap score or assigning countries to priority categories. It showed substantial cross-national heterogeneity in climate-driven vector suitability and disease burden. Several countries with comparatively high values for one or both indicators had no eligible Web of Science-indexed publications in the present dataset.

### Journals and co-cited journals

**Fig. 8:**
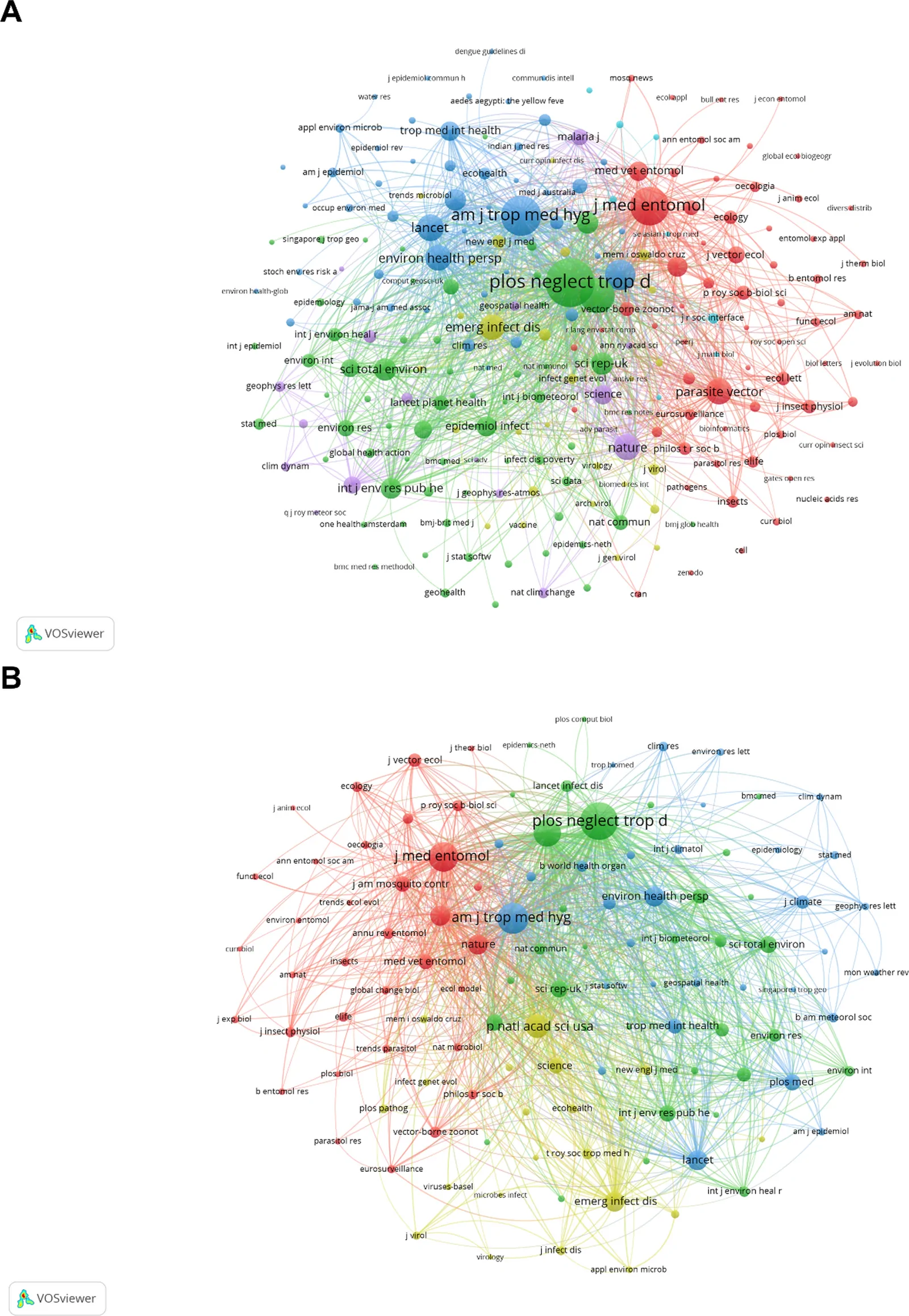
Visualization analysis of journals (**A)** and co-cited journals (**B**) in dengue fever and extreme weather research

The 290 publications were distributed across a range of journals. *PLOS Neglected Tropical Diseases* published the largest number of eligible articles (*n* = 28), followed by the International Journal of Environmental Research and Public Health and Parasites and Vectors (*n* = 14 each). Acta Tropica published 11 articles, while *the Journal of Medical Entomology and Scientific Report*s each published 9 (Table 2). The publishing-journal network was centered on *PLOS Neglected Tropical Diseases*, *the American Journal of Tropical Medicine and Hygiene*, *the Journal of Medical Entomology*, *PLOS ONE*, *Parasites and Vectors*, and *Scientific Reports* (Fig. 8A).

**Table 2:** Top 15 publishing journals and co-cited journals in research on dengue and extreme weather

Rank	Journal	Count	IF	Q	Co-cited Journal	Co-citation	IF	Q
1	PLOS Neglected Tropical Diseases	28	3.4	Q1	PLOS Neglected Tropical Diseases	874	3.4	Q1
2	International Journal of Environmental Research And Public Health	14	-	-	American Journal of Tropical Medicine And Hygiene	593	1.6	Q3
3	Parasites and Vectors	14	3.5	Q1	Journal of Medical Entomology	549	2.0	Q1
4	Acta Tropica	11	2.5	Q1	PLOS ONE	455	2.6	Q2
5	Journal Of Medical Entomology	9	2.0	Q1	Proceedings of the National Academy of Sciences of the United States of America	341	9.1	Q1
6	Scientific Reports	9	3.9	Q1	The Lancet	257	88.5	Q1
7	PLOS ONE	8	2.6	Q2	Environmental Health Perspectives	256	9.8	Q1
8	BMC Infectious Diseases	7	3.0	Q2	Parasites and Vectors	255	3.5	Q1
9	GeoHealth	7	3.8	Q1	Nature	246	48.5	Q1
10	The Lancet Planetary Health	6	21.6	Q1	Emerging Infectious Diseases	243	7.2	Q1
11	American Journal of Tropical Medicine and Hygiene	5	1.6	Q3	Scientific Reports	182	3.9	Q1
12	Science of the Total Environment	5	8.0	Q1	Science of the Total Environment	181	8.0	Q1
13	Environmental Research	4	7.7	Q1	Acta Tropica	170	2.5	Q1
14	Environmental Research Letters	4	5.6	Q1	PLOS Medicine	167	9.9	Q1
15	International Journal of Biometeorology	4	2.6	Q2	International Journal of Environmental Research and Public Health	162	-	-

“-” indicates that the journal has no current JCR Impact Factor or JCR quartile. Journal impact factors and quartiles were obtained from the 2025 Journal Citation Reports and correspond to the 2024 Journal Impact Factor reporting year. A dash indicates that no current Journal Impact Factor or JCR quartile was available

Among co-cited journals, *PLOS Neglected Tropical Diseases* ranked first with 874 co-citations, followed by the American Journal of Tropical Medicine and Hygiene with 593, the Journal of Medical Entomology with 549, and *PLOS ONE* with 455 (Table 2). Other frequently co-cited sources included the Proceedings of *the National Academy of Sciences of the United States of America, The Lancet, Environmental Health Perspectives, Nature,* and *Emerging Infectious Diseases*. The co-cited journal network showed close connections among journals in tropical medicine, medical entomology, infectious disease epidemiology, environmental health, and general science (Fig. 8B).

**Fig. 9:**
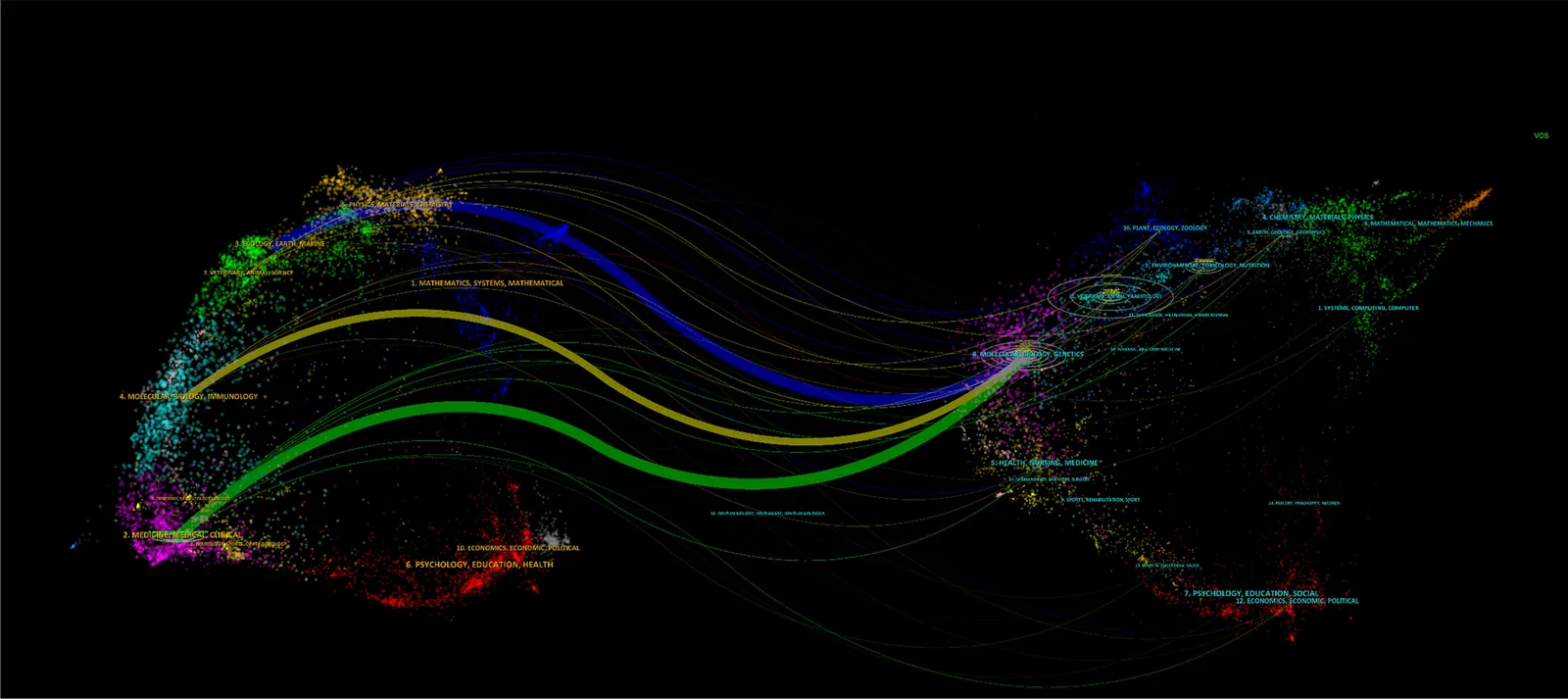
Dual-map overlay of journals on dengue, climate, and extreme weather research. The left side represents the disciplinary distribution of citing journals, while the right side represents that of cited journals. Colored curves indicate the main citation trajectories from citing disciplines to cited disciplines, with thicker curves representing more prominent citation paths

The dual-map overlay of journals further illustrated the disciplinary citation patterns in this field (Fig. 9). The citing journals were mainly distributed in disciplines such as Medicine, Medical, Clinical; Molecular Biology, Immunology; and Psychology, Education, Health, whereas the cited journals were concentrated primarily in Molecular Biology, Genetics; Health, Nursing, Medicine; and related disciplines. Several prominent citation trajectories connected the citing and cited journal clusters, indicating that research on dengue, climate, and extreme weather draws on knowledge across clinical medicine, molecular biology, and health-related disciplines. These citation paths demonstrate the interdisciplinary knowledge flow underlying this research field.

### Authors and co-cited authors

Rachel Lowe was the most productive author, with 11 publications, followed by Qiyong Liu with 9, Anna M. Stewart-Ibarra with 8, and Wenbiao Hu with 7. Wei Ma, Sadie J. Ryan, and Shilu Tong each contributed 6 publications, while Peng Bi, Mercy J. Borbor-Cordova, and Jue Tao Lim each contributed 5 (Table 3).

**Table 3:** Top 10 productive authors and co-cited authors in research on dengue and extreme weather

Rank	Author	Count	Co-cited Author	Co-citation
1	Rachel Lowe	11	Simon Hales	102
2	Qiyong Liu	9	Rachel Lowe	100
3	Anna M. Stewart-Ibarra	8	Michael A. Johansson	91
4	Wenbiao Hu	7	Samir Bhatt	77
5	Wei Ma	6	Bernard Cazelles	71
6	Sadie J. Ryan	6	Duane J. Gubler	71
7	Shilu Tong	6	Oliver J. Brady	68
8	Peng Bi	5	Erin A. Mordecai	65
9	Mercy J. Borbor-Cordova	5	Cory W. Morin	63
10	Jue Tao Lim	5	Dana A. Focks	56

**Fig. 10:**
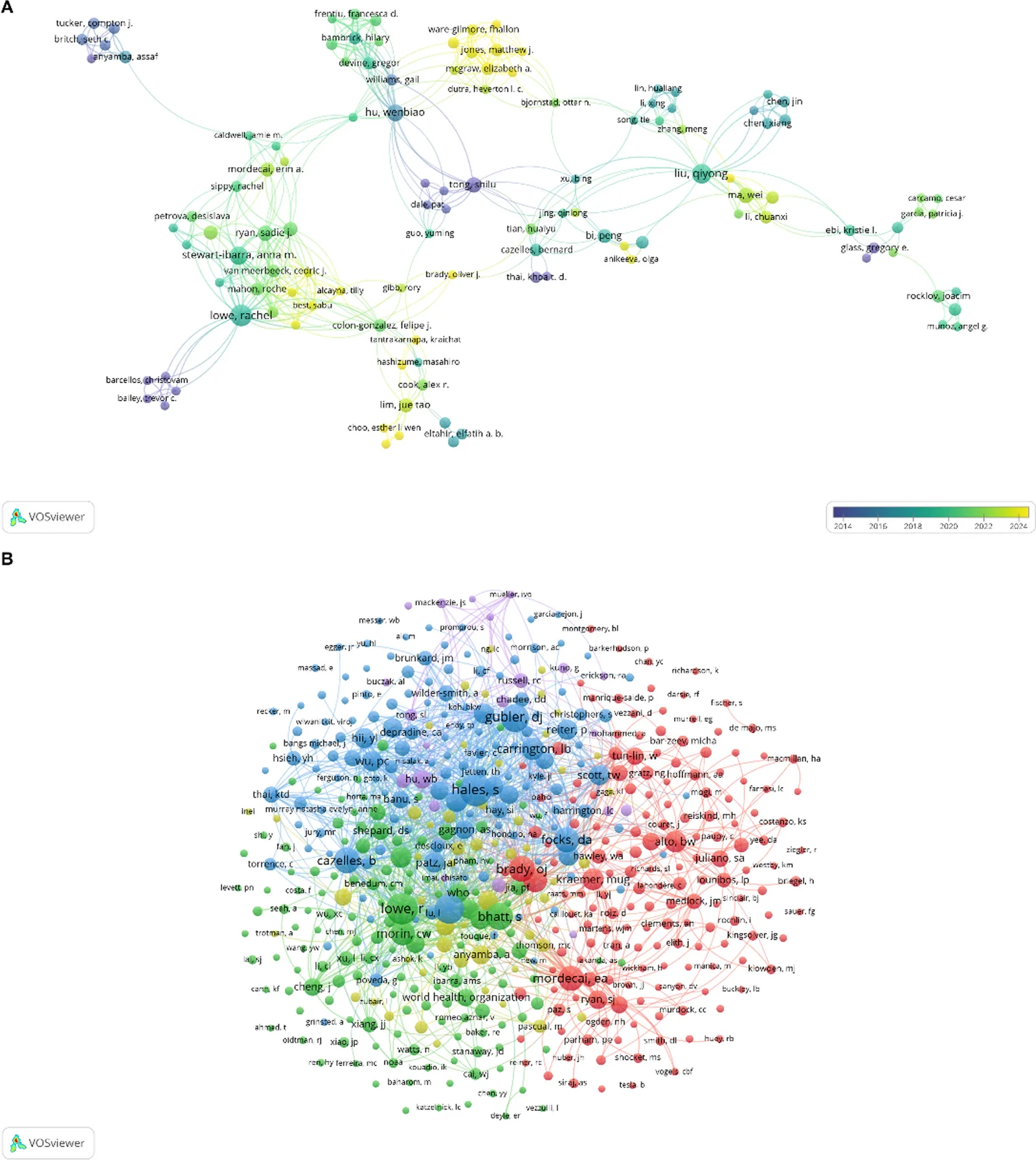
Visualization of authors (**A)** and co-cited authors (**B**) in research on dengue fever and extreme weather

The author collaboration network contained several distinct clusters (Fig. 10A). Rachel Lowe, Wenbiao Hu, Qiyong Liu, Anna M. Stewart-Ibarra, Sadie J. Ryan, and Shilu Tong were among the authors with relatively large nodes or multiple collaborative links. Connections were dense within several clusters but less extensive between some groups.

Simon Hales was the most frequently co-cited author, with 102 co-citations, followed by Rachel Lowe with 100, Michael A. Johansson with 91, and Samir Bhatt with 77. Bernard Cazelles and Duane J. Gubler each received 71 co-citations. Oliver J. Brady, Erin A. Mordecai, Cory W. Morin, and Dana A. Focks were also among the ten most frequently co-cited authors (Table 3). The co-cited author network showed connections among researchers working on climate-sensitive dengue transmission, vector ecology, global dengue burden, transmission modeling, and early warning (Fig. 10B).

### Co-cited references and citation bursts

**Fig. 11:**
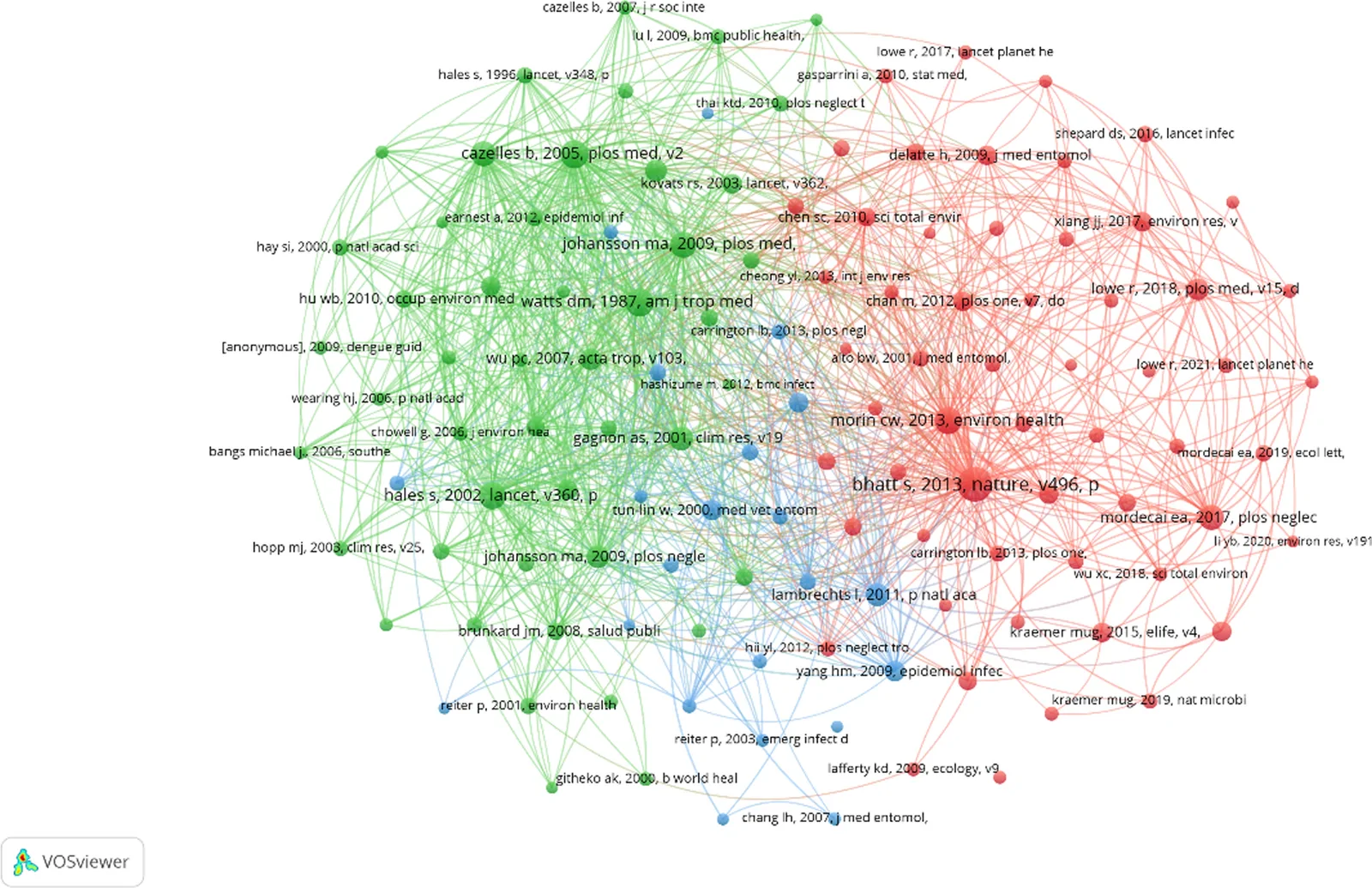
Visualization of co-cited references in research on dengue fever and extreme weather

The co-cited reference network contained three major thematic clusters (Fig. 11). The green cluster comprised studies of climate variability, meteorological factors, and dengue transmission dynamics. The red cluster focused on the global distribution and burden of dengue, climate change, Aedes-borne virus transmission, and projected disease risk, while the blue cluster covered vector ecology, mosquito biology, and environmental determinants of transmission. Because co-citation links connect references that are frequently cited together, the clusters represent related bodies of knowledge rather than simply groups of highly cited publications. The links among the three clusters indicate that research on dengue and extreme weather draws jointly on epidemiological evidence of disease burden, climatic determinants of transmission, mosquito biology and ecology, and spatial or mechanistic risk modeling. Thus, the network reveals how these different knowledge domains collectively support the development of climate-informed dengue risk assessment and prediction.

**Fig. 12:**
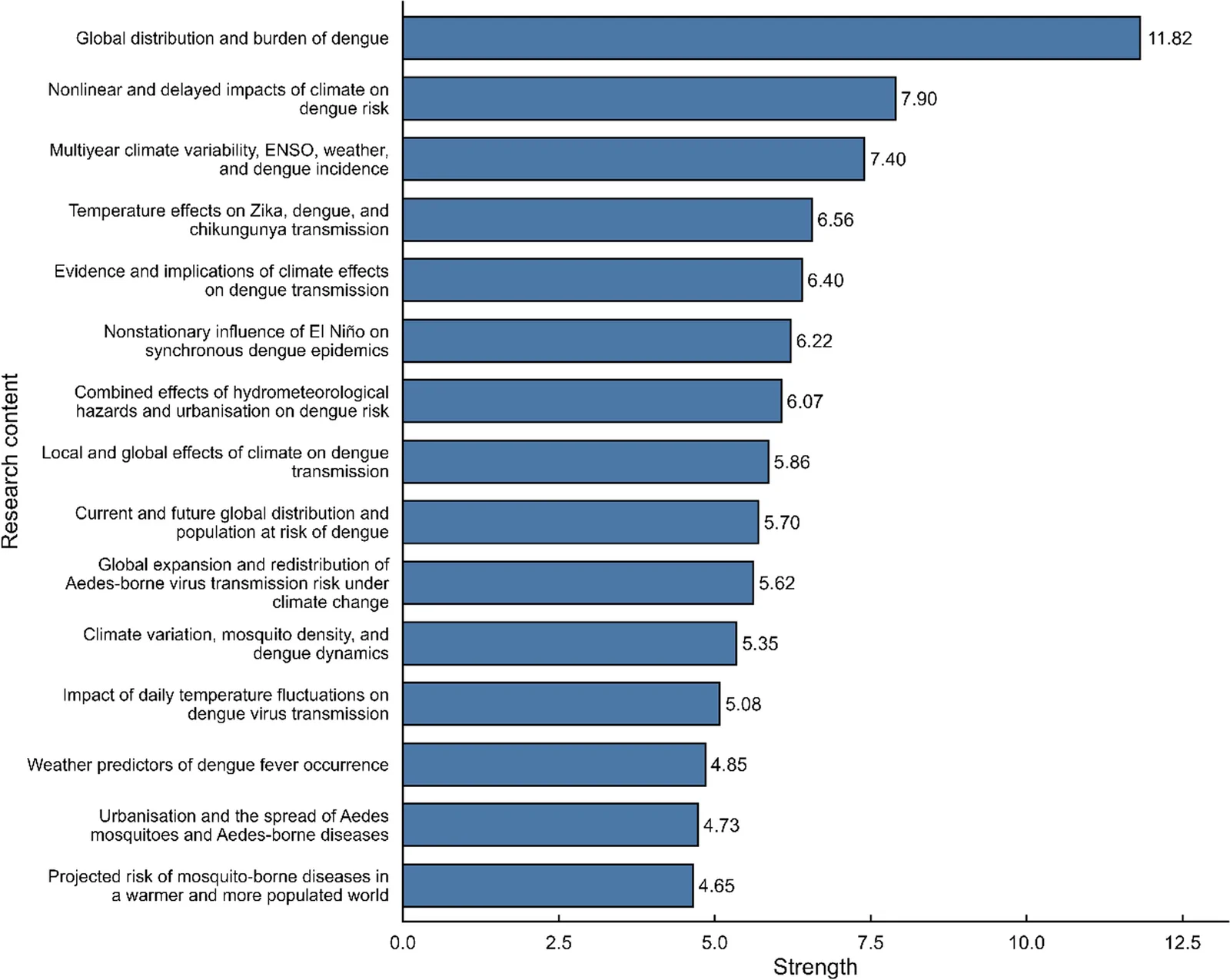
Main research contents of the top 15 references with the strongest citation bursts

**Fig. 13:**
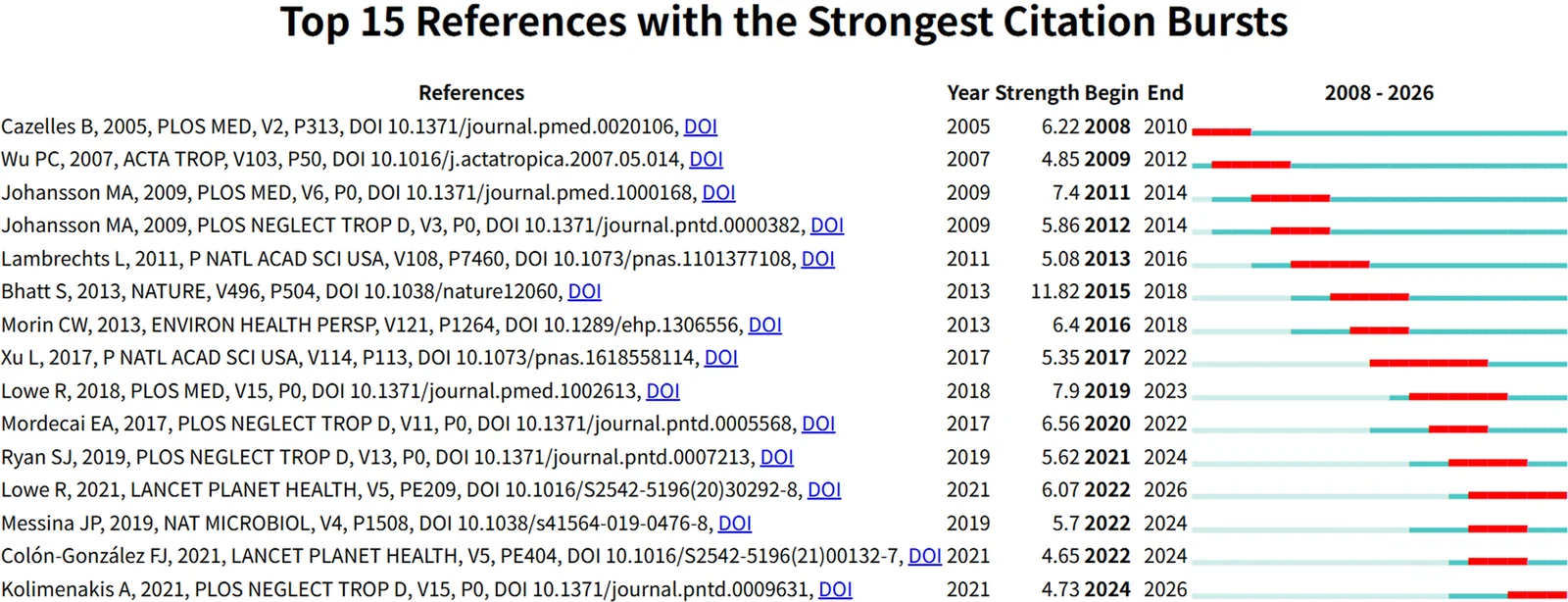
Top 15 references with the strongest citation bursts in research on dengue fever and extreme weather. The red bars indicate periods of high citation frequency

CiteSpace identified the 15 references with the strongest citation bursts during 2008–2026 (Fig. 12). The strongest burst concerned the global distribution and burden of dengue, with a burst strength of 11.82. Other prominent burst references addressed nonlinear and delayed climatic effects on dengue risk, multiyear climate variability, the El Niño–Southern Oscillation, temperature-dependent suitability for arbovirus transmission, hydrometeorological hazards, urbanization, and projected changes in the distribution of *Aedes*-borne virus risk. The topics and burst strengths of these references are summarized in Fig. 13 and Table 4.

**Table 4:** Main research contents of the 15 references with strong citation bursts

Rank	Strength	Main research content
1	11.82	Global distribution and burden of dengue
2	7.90	Nonlinear and delayed impacts of climate on dengue risk
3	7.40	Multiyear climate variability, ENSO, weather, and dengue incidence
4	6.56	Temperature effects on Zika, dengue, and chikungunya transmission
5	6.40	Evidence and implications of climate effects on dengue transmission
6	6.22	Nonstationary influence of El Niño on synchronous dengue epidemics
7	6.07	Combined effects of hydrometeorological hazards and urbanisation on dengue risk
8	5.86	Local and global effects of climate on dengue transmission
9	5.70	Current and future global distribution and population at risk of dengue
10	5.62	Global expansion and redistribution of Aedes-borne virus transmission risk under climate change
11	5.35	Climate variation, mosquito density, and dengue dynamics
12	5.08	Impact of daily temperature fluctuations on dengue virus transmission
13	4.85	Weather predictors of dengue fever occurrence
14	4.73	urbanization and the spread of Aedes mosquitoes and Aedes-borne diseases
15	4.65	Projected risk of mosquito-borne diseases in a warmer and more populated world

### Keyword-based research hotspots and thematic evolution

**Fig. 14:**
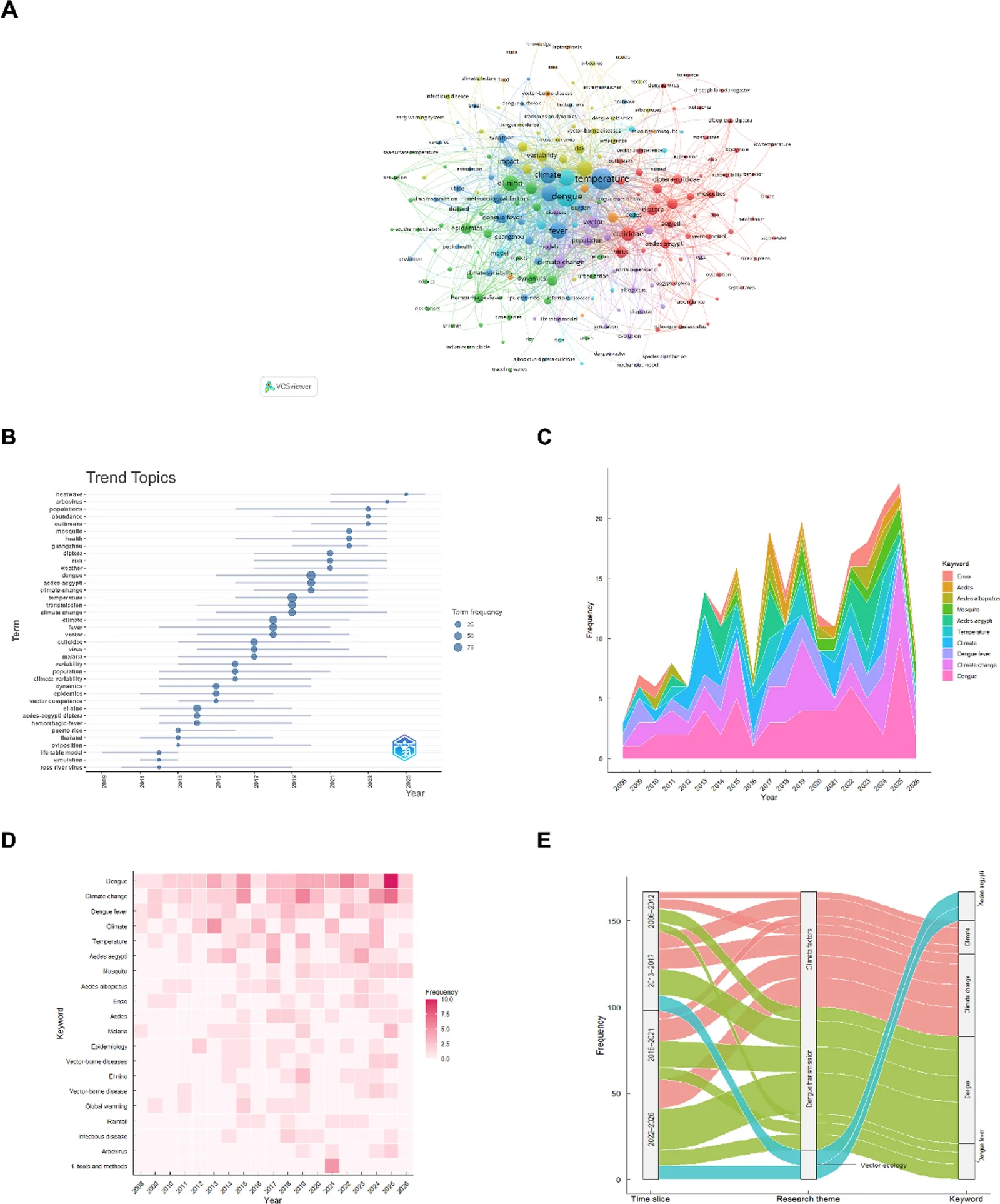
Keyword-based research hotspots and thematic evolution analysis. **A** Keyword co-occurrence clustering network. **B** Temporal evolution of trend topics. **C** Stacked time-series analysis of high-frequency keywords. **D** Time-slice heatmap of research field keywords. **E** Alluvial diagram of thematic evolution based on high-frequency keywords

The most frequent keywords were “dengue”, “climate”, “temperature”, “rainfall”, “Aedes aegypti”, “Aedes albopictus”, “dengue fever”, and “climate change” (Fig. 14A). The co-occurrence network grouped these terms into themes related to climatic factors, dengue transmission dynamics, vector ecology, and public health risk assessment.

The trend-topic analysis showed that rainfall, temperature, time-series analysis, and dengue fever were more prominent during the earlier years, whereas climate change, urbanization, population, Aedes, disease, and transmission appeared more frequently in later years (Fig. 14B). The frequencies of dengue, climate, temperature, rainfall, *Aedes aegypti*,and *Aedes albopictus *increased particularly after 2015 (Fig. 14C). The time-slice heatmap showed that dengue, climate change, dengue fever, climate, temperature, *Aedes aegypti*, and mosquito occurred consistently across multiple study periods (Fig. 14D).

The alluvial diagram showed temporal links between early themes concerning meteorological factors and dengue transmission and later themes involving climate change, Aedes vectors, mosquito ecology, and transmission risk (Fig. 14E).

## Discussion

### Main findings and publication trends

Three findings are particularly relevant. First, publication output increased after 2020 but remained concentrated in a limited number of countries and institutions. Second, among dengue-endemic countries, publication output showed modest positive associations with ASIR and DALY rate in 2023, whereas its association with the EAPC in ASIR was not statistically significant. Third, despite the observed associations with contemporary disease burden, several countries combined high dengue burden or climate-driven vector suitability with little or no eligible Web of Science-indexed publication output. By integrating bibliometric, epidemiological, and environmental indicators, this study identified geographic imbalances that would not be apparent from publication rankings or correlation analyses alone.

### Global contributions and collaboration patterns

Research output was concentrated in a relatively small group of countries, led by the United States, China, the United Kingdom, Australia, and Brazil (Table 1). The United States had the largest publication output and extensive international co-authorship links, while China also maintained collaborations with partners in Asia, Europe, North America, and Oceania (Fig. 4). The funding-agency analysis provides additional context for the leading contribution of the United States. The NIH and NSF were the two most frequently acknowledged funding agencies, and the CDC also ranked among the leading funders. This pattern suggests that sustained support from major US funding agencies may have contributed to the country’s high publication output. In addition, the United States has well-established biomedical and public health-research infrastructure, strong analytical capacity, and extensive international collaboration networks. Its high publication output may, therefore, include research conducted through multinational partnerships and studies involving dengue-endemic countries. Nevertheless, funding-acknowledgement frequency does not represent the amount of funding received, and the present analysis cannot establish that funding was the direct cause of the leading US publication output. Brazil, Singapore, Vietnam, and Malaysia were also prominent contributors, indicating that research activity may be influenced by both scientific capacity and the regional importance of dengue transmission [24, 25].

A similar concentration was observed at the institutional level. The leading institutions were primarily located in the United Kingdom, the United States, Singapore, China, and Australia, and the London School of Hygiene and Tropical Medicine occupied a well-connected position in the institutional collaboration network (Table 1; Fig. 5). However, several country and institutional clusters had relatively few connections, while some dengue-endemic regions remained under-represented in the indexed literature. This pattern points to a mismatch between research capacity and public health need, although the restriction to English-language publications indexed in the Web of Science may partly account for the observed imbalance [26, 27]. Partnerships between established research centers and institutions in dengue-affected settings could strengthen locally led evidence generation, context-specific risk assessment, and the development and implementation of dengue early warning systems [28, 29].

### Research–burden alignment and geographic heterogeneity

Linking bibliometric indicators with epidemiological estimates showed that, among dengue-endemic countries, indexed publication output was positively associated with both ASIR and DALY rate in 2023, although the magnitudes of these associations were modest (Fig. 6). This suggests that countries with a greater contemporary dengue burden generally received more research attention, but disease burden alone did not fully explain the geographic distribution of research output. In contrast, publication output was not significantly associated with the EAPC in ASIR from 1990 to 2023, indicating that countries experiencing more rapid increases in incidence did not necessarily receive correspondingly greater research attention. The positive associations with contemporary burden, therefore, do not eliminate the broader research–burden mismatch, particularly because several high-burden countries had few or no eligible Web of Science-indexed publications. Similar imbalances have been reported in global health research, where scientific activity is often concentrated in countries with greater research capacity rather than distributed solely according to health need [30]. However, disease burden is only one determinant of research activity; national funding, scientific infrastructure, international collaboration, institutional priorities, market incentives, and the feasibility of conducting research also influence publication output [31].

Several dengue-endemic countries, including India, Indonesia, Brazil, Bangladesh, the Philippines, and Thailand, have experienced a substantial dengue burden [1, 2]. However, their contributions to global research output are not always proportional to their disease burden, as indicated by our findings. Several dengue-endemic countries had substantial dengue burden but few or no eligible publications in the present dataset. Differences in funding and research personnel may contribute to this pattern, as low-income countries receive a limited share of international health-research funding and have fewer health researchers per capita than high-income countries [30–32]. Database coverage is another likely contributor. The restriction to English-language records indexed in the Web of Science Core Collection may have excluded locally conducted studies published in regional journals or other languages. Countries with no eligible publications should, therefore, be regarded as under-represented in the dataset rather than as having no dengue-related research.

The interpretation of dengue burden estimates requires particular caution in sub-Saharan Africa, especially in parts of West and Central Africa. Limited surveillance coverage, restricted access to laboratory confirmation, under-reporting, and potential misclassification with other febrile illnesses mean that GBD estimates for these settings may rely substantially on model-based extrapolation and may have wide uncertainty intervals. In the present study, the positive association between publication output and the 2023 DALY rate remained statistically significant when either the lower bound (Spearman’s ρ = 0.40, P < 0.001, n = 80) or the upper bound (ρ = 0.31, P = 0.005, n = 80) of the GBD 95% uncertainty interval was used. The consistent direction of these associations indicates that the overall country-level finding was robust to the reported uncertainty intervals. Nevertheless, this sensitivity analysis does not eliminate uncertainty arising from sparse underlying surveillance data, diagnostic limitations, or the assumptions used in the GBD modeling framework.

The separate assessment of long-term index-P, dengue burden, and eligible publication output showed that some dengue-endemic countries with high climate-driven vector suitability or substantial disease burden were under-represented in the present Web of Science-based dataset (Fig. 7). Index-P estimates the contribution of temperature and humidity to the suitability of local conditions for mosquito-borne virus transmission [33]. It does not represent observed dengue transmission, which also depends on vector distribution, population immunity, human mobility, urban infrastructure, surveillance practices, vector-control interventions, and access to health care. The revised figure is, therefore, intended as a descriptive comparison of separate country-level indicators rather than as a ranking of national dengue risk or research need.

Estimates reported by Bhatt et al. [65] and Messina et al. [44] provide complementary evidence regarding dengue infection occurrence, environmental suitability, and populations living in areas at risk. However, their outcomes, reference periods, and modeling frameworks differ from the age-standardized DALY rates used in the present study and, therefore ,do not provide directly interchangeable country-level burden estimates. Accordingly, low indexed publication output in parts of West and Central Africa should not be interpreted solely as evidence of inadequate research investment; it may also reflect genuine scientific uncertainty regarding the magnitude and geographic distribution of dengue burden in these settings.

Among the 63 dengue-endemic countries with complete data, the separate assessment of long-term index-P, dengue burden, and eligible publication output showed that some countries with relatively high climate-driven vector suitability or substantial GBD burden estimates were under-represented in the present Web of Science-based dataset (Fig. 7). Index-P estimates the contribution of temperature and humidity to the suitability of local conditions for mosquito-borne virus transmission [33]. It does not represent observed dengue transmission, which also depends on vector distribution, population immunity, human mobility, urban infrastructure, surveillance practices, vector-control interventions, and access to health care. The revised figure is, therefore, intended as a descriptive comparison of separate country-level indicators rather than as a ranking of national dengue risk or research need.

In countries where high estimated burden or climatic suitability coincides with limited indexed research, strengthening locally led surveillance and research capacity may be particularly valuable. Relevant measures could include improving laboratory and epidemiological surveillance, supporting research training, expanding access to data and analytical resources, and developing equitable international partnerships. These measures are consistent with the World Health Organization’s emphasis on collaborative surveillance, laboratory capacity, vector control, research, and innovation in addressing dengue and other Aedes-borne arboviruses [34].

### Interdisciplinary knowledge structure

The journal distribution highlights the interdisciplinary nature of research on dengue and extreme weather. The leading publication sources covered tropical medicine, infectious diseases, medical entomology, environmental health, and public health, including PLOS Neglected Tropical Diseases, International Journal of Environmental Research and Public Health, Parasites and Vectors, Acta Tropica, Journal of Medical Entomology, and Scientific Reports (Table 2). The field, therefore, draws on several related disciplines rather than developing within a single research domain [27, 35].

The co-citation results showed an even broader intellectual base. In addition to specialist journals in tropical medicine and medical entomology, frequently co-cited sources included Proceedings of the National Academy of Sciences of the United States of America, The Lancet, Environmental Health Perspectives, Nature, and Emerging Infectious Diseases (Table 2). The journal networks and dual-map overlay similarly identified citation links connecting medicine and public health with environmental science, ecology, and the biomedical sciences (Figs. 8 and 9). These patterns reflect the combination of epidemiological evidence, climate-related exposure assessment, vector ecology, and public health risk analysis in this research area [36–39].

The author networks were consistent with this disciplinary breadth. Productive and frequently co-cited researchers represented expertise in dengue epidemiology, climate variability, vector biology, disease modeling, and early warning systems (Table 3) [40]. Although several established research groups were visible in the collaboration network, links between some clusters remained limited (Fig. 10A). The co-citation network was centered on climate-sensitive transmission, mosquito ecology, global dengue burden, mathematical modeling, and public health risk assessment (Fig. 10B). Together, these structures show that the field is supported by contributions from tropical medicine, infectious disease epidemiology, environmental health, climate science, vector ecology, and quantitative modeling [41, 42].

### Knowledge base of the field

Co-citation analysis identifies references that are frequently cited together and thereby maps the shared intellectual foundations and conceptual relationships within a research field [43]. Its value extends beyond identifying highly cited publications: it can distinguish major bodies of evidence, reveal connections between disciplines, and show how different research traditions contribute to the development of a field. It may also help identify weakly connected themes and areas in which greater interdisciplinary integration is needed. In this study, the co-citation network connected four closely related knowledge domains: the global distribution and burden of dengue, climate-sensitive transmission, Aedes mosquito biology and ecology, and spatial or mechanistic modeling (Fig. 11).

Studies of temperature, rainfall, humidity, and the El Niño–Southern Oscillation established the sensitivity of dengue transmission to climatic conditions [45–47]. Research on *Aedes aegypti*, *Aedes albopictus*, vector competence, and environmental suitability linked these population-level associations to mosquito biology and ecology [48, 49]. The co-citation structure, therefore, reflects a close connection among epidemiological patterns, meteorological exposures, and the biological mechanisms underlying dengue transmission.

More recent modeling studies have combined these components to estimate transmission suitability, identify high-risk areas, and project changes in the distribution of dengue and other Aedes-borne viruses under climate change [50–52]. Such models also provide a methodological basis for risk assessment and climate-informed early warning. Rather than indicating a simple replacement of earlier research topics, the network suggests increasing integration of epidemiological, entomological, climatic, and modeling evidence [53]. The benefit of the co-citation network is, therefore, that it reveals how these bodies of evidence are intellectually connected and collectively support the explanation of climate-sensitive dengue transmission, the projection of future risk, and the translation of research findings into surveillance and early warning strategies.

### Research hotspots and emerging frontiers

Keyword and thematic analyses identified climate, temperature, rainfall, *Aedes aegypti*, *Aedes albopictus, *and climate change as persistent topics (Fig. 14). Earlier studies more often examined associations between individual meteorological variables and dengue incidence, whereas later work increasingly incorporated climate change, urbanization, population exposure, and spatial transmission risk [54–59].

The continued prominence of vector-related terms reflects the biological pathway through which weather affects dengue transmission. Temperature and moisture influence mosquito development, survival, biting activity, and viral replication, but their effects depend on vector presence and local environmental conditions [60, 61]. Combining epidemiological observations with entomological and climatic data is, therefore, important for distinguishing climatic suitability from observed transmission.

Citation bursts highlighted growing interest in nonlinear and delayed climatic effects, ENSO, hydrometeorological hazards, and the projected redistribution of Aedes-borne virus risk (Figs. 12 and 13) [62–65]. The increasing emphasis on prediction and early warning reflects a practical need to translate climate information into preparedness and vector-control decisions [28, 29, 66–69].

### Limitations

This study has several limitations. The bibliometric dataset was restricted to English-language articles and reviews indexed in the Web of Science Core Collection. Relevant studies available only through Scopus, PubMed, Embase, Google Scholar, regional databases, or non-English-language journals were, therefore, not captured. Because dengue is concentrated in tropical and subtropical regions, this restriction may have disproportionately excluded locally published research and underestimated the contributions of some dengue-endemic countries. Accordingly, the absence of eligible publications should be interpreted as under-representation within the dataset rather than evidence that no relevant research was conducted in a country.

The network and thematic results were also dependent on the analytical methods and parameter settings. CiteSpace, VOSviewer, and bibliometrix differ in data processing, network construction, clustering, and visualization, and alternative thresholds or algorithms could produce somewhat different network structures and thematic groupings [70–72]. Citation-based indicators are additionally subject to time-lag bias, as recently published studies have had less time to accumulate citations. The 2026 data covered only the period up to 22 May and consequently did not represent a complete publication year [73, 74].

The country-level integration introduced further constraints. Cumulative publication output from 2008 to May 2026 was compared with dengue burden estimates for 2023 and long-term index-P estimates for 1990–2022; these indicators, therefore, covered different time periods. Country-level publication counts were not normalized by population size, total national scientific output, or health-research output, because consistent denominators were not available for all included countries and territories. The observed publication counts, therefore, represent coverage in the present Web of Science-based dataset rather than dengue-specific research investment or national research capacity. Countries with no eligible publications may still have produced relevant research in regional databases, non-indexed journals, languages other than English, surveillance reports, or other forms of grey literature. The funding-agency analysis was based on acknowledgement frequency rather than funding amounts. Funding information was unavailable for 68 publications, and the presence of a funding acknowledgement does not indicate the level of financial support provided by an agency. The results should, therefore, be interpreted as a description of acknowledged funding patterns rather than evidence of a causal relationship between funding and publication output. Moreover, index-P represents climate-driven suitability for transmission and does not capture all determinants of observed dengue occurrence, such as vector distribution, population immunity, human mobility, urban infrastructure, surveillance practices, vector-control interventions, and access to health care. The combined visual presentation of these indicators was intended for descriptive comparison and should not be interpreted as a definitive ranking of national dengue risk or research investment need.

Finally, bibliometric indicators describe publication activity, citation relationships, collaboration patterns, and thematic development but do not directly assess the methodological quality, causal validity, or public health impact of individual studies [75, 76]. The ecological correlations reported here likewise cannot establish causal relationships between research output, dengue burden, and vector suitability. Systematic reviews, meta-analyses, and country-specific empirical studies are needed to evaluate the underlying evidence and clarify how extreme weather affects dengue transmission in different settings [77].

## Conclusions

Research on dengue and extreme weather expanded between 2008 and 2026 but remained concentrated in a limited number of countries and institutions. The literature increasingly connected climate change, vector ecology, transmission modeling, risk prediction, and early warning, reflecting greater integration of epidemiological, entomological, and climatic evidence.

Country-level publication output was not significantly associated with dengue ASIR or DALY rate in 2023, although a weak positive association was observed with the EAPC in ASIR from 1990 to 2023. Several countries had high dengue burden or climate-driven vector suitability but little or no eligible Web of Science-indexed publication output. These results identify geographic imbalances in the indexed literature and may inform further assessment of research capacity, surveillance needs, and international partnerships. Country-level publication output showed modest positive associations with dengue ASIR and DALY rate in 2023 among dengue-endemic countries, whereas its association with the EAPC in ASIR was not statistically significant.

## Supplementary Information

Additional file 1.

## Data Availability

The datasets used in this study are publicly available from established repositories. Global Burden of Disease (GBD) 2023 dengue estimates were obtained from the Global Health Data Exchange (GHDx) of the Institute for Health Metrics and Evaluation (IHME). Historical climate data were obtained from the ERA5-Land reanalysis dataset provided by the Copernicus Climate Data Store. Future climate projection data were obtained from the Coupled Model Intercomparison Project Phase 6 (CMIP6). Socio-demographic data were obtained from publicly available IHME and World Bank databases. The processed datasets and analytical code generated during the current study are available from the corresponding author upon reasonable request.

## Abbreviations

DENV: Dengue virus
ENSO: El Niño–Southern Oscillation
PRISMA: Preferred Reporting Items for Systematic Reviews and Meta-Analyses
WoSCC: Web of Science Core Collection
