# Supplementary material for: Global disparities in dengue, climate, and extreme weather research: a bibliometric analysis of alignment with disease burden and climate-driven vector suitability
Source: Trop Med Health. 2026 Nov 13;54:238. doi: 10.1186/s41182-026-01110-6 (PMC13760753; doi:10.1186/s41182-026-01110-6)
Supplement: Supplementary file 1 [file 41182_2026_1110_MOESM1_ESM.docx]

**Supplementary material**

**Supplementary contents**

1. **Supplementary Methods S1. Search strategy and literature retrieval**
2. **Supplementary Table S1. Country-level dengue transmission classification and inclusion in the primary correlation analysis**
3. **Supplementary Table S2.** **Country-level publication output, mean Index-P, and age-standardized dengue DALY rates with 95% uncertainty intervals among 80 dengue-endemic countries and territories.**
4. **Supplementary Table S3. Sensitivity analyses of the association between country-level publication output and alternative estimates of the 2023 age-standardized dengue DALY rate among 80 dengue-endemic countries and territories.**
5. **Supplementary Table S4. Bibliographic information and reasons for exclusion of records during title and abstract screening**

**Supplementary Methods S1. Search strategy and literature retrieval**

**Publications were retrieved from the Web of Science Core Collection on May 22, 2026.**

TS=(

dengue OR "dengue fever" OR "dengue virus" OR DENV

OR "Aedes aegypti" OR "Aedes albopictus"

)

AND TS=(

"extreme weather" OR "extreme climate" OR "climate extreme*"

OR "extreme event*" OR "weather extreme*" OR "climatic extreme*"

OR heatwave OR "heat wave*" OR "high temperature*" OR "extreme heat" OR "heat stress"

OR "cold wave*" OR "cold spell*" OR "low temperature*" OR "extreme cold" OR "cold surge"

OR "heavy rainfall" OR "extreme precipitation" OR "intense precipitation"

OR rainstorm* OR downpour* OR "torrential rain*"

OR flood* OR flooding OR inundation OR "storm surge"

OR typhoon* OR cyclone* OR hurricane* OR storm*

OR drought* OR "dry spell*" OR aridity

OR "dust storm*" OR sandstorm*

OR ENSO OR "El Nino" OR "El Niño" OR "La Nina" OR "La Niña"

)

AND TS=(

risk OR incidence OR outbreak* OR transmission OR association

OR correlate* OR impact OR effect* OR epidemic* OR cases

OR burden OR prevalence OR occurrence

.

| **Supplementary Table S1.** Country-level dengue transmission classification and inclusion in the primary correlation analysis | | | | | | | |
| --- | --- | --- | --- | --- | --- | --- | --- |
| Country | ISO3 code | CDC dengue risk classification | Classification criterion | Subnational heterogeneity | Primary analysis | National_endemicity_basis | Reviewer note |
| Afghanistan | AFG | Frequent or continuous risk | Evidence of >10 dengue cases in at least 3 of the previous 10 years | No | Included | CDC frequent or continuous risk classification |  |
| Albania | ALB | Needs manual review | Not individually and unambiguously classified in the CDC text description | No | Excluded | Not included in the primary national-level endemic-country analysis |  |
| Algeria | DZA | Needs manual review | Not individually and unambiguously classified in the CDC text description | No | Excluded | Not included in the primary national-level endemic-country analysis |  |
| American Samoa | ASM | Frequent or continuous risk | Evidence of >10 dengue cases in at least 3 of the previous 10 years | No | Included | CDC frequent or continuous risk classification |  |
| Andorra | AND | Sporadic or uncertain risk | Evidence of at least 1 locally acquired dengue case during the previous 10 years | No | Excluded | Not included in the primary national-level endemic-country analysis |  |
| Angola | AGO | Sporadic or uncertain risk | Evidence of at least 1 locally acquired dengue case during the previous 10 years | No | Excluded | Not included in the primary national-level endemic-country analysis |  |
| Antigua and Barbuda | ATG | Frequent or continuous risk | Evidence of >10 dengue cases in at least 3 of the previous 10 years | No | Included | CDC frequent or continuous risk classification |  |
| Argentina | ARG | Needs manual review | Not individually and unambiguously classified in the CDC text description | Yes | Included | Frequent or continuous risk across a substantial national area | CDC indicates no evidence of risk in southern Argentina, while risk may occur in northern areas; national classification requires review. |
| Armenia | ARM | Needs manual review | Not individually and unambiguously classified in the CDC text description | No | Excluded | Not included in the primary national-level endemic-country analysis |  |
| Australia | AUS | Needs manual review | Not individually and unambiguously classified in the CDC text description | Yes | Excluded | Frequent or local transmission restricted to a focal subnational area | CDC indicates frequent or continuous risk in parts of northeastern Australia; national classification requires review. |
| Austria | AUT | Needs manual review | Not individually and unambiguously classified in the CDC text description | No | Excluded | Not included in the primary national-level endemic-country analysis |  |
| Azerbaijan | AZE | Needs manual review | Not individually and unambiguously classified in the CDC text description | No | Excluded | Not included in the primary national-level endemic-country analysis |  |
| Bahamas | BHS | Frequent or continuous risk | Evidence of >10 dengue cases in at least 3 of the previous 10 years | No | Included | CDC frequent or continuous risk classification |  |
| Bahrain | BHR | Needs manual review | Not individually and unambiguously classified in the CDC text description | No | Excluded | Not included in the primary national-level endemic-country analysis |  |
| Bangladesh | BGD | Frequent or continuous risk | Evidence of >10 dengue cases in at least 3 of the previous 10 years | No | Included | CDC frequent or continuous risk classification |  |
| Barbados | BRB | Frequent or continuous risk | Evidence of >10 dengue cases in at least 3 of the previous 10 years | No | Included | CDC frequent or continuous risk classification |  |
| Belarus | BLR | Needs manual review | Not individually and unambiguously classified in the CDC text description | No | Excluded | Not included in the primary national-level endemic-country analysis |  |
| Belgium | BEL | Needs manual review | Not individually and unambiguously classified in the CDC text description | No | Excluded | Not included in the primary national-level endemic-country analysis |  |
| Belize | BLZ | Frequent or continuous risk | Evidence of >10 dengue cases in at least 3 of the previous 10 years | No | Included | CDC frequent or continuous risk classification |  |
| Benin | BEN | Sporadic or uncertain risk | Evidence of at least 1 locally acquired dengue case during the previous 10 years | No | Excluded | Not included in the primary national-level endemic-country analysis |  |
| Bermuda | BMU | Needs manual review | Not individually and unambiguously classified in the CDC text description | No | Excluded | Not included in the primary national-level endemic-country analysis |  |
| Bhutan | BTN | Frequent or continuous risk | Evidence of >10 dengue cases in at least 3 of the previous 10 years | No | Included | CDC frequent or continuous risk classification |  |
| Bolivia (Plurinational State of) | BOL | Frequent or continuous risk | Evidence of >10 dengue cases in at least 3 of the previous 10 years | No | Included | CDC frequent or continuous risk classification |  |
| Bosnia and Herzegovina | BIH | Needs manual review | Not individually and unambiguously classified in the CDC text description | No | Excluded | Not included in the primary national-level endemic-country analysis |  |
| Botswana | BWA | Needs manual review | Not individually and unambiguously classified in the CDC text description | No | Excluded | Not included in the primary national-level endemic-country analysis |  |
| Brazil | BRA | Frequent or continuous risk | Evidence of >10 dengue cases in at least 3 of the previous 10 years | No | Included | CDC frequent or continuous risk classification |  |
| Brunei Darussalam | BRN | Needs manual review | Not individually and unambiguously classified in the CDC text description | No | Included | Located within a frequent or continuous transmission area |  |
| Bulgaria | BGR | Needs manual review | Not individually and unambiguously classified in the CDC text description | No | Excluded | Not included in the primary national-level endemic-country analysis |  |
| Burkina Faso | BFA | Frequent or continuous risk | Evidence of >10 dengue cases in at least 3 of the previous 10 years | No | Included | CDC frequent or continuous risk classification |  |
| Burundi | BDI | Needs manual review | Not individually and unambiguously classified in the CDC text description | No | Excluded | Not included in the primary national-level endemic-country analysis |  |
| Cabo Verde | CPV | Sporadic or uncertain risk | Evidence of at least 1 locally acquired dengue case during the previous 10 years | No | Excluded | Not included in the primary national-level endemic-country analysis |  |
| Cambodia | KHM | Frequent or continuous risk | Evidence of >10 dengue cases in at least 3 of the previous 10 years | No | Included | CDC frequent or continuous risk classification |  |
| Cameroon | CMR | Frequent or continuous risk | Evidence of >10 dengue cases in at least 3 of the previous 10 years | No | Included | CDC frequent or continuous risk classification |  |
| Canada | CAN | No evidence of risk | No evidence of dengue risk shown in the CDC classification | No | Excluded | Not included in the primary national-level endemic-country analysis |  |
| Central African Republic | CAF | Sporadic or uncertain risk | Evidence of at least 1 locally acquired dengue case during the previous 10 years | No | Excluded | Not included in the primary national-level endemic-country analysis |  |
| Chad | TCD | Sporadic or uncertain risk | Evidence of at least 1 locally acquired dengue case during the previous 10 years | No | Excluded | Not included in the primary national-level endemic-country analysis |  |
| Chile | CHL | No evidence of risk | No evidence of dengue risk shown in the CDC classification | No | Excluded | Not included in the primary national-level endemic-country analysis |  |
| China | CHN | Needs manual review | Not individually and unambiguously classified in the CDC text description | Yes | Excluded | Frequent or local transmission restricted to a focal subnational area | CDC indicates frequent or continuous risk in parts of southeastern China and sporadic or uncertain risk in parts of southern China; national classification requires review. |
| Colombia | COL | Frequent or continuous risk | Evidence of >10 dengue cases in at least 3 of the previous 10 years | No | Included | CDC frequent or continuous risk classification |  |
| Comoros | COM | Sporadic or uncertain risk | Evidence of at least 1 locally acquired dengue case during the previous 10 years | No | Excluded | Not included in the primary national-level endemic-country analysis |  |
| Congo | COG | Needs manual review | Not individually and unambiguously classified in the CDC text description | No | Excluded | Not included in the primary national-level endemic-country analysis |  |
| Cook Islands | COK | Frequent or continuous risk | Evidence of >10 dengue cases in at least 3 of the previous 10 years | No | Included | CDC frequent or continuous risk classification |  |
| Costa Rica | CRI | Frequent or continuous risk | Evidence of >10 dengue cases in at least 3 of the previous 10 years | No | Included | CDC frequent or continuous risk classification |  |
| Croatia | HRV | Needs manual review | Not individually and unambiguously classified in the CDC text description | No | Excluded | Not included in the primary national-level endemic-country analysis |  |
| Cuba | CUB | Frequent or continuous risk | Evidence of >10 dengue cases in at least 3 of the previous 10 years | No | Included | CDC frequent or continuous risk classification |  |
| Cyprus | CYP | Needs manual review | Not individually and unambiguously classified in the CDC text description | No | Excluded | Not included in the primary national-level endemic-country analysis |  |
| Czechia | CZE | Needs manual review | Not individually and unambiguously classified in the CDC text description | No | Excluded | Not included in the primary national-level endemic-country analysis |  |
| C么te d'Ivoire | CIV | Frequent or continuous risk | Evidence of >10 dengue cases in at least 3 of the previous 10 years | No | Included | CDC frequent or continuous risk classification |  |
| Democratic People's Republic of Korea | PRK | Needs manual review | Not individually and unambiguously classified in the CDC text description | No | Excluded | Not included in the primary national-level endemic-country analysis |  |
| Democratic Republic of the Congo | COD | Sporadic or uncertain risk | Evidence of at least 1 locally acquired dengue case during the previous 10 years | No | Excluded | Not included in the primary national-level endemic-country analysis |  |
| Denmark | DNK | Needs manual review | Not individually and unambiguously classified in the CDC text description | No | Excluded | Not included in the primary national-level endemic-country analysis |  |
| Djibouti | DJI | Sporadic or uncertain risk | Evidence of at least 1 locally acquired dengue case during the previous 10 years | No | Excluded | Not included in the primary national-level endemic-country analysis |  |
| Dominica | DMA | Frequent or continuous risk | Evidence of >10 dengue cases in at least 3 of the previous 10 years | No | Included | CDC frequent or continuous risk classification |  |
| Dominican Republic | DOM | Frequent or continuous risk | Evidence of >10 dengue cases in at least 3 of the previous 10 years | No | Included | CDC frequent or continuous risk classification |  |
| Ecuador | ECU | Frequent or continuous risk | Evidence of >10 dengue cases in at least 3 of the previous 10 years | No | Included | CDC frequent or continuous risk classification |  |
| Egypt | EGY | Frequent or continuous risk | Evidence of >10 dengue cases in at least 3 of the previous 10 years | No | Included | CDC frequent or continuous risk classification |  |
| El Salvador | SLV | Frequent or continuous risk | Evidence of >10 dengue cases in at least 3 of the previous 10 years | No | Included | CDC frequent or continuous risk classification |  |
| Equatorial Guinea | GNQ | Sporadic or uncertain risk | Evidence of at least 1 locally acquired dengue case during the previous 10 years | No | Excluded | Not included in the primary national-level endemic-country analysis |  |
| Eritrea | ERI | Frequent or continuous risk | Evidence of >10 dengue cases in at least 3 of the previous 10 years | No | Included | CDC frequent or continuous risk classification |  |
| Estonia | EST | Needs manual review | Not individually and unambiguously classified in the CDC text description | No | Excluded | Not included in the primary national-level endemic-country analysis |  |
| Eswatini | SWZ | Needs manual review | Not individually and unambiguously classified in the CDC text description | No | Excluded | Not included in the primary national-level endemic-country analysis |  |
| Ethiopia | ETH | Frequent or continuous risk | Evidence of >10 dengue cases in at least 3 of the previous 10 years | No | Included | CDC frequent or continuous risk classification |  |
| Fiji | FJI | Frequent or continuous risk | Evidence of >10 dengue cases in at least 3 of the previous 10 years | No | Included | CDC frequent or continuous risk classification |  |
| Finland | FIN | Needs manual review | Not individually and unambiguously classified in the CDC text description | No | Excluded | Not included in the primary national-level endemic-country analysis |  |
| France | FRA | Sporadic or uncertain risk | Evidence of at least 1 locally acquired dengue case during the previous 10 years | No | Excluded | Not included in the primary national-level endemic-country analysis |  |
| Gabon | GAB | Frequent or continuous risk | Evidence of >10 dengue cases in at least 3 of the previous 10 years | No | Included | CDC frequent or continuous risk classification |  |
| Gambia | GMB | Needs manual review | Not individually and unambiguously classified in the CDC text description | No | Excluded | Not included in the primary national-level endemic-country analysis |  |
| Georgia | GEO | Needs manual review | Not individually and unambiguously classified in the CDC text description | No | Excluded | Not included in the primary national-level endemic-country analysis |  |
| Germany | DEU | Needs manual review | Not individually and unambiguously classified in the CDC text description | No | Excluded | Not included in the primary national-level endemic-country analysis |  |
| Ghana | GHA | Sporadic or uncertain risk | Evidence of at least 1 locally acquired dengue case during the previous 10 years | No | Excluded | Not included in the primary national-level endemic-country analysis |  |
| Greece | GRC | Needs manual review | Not individually and unambiguously classified in the CDC text description | No | Excluded | Not included in the primary national-level endemic-country analysis |  |
| Greenland | GRL | Needs manual review | Not individually and unambiguously classified in the CDC text description | No | Excluded | Not included in the primary national-level endemic-country analysis |  |
| Grenada | GRD | Frequent or continuous risk | Evidence of >10 dengue cases in at least 3 of the previous 10 years | No | Included | CDC frequent or continuous risk classification |  |
| Guam | GUM | Sporadic or uncertain risk | Evidence of at least 1 locally acquired dengue case during the previous 10 years | No | Excluded | Not included in the primary national-level endemic-country analysis |  |
| Guatemala | GTM | Frequent or continuous risk | Evidence of >10 dengue cases in at least 3 of the previous 10 years | No | Included | CDC frequent or continuous risk classification |  |
| Guinea | GIN | Sporadic or uncertain risk | Evidence of at least 1 locally acquired dengue case during the previous 10 years | No | Excluded | Not included in the primary national-level endemic-country analysis |  |
| Guinea-Bissau | GNB | Needs manual review | Not individually and unambiguously classified in the CDC text description | No | Excluded | Not included in the primary national-level endemic-country analysis |  |
| Guyana | GUY | Frequent or continuous risk | Evidence of >10 dengue cases in at least 3 of the previous 10 years | No | Included | CDC frequent or continuous risk classification |  |
| Haiti | HTI | Frequent or continuous risk | Evidence of >10 dengue cases in at least 3 of the previous 10 years | No | Included | CDC frequent or continuous risk classification |  |
| Honduras | HND | Frequent or continuous risk | Evidence of >10 dengue cases in at least 3 of the previous 10 years | No | Included | CDC frequent or continuous risk classification |  |
| Hungary | HUN | Needs manual review | Not individually and unambiguously classified in the CDC text description | No | Excluded | Not included in the primary national-level endemic-country analysis |  |
| Iceland | ISL | Needs manual review | Not individually and unambiguously classified in the CDC text description | No | Excluded | Not included in the primary national-level endemic-country analysis |  |
| India | IND | Frequent or continuous risk | Evidence of >10 dengue cases in at least 3 of the previous 10 years | No | Included | CDC frequent or continuous risk classification |  |
| Indonesia | IDN | Frequent or continuous risk | Evidence of >10 dengue cases in at least 3 of the previous 10 years | No | Included | CDC frequent or continuous risk classification |  |
| Iran (Islamic Republic of) | IRN | Needs manual review | Not individually and unambiguously classified in the CDC text description | No | Excluded | Not included in the primary national-level endemic-country analysis |  |
| Iraq | IRQ | Needs manual review | Not individually and unambiguously classified in the CDC text description | No | Excluded | Not included in the primary national-level endemic-country analysis |  |
| Ireland | IRL | Needs manual review | Not individually and unambiguously classified in the CDC text description | No | Excluded | Not included in the primary national-level endemic-country analysis |  |
| Israel | ISR | Needs manual review | Not individually and unambiguously classified in the CDC text description | No | Excluded | Not included in the primary national-level endemic-country analysis |  |
| Italy | ITA | Sporadic or uncertain risk | Evidence of at least 1 locally acquired dengue case during the previous 10 years | No | Excluded | Not included in the primary national-level endemic-country analysis |  |
| Jamaica | JAM | Frequent or continuous risk | Evidence of >10 dengue cases in at least 3 of the previous 10 years | No | Included | CDC frequent or continuous risk classification |  |
| Japan | JPN | Sporadic or uncertain risk | Evidence of at least 1 locally acquired dengue case during the previous 10 years | No | Excluded | Not included in the primary national-level endemic-country analysis |  |
| Jordan | JOR | Needs manual review | Not individually and unambiguously classified in the CDC text description | No | Excluded | Not included in the primary national-level endemic-country analysis |  |
| Kazakhstan | KAZ | Needs manual review | Not individually and unambiguously classified in the CDC text description | No | Excluded | Not included in the primary national-level endemic-country analysis |  |
| Kenya | KEN | Frequent or continuous risk | Evidence of >10 dengue cases in at least 3 of the previous 10 years | No | Included | CDC frequent or continuous risk classification |  |
| Kiribati | KIR | Sporadic or uncertain risk | Evidence of at least 1 locally acquired dengue case during the previous 10 years | No | Excluded | Not included in the primary national-level endemic-country analysis |  |
| Kuwait | KWT | Needs manual review | Not individually and unambiguously classified in the CDC text description | No | Excluded | Not included in the primary national-level endemic-country analysis |  |
| Kyrgyzstan | KGZ | Needs manual review | Not individually and unambiguously classified in the CDC text description | No | Excluded | Not included in the primary national-level endemic-country analysis |  |
| Lao People's Democratic Republic | LAO | Frequent or continuous risk | Evidence of >10 dengue cases in at least 3 of the previous 10 years | No | Included | CDC frequent or continuous risk classification |  |
| Latvia | LVA | Needs manual review | Not individually and unambiguously classified in the CDC text description | No | Excluded | Not included in the primary national-level endemic-country analysis |  |
| Lebanon | LBN | Needs manual review | Not individually and unambiguously classified in the CDC text description | No | Excluded | Not included in the primary national-level endemic-country analysis |  |
| Lesotho | LSO | Needs manual review | Not individually and unambiguously classified in the CDC text description | No | Excluded | Not included in the primary national-level endemic-country analysis |  |
| Liberia | LBR | Sporadic or uncertain risk | Evidence of at least 1 locally acquired dengue case during the previous 10 years | No | Excluded | Not included in the primary national-level endemic-country analysis |  |
| Libya | LBY | Needs manual review | Not individually and unambiguously classified in the CDC text description | No | Excluded | Not included in the primary national-level endemic-country analysis |  |
| Lithuania | LTU | Needs manual review | Not individually and unambiguously classified in the CDC text description | No | Excluded | Not included in the primary national-level endemic-country analysis |  |
| Luxembourg | LUX | Needs manual review | Not individually and unambiguously classified in the CDC text description | No | Excluded | Not included in the primary national-level endemic-country analysis |  |
| Madagascar | MDG | Sporadic or uncertain risk | Evidence of at least 1 locally acquired dengue case during the previous 10 years | No | Excluded | Not included in the primary national-level endemic-country analysis |  |
| Malawi | MWI | Needs manual review | Not individually and unambiguously classified in the CDC text description | No | Excluded | Not included in the primary national-level endemic-country analysis |  |
| Malaysia | MYS | Frequent or continuous risk | Evidence of >10 dengue cases in at least 3 of the previous 10 years | No | Included | CDC frequent or continuous risk classification |  |
| Maldives | MDV | Frequent or continuous risk | Evidence of >10 dengue cases in at least 3 of the previous 10 years | No | Included | CDC frequent or continuous risk classification |  |
| Mali | MLI | Sporadic or uncertain risk | Evidence of at least 1 locally acquired dengue case during the previous 10 years | No | Excluded | Not included in the primary national-level endemic-country analysis |  |
| Malta | MLT | Needs manual review | Not individually and unambiguously classified in the CDC text description | No | Excluded | Not included in the primary national-level endemic-country analysis |  |
| Marshall Islands | MHL | Sporadic or uncertain risk | Evidence of at least 1 locally acquired dengue case during the previous 10 years | No | Excluded | Not included in the primary national-level endemic-country analysis |  |
| Mauritania | MRT | Sporadic or uncertain risk | Evidence of at least 1 locally acquired dengue case during the previous 10 years | No | Excluded | Not included in the primary national-level endemic-country analysis |  |
| Mauritius | MUS | Frequent or continuous risk | Evidence of >10 dengue cases in at least 3 of the previous 10 years | No | Included | CDC frequent or continuous risk classification |  |
| Mexico | MEX | Frequent or continuous risk | Evidence of >10 dengue cases in at least 3 of the previous 10 years | No | Included | CDC frequent or continuous risk classification |  |
| Micronesia (Federated States of) | FSM | Frequent or continuous risk | Evidence of >10 dengue cases in at least 3 of the previous 10 years | No | Included | CDC frequent or continuous risk classification |  |
| Monaco | MCO | Needs manual review | Not individually and unambiguously classified in the CDC text description | No | Excluded | Not included in the primary national-level endemic-country analysis |  |
| Mongolia | MNG | Needs manual review | Not individually and unambiguously classified in the CDC text description | No | Excluded | Not included in the primary national-level endemic-country analysis |  |
| Montenegro | MNE | Needs manual review | Not individually and unambiguously classified in the CDC text description | No | Excluded | Not included in the primary national-level endemic-country analysis |  |
| Morocco | MAR | Needs manual review | Not individually and unambiguously classified in the CDC text description | No | Excluded | Not included in the primary national-level endemic-country analysis |  |
| Mozambique | MOZ | Sporadic or uncertain risk | Evidence of at least 1 locally acquired dengue case during the previous 10 years | No | Excluded | Not included in the primary national-level endemic-country analysis |  |
| Myanmar | MMR | Frequent or continuous risk | Evidence of >10 dengue cases in at least 3 of the previous 10 years | No | Included | CDC frequent or continuous risk classification |  |
| Namibia | NAM | Needs manual review | Not individually and unambiguously classified in the CDC text description | No | Excluded | Not included in the primary national-level endemic-country analysis |  |
| Nauru | NRU | Frequent or continuous risk | Evidence of >10 dengue cases in at least 3 of the previous 10 years | No | Included | CDC frequent or continuous risk classification |  |
| Nepal | NPL | Frequent or continuous risk | Evidence of >10 dengue cases in at least 3 of the previous 10 years | No | Included | CDC frequent or continuous risk classification |  |
| Netherlands | NLD | Needs manual review | Not individually and unambiguously classified in the CDC text description | No | Excluded | Not included in the primary national-level endemic-country analysis |  |
| New Zealand | NZL | Needs manual review | Not individually and unambiguously classified in the CDC text description | No | Excluded | Not included in the primary national-level endemic-country analysis |  |
| Nicaragua | NIC | Frequent or continuous risk | Evidence of >10 dengue cases in at least 3 of the previous 10 years | No | Included | CDC frequent or continuous risk classification |  |
| Niger | NER | Needs manual review | Not individually and unambiguously classified in the CDC text description | No | Excluded | Not included in the primary national-level endemic-country analysis |  |
| Nigeria | NGA | Sporadic or uncertain risk | Evidence of at least 1 locally acquired dengue case during the previous 10 years | No | Excluded | Not included in the primary national-level endemic-country analysis |  |
| Niue | NIU | Sporadic or uncertain risk | Evidence of at least 1 locally acquired dengue case during the previous 10 years | No | Excluded | Not included in the primary national-level endemic-country analysis |  |
| North Macedonia | MKD | Needs manual review | Not individually and unambiguously classified in the CDC text description | No | Excluded | Not included in the primary national-level endemic-country analysis |  |
| Northern Mariana Islands | MNP | Needs manual review | Not individually and unambiguously classified in the CDC text description | No | Excluded | Not included in the primary national-level endemic-country analysis |  |
| Norway | NOR | Needs manual review | Not individually and unambiguously classified in the CDC text description | No | Excluded | Not included in the primary national-level endemic-country analysis |  |
| Oman | OMN | Sporadic or uncertain risk | Evidence of at least 1 locally acquired dengue case during the previous 10 years | No | Excluded | Not included in the primary national-level endemic-country analysis |  |
| Pakistan | PAK | Frequent or continuous risk | Evidence of >10 dengue cases in at least 3 of the previous 10 years | No | Included | CDC frequent or continuous risk classification |  |
| Palau | PLW | Needs manual review | Not individually and unambiguously classified in the CDC text description | No | Included | Established recurrent dengue transmission in the Pacific |  |
| Palestine | PSE | Needs manual review | Not individually and unambiguously classified in the CDC text description | No | Excluded | Not included in the primary national-level endemic-country analysis |  |
| Panama | PAN | Frequent or continuous risk | Evidence of >10 dengue cases in at least 3 of the previous 10 years | No | Included | CDC frequent or continuous risk classification |  |
| Papua New Guinea | PNG | Frequent or continuous risk | Evidence of >10 dengue cases in at least 3 of the previous 10 years | No | Included | CDC frequent or continuous risk classification |  |
| Paraguay | PRY | Frequent or continuous risk | Evidence of >10 dengue cases in at least 3 of the previous 10 years | No | Included | CDC frequent or continuous risk classification |  |
| Peru | PER | Frequent or continuous risk | Evidence of >10 dengue cases in at least 3 of the previous 10 years | No | Included | CDC frequent or continuous risk classification |  |
| Philippines | PHL | Frequent or continuous risk | Evidence of >10 dengue cases in at least 3 of the previous 10 years | No | Included | CDC frequent or continuous risk classification |  |
| Poland | POL | Needs manual review | Not individually and unambiguously classified in the CDC text description | No | Excluded | Not included in the primary national-level endemic-country analysis |  |
| Portugal | PRT | Needs manual review | Not individually and unambiguously classified in the CDC text description | No | Excluded | Not included in the primary national-level endemic-country analysis |  |
| Puerto Rico | PRI | Frequent or continuous risk | Evidence of >10 dengue cases in at least 3 of the previous 10 years | No | Included | CDC frequent or continuous risk classification |  |
| Qatar | QAT | Needs manual review | Not individually and unambiguously classified in the CDC text description | No | Excluded | Not included in the primary national-level endemic-country analysis |  |
| Republic of Korea | KOR | Needs manual review | Not individually and unambiguously classified in the CDC text description | No | Excluded | Not included in the primary national-level endemic-country analysis |  |
| Republic of Moldova | MDA | Needs manual review | Not individually and unambiguously classified in the CDC text description | No | Excluded | Not included in the primary national-level endemic-country analysis |  |
| Romania | ROU | Needs manual review | Not individually and unambiguously classified in the CDC text description | No | Excluded | Not included in the primary national-level endemic-country analysis |  |
| Russian Federation | RUS | Needs manual review | Not individually and unambiguously classified in the CDC text description | No | Excluded | Not included in the primary national-level endemic-country analysis |  |
| Rwanda | RWA | Sporadic or uncertain risk | Evidence of at least 1 locally acquired dengue case during the previous 10 years | No | Excluded | Not included in the primary national-level endemic-country analysis |  |
| Saint Kitts and Nevis | KNA | Frequent or continuous risk | Evidence of >10 dengue cases in at least 3 of the previous 10 years | No | Included | CDC frequent or continuous risk classification |  |
| Saint Lucia | LCA | Frequent or continuous risk | Evidence of >10 dengue cases in at least 3 of the previous 10 years | No | Included | CDC frequent or continuous risk classification |  |
| Saint Vincent and the Grenadines | VCT | Frequent or continuous risk | Evidence of >10 dengue cases in at least 3 of the previous 10 years | No | Included | CDC frequent or continuous risk classification |  |
| Samoa | WSM | Frequent or continuous risk | Evidence of >10 dengue cases in at least 3 of the previous 10 years | No | Included | CDC frequent or continuous risk classification |  |
| San Marino | SMR | Needs manual review | Not individually and unambiguously classified in the CDC text description | No | Excluded | Not included in the primary national-level endemic-country analysis |  |
| Sao Tome and Principe | STP | Sporadic or uncertain risk | Evidence of at least 1 locally acquired dengue case during the previous 10 years | No | Excluded | Not included in the primary national-level endemic-country analysis |  |
| Saudi Arabia | SAU | Frequent or continuous risk | Evidence of >10 dengue cases in at least 3 of the previous 10 years | No | Included | CDC frequent or continuous risk classification |  |
| Senegal | SEN | Frequent or continuous risk | Evidence of >10 dengue cases in at least 3 of the previous 10 years | No | Included | CDC frequent or continuous risk classification |  |
| Serbia | SRB | Needs manual review | Not individually and unambiguously classified in the CDC text description | No | Excluded | Not included in the primary national-level endemic-country analysis |  |
| Seychelles | SYC | Sporadic or uncertain risk | Evidence of at least 1 locally acquired dengue case during the previous 10 years | No | Excluded | Not included in the primary national-level endemic-country analysis |  |
| Sierra Leone | SLE | Sporadic or uncertain risk | Evidence of at least 1 locally acquired dengue case during the previous 10 years | No | Excluded | Not included in the primary national-level endemic-country analysis |  |
| Singapore | SGP | Frequent or continuous risk | Evidence of >10 dengue cases in at least 3 of the previous 10 years | No | Included | CDC frequent or continuous risk classification |  |
| Slovakia | SVK | Needs manual review | Not individually and unambiguously classified in the CDC text description | No | Excluded | Not included in the primary national-level endemic-country analysis |  |
| Slovenia | SVN | Needs manual review | Not individually and unambiguously classified in the CDC text description | No | Excluded | Not included in the primary national-level endemic-country analysis |  |
| Solomon Islands | SLB | Frequent or continuous risk | Evidence of >10 dengue cases in at least 3 of the previous 10 years | No | Included | CDC frequent or continuous risk classification |  |
| Somalia | SOM | Frequent or continuous risk | Evidence of >10 dengue cases in at least 3 of the previous 10 years | No | Included | CDC frequent or continuous risk classification |  |
| South Africa | ZAF | Needs manual review | Not individually and unambiguously classified in the CDC text description | No | Excluded | Not included in the primary national-level endemic-country analysis |  |
| South Sudan | SSD | Needs manual review | Not individually and unambiguously classified in the CDC text description | No | Excluded | Not included in the primary national-level endemic-country analysis |  |
| Spain | ESP | Sporadic or uncertain risk | Evidence of at least 1 locally acquired dengue case during the previous 10 years | No | Excluded | Not included in the primary national-level endemic-country analysis |  |
| Sri Lanka | LKA | Frequent or continuous risk | Evidence of >10 dengue cases in at least 3 of the previous 10 years | No | Included | CDC frequent or continuous risk classification |  |
| Sudan | SDN | Frequent or continuous risk | Evidence of >10 dengue cases in at least 3 of the previous 10 years | No | Included | CDC frequent or continuous risk classification |  |
| Suriname | SUR | Frequent or continuous risk | Evidence of >10 dengue cases in at least 3 of the previous 10 years | No | Included | CDC frequent or continuous risk classification |  |
| Sweden | SWE | Needs manual review | Not individually and unambiguously classified in the CDC text description | No | Excluded | Not included in the primary national-level endemic-country analysis |  |
| Switzerland | CHE | Needs manual review | Not individually and unambiguously classified in the CDC text description | No | Excluded | Not included in the primary national-level endemic-country analysis |  |
| Syrian Arab Republic | SYR | Needs manual review | Not individually and unambiguously classified in the CDC text description | No | Excluded | Not included in the primary national-level endemic-country analysis |  |
| Taiwan | TWN | Frequent or continuous risk | Evidence of >10 dengue cases in at least 3 of the previous 10 years | No | Included | CDC frequent or continuous risk classification |  |
| Tajikistan | TJK | Needs manual review | Not individually and unambiguously classified in the CDC text description | No | Excluded | Not included in the primary national-level endemic-country analysis |  |
| Thailand | THA | Frequent or continuous risk | Evidence of >10 dengue cases in at least 3 of the previous 10 years | No | Included | CDC frequent or continuous risk classification |  |
| Timor-Leste | TLS | Frequent or continuous risk | Evidence of >10 dengue cases in at least 3 of the previous 10 years | No | Included | CDC frequent or continuous risk classification |  |
| Togo | TGO | Sporadic or uncertain risk | Evidence of at least 1 locally acquired dengue case during the previous 10 years | No | Excluded | Not included in the primary national-level endemic-country analysis |  |
| Tokelau | TKL | Sporadic or uncertain risk | Evidence of at least 1 locally acquired dengue case during the previous 10 years | No | Excluded | Not included in the primary national-level endemic-country analysis |  |
| Tonga | TON | Frequent or continuous risk | Evidence of >10 dengue cases in at least 3 of the previous 10 years | No | Included | CDC frequent or continuous risk classification |  |
| Trinidad and Tobago | TTO | Frequent or continuous risk | Evidence of >10 dengue cases in at least 3 of the previous 10 years | No | Included | CDC frequent or continuous risk classification |  |
| Tunisia | TUN | Needs manual review | Not individually and unambiguously classified in the CDC text description | No | Excluded | Not included in the primary national-level endemic-country analysis |  |
| Turkmenistan | TKM | Needs manual review | Not individually and unambiguously classified in the CDC text description | No | Excluded | Not included in the primary national-level endemic-country analysis |  |
| Tuvalu | TUV | Frequent or continuous risk | Evidence of >10 dengue cases in at least 3 of the previous 10 years | No | Included | CDC frequent or continuous risk classification |  |
| Türkiye | TUR | Needs manual review | Not individually and unambiguously classified in the CDC text description | No | Excluded | Not included in the primary national-level endemic-country analysis |  |
| Uganda | UGA | Sporadic or uncertain risk | Evidence of at least 1 locally acquired dengue case during the previous 10 years | No | Excluded | Not included in the primary national-level endemic-country analysis |  |
| Ukraine | UKR | Needs manual review | Not individually and unambiguously classified in the CDC text description | No | Excluded | Not included in the primary national-level endemic-country analysis |  |
| United Arab Emirates | ARE | Needs manual review | Not individually and unambiguously classified in the CDC text description | No | Excluded | Not included in the primary national-level endemic-country analysis |  |
| United Kingdom | GBR | No evidence of risk | No evidence of dengue risk shown in the CDC classification | No | Excluded | Not included in the primary national-level endemic-country analysis |  |
| United Republic of Tanzania | TZA | Frequent or continuous risk | Evidence of >10 dengue cases in at least 3 of the previous 10 years | No | Included | CDC frequent or continuous risk classification |  |
| United States Virgin Islands | VIR | Frequent or continuous risk | Evidence of >10 dengue cases in at least 3 of the previous 10 years | No | Included | CDC frequent or continuous risk classification |  |
| United States of America | USA | Sporadic or uncertain risk | Evidence of at least 1 locally acquired dengue case during the previous 10 years | No | Excluded | Frequent or local transmission restricted to a focal subnational area | Local transmission is focal and subnational; excluded from the primary endemic-country analysis. |
| Uruguay | URY | Sporadic or uncertain risk | Evidence of at least 1 locally acquired dengue case during the previous 10 years | No | Excluded | Not included in the primary national-level endemic-country analysis |  |
| Uzbekistan | UZB | Needs manual review | Not individually and unambiguously classified in the CDC text description | No | Excluded | Not included in the primary national-level endemic-country analysis |  |
| Vanuatu | VUT | Frequent or continuous risk | Evidence of >10 dengue cases in at least 3 of the previous 10 years | No | Included | CDC frequent or continuous risk classification |  |
| Venezuela (Bolivarian Republic of) | VEN | Frequent or continuous risk | Evidence of >10 dengue cases in at least 3 of the previous 10 years | No | Included | CDC frequent or continuous risk classification |  |
| Viet Nam | VNM | Frequent or continuous risk | Evidence of >10 dengue cases in at least 3 of the previous 10 years | No | Included | CDC frequent or continuous risk classification |  |
| Yemen | YEM | Frequent or continuous risk | Evidence of >10 dengue cases in at least 3 of the previous 10 years | No | Included | CDC frequent or continuous risk classification |  |
| Zambia | ZMB | Needs manual review | Not individually and unambiguously classified in the CDC text description | No | Excluded | Not included in the primary national-level endemic-country analysis |  |
| Zimbabwe | ZWE | Needs manual review | Not individually and unambiguously classified in the CDC text description | No | Excluded | Not included in the primary national-level endemic-country analysis |  |

Note: Dengue risk classifications were derived from the global dengue risk classification presented in the CDC Yellow Book 2026, Map 4.4.1. Frequent or continuous risk was defined as evidence of more than 10 dengue cases in at least 3 of the preceding 10 years, whereas sporadic or uncertain risk was defined as evidence of at least one locally acquired dengue case during the preceding 10 years. Because dengue transmission may vary within countries, countries with frequent or continuous transmission restricted to a small focal subnational area were excluded from the primary country-level analysis. Source: CDC Yellow Book 2026, “Dengue.” Available at: https://www.cdc.gov/yellow-book/hcp/travel-associated-infections-diseases/dengue.html (accessed 20 September 2026).

| **Supplementary Table S2.** Country-level publication output, mean Index-P, and age-standardized dengue DALY rates with 95% uncertainty intervals among 80 dengue-endemic countries and territories. | | | | | | |
| --- | --- | --- | --- | --- | --- | --- |
| Country | ISO3 code | DALY rate, 2023 | Lower 95% UI | Upper 95% UI | Publication count | Mean Index-P |
| Afghanistan | AFG | 0.048 | 0.023 | 0.083 | 0 | 0.1784 |
| American Samoa | ASM | 20.378 | 10.117 | 36.374 | 0 |  |
| Antigua and Barbuda | ATG | 0.614 | 0.349 | 1.012 | 0 |  |
| Argentina | ARG | 12.875 | 6.168 | 23.513 | 9 | 0.2149 |
| Bahamas | BHS | 3.185 | 1.528 | 5.375 | 0 | 1.4887 |
| Bangladesh | BGD | 27.795 | 16.921 | 43.461 | 3 | 1.5809 |
| Barbados | BRB | 16.487 | 8.684 | 26.593 | 4 |  |
| Belize | BLZ | 28.458 | 13.601 | 48.947 | 0 | 1.0901 |
| Bhutan | BTN | 29.342 | 8.525 | 78.207 | 0 | 0.1049 |
| Bolivia (Plurinational State of) | BOL | 67.524 | 31.303 | 119.398 | 0 | 0.749 |
| Brazil | BRA | 93.429 | 51.063 | 157.853 | 25 | 1.1047 |
| Brunei Darussalam | BRN | 2.629 | 1.112 | 5.946 | 0 | 1.2526 |
| Burkina Faso | BFA | 4.38 | 2.135 | 8.925 | 0 | 1.8441 |
| Cambodia | KHM | 19.097 | 6.88 | 46.501 | 1 | 1.7096 |
| Cameroon | CMR | 34.344 | 6.284 | 128.095 | 0 | 0.9319 |
| Colombia | COL | 12.042 | 7.222 | 19.276 | 7 | 0.918 |
| Cook Islands | COK | 0.287 | 0.096 | 0.748 | 0 |  |
| Costa Rica | CRI | 33.851 | 15.822 | 61.63 | 3 | 0.7284 |
| Cuba | CUB | 0.288 | 0.134 | 0.511 | 1 | 1.363 |
| Côte d’Ivoire | CIV | 27.893 | 5.324 | 95.71 | 0 | 1.4106 |
| Dominica | DMA | 0.401 | 0.199 | 0.694 | 0 |  |
| Dominican Republic | DOM | 7.204 | 4.66 | 12.559 | 0 | 0.8958 |
| Ecuador | ECU | 8.394 | 3.948 | 15.148 | 9 | 0.5057 |
| Egypt | EGY | 0.023 | 0.011 | 0.046 | 0 | 0.5817 |
| El Salvador | SLV | 8.294 | 5.48 | 13.191 | 0 | 1.2853 |
| Eritrea | ERI | 7.983 | 1.296 | 29.886 | 0 | 1.0761 |
| Ethiopia | ETH | 7.984 | 1.721 | 24.791 | 3 | 0.7817 |
| Fiji | FJI | 10.219 | 2.923 | 23.469 | 2 |  |
| Gabon | GAB | 1.262 | 0.444 | 3.459 | 0 | 0.9386 |
| Grenada | GRD | 0.949 | 0.44 | 1.688 | 0 |  |
| Guatemala | GTM | 56.035 | 33.829 | 87.284 | 0 | 0.6924 |
| Guyana | GUY | 192.624 | 95.298 | 342.142 | 1 | 1.0697 |
| Haiti | HTI | 0.647 | 0.296 | 1.437 | 0 | 1.0515 |
| Honduras | HND | 35.462 | 16.215 | 67.552 | 1 | 0.6649 |
| India | IND | 92.754 | 48.783 | 196.373 | 5 | 1.1748 |
| Indonesia | IDN | 364.851 | 130.878 | 825.134 | 6 | 1.0717 |
| Jamaica | JAM | 17.174 | 8.918 | 28.781 | 1 | 1.1064 |
| Kenya | KEN | 1.531 | 0.551 | 4.082 | 3 | 1.1933 |
| Lao People's Democratic Republic | LAO | 14.501 | 5.08 | 37.905 | 3 | 0.7699 |
| Malaysia | MYS | 46.849 | 31.863 | 71.109 | 10 | 1.0801 |
| Maldives | MDV | 54.703 | 29.26 | 93.481 | 0 |  |
| Mauritius | MUS | 1.705 | 0.786 | 3.117 | 2 | 0.9391 |
| Mexico | MEX | 13.843 | 6.642 | 24.867 | 8 | 0.586 |
| Micronesia (Federated States of) | FSM | 2.256 | 1.238 | 4.031 | 0 |  |
| Myanmar | MMR | 62.292 | 18.197 | 146.32 | 0 | 0.9472 |
| Nauru | NRU | 3.15 | 1.395 | 5.562 | 0 |  |
| Nepal | NPL | 81.513 | 29.199 | 198.643 | 0 | 0.3944 |
| Nicaragua | NIC | 474.552 | 256.011 | 815.023 | 0 | 1.1268 |
| Pakistan | PAK | 29.697 | 9.22 | 78.548 | 8 | 0.6668 |
| Palau | PLW | 4.875 | 1.123 | 17.116 | 0 |  |
| Panama | PAN | 22.015 | 10.465 | 39.319 | 2 | 0.9907 |
| Papua New Guinea | PNG | 0.552 | 0.185 | 1.301 | 0 | 0.8421 |
| Paraguay | PRY | 43.363 | 20.932 | 74.739 | 0 | 0.9526 |
| Peru | PER | 74.685 | 52.22 | 107.449 | 6 | 0.5205 |
| Philippines | PHL | 37.924 | 25.412 | 54.059 | 5 | 1.166 |
| Puerto Rico | PRI | 2.471 | 1.508 | 3.812 | 0 | 1.092 |
| Saint Kitts and Nevis | KNA | 5.927 | 2.833 | 10.854 | 0 |  |
| Saint Lucia | LCA | 1.568 | 0.927 | 2.471 | 0 |  |
| Saint Vincent and the Grenadines | VCT | 0.708 | 0.363 | 1.208 | 0 |  |
| Samoa | WSM | 0.809 | 0.391 | 1.527 | 0 | 0.6036 |
| Saudi Arabia | SAU | 0.856 | 0.414 | 1.628 | 2 | 0.4977 |
| Senegal | SEN | 22.752 | 3.604 | 82.762 | 1 | 1.971 |
| Singapore | SGP | 3.848 | 1.908 | 6.606 | 17 | 1.5966 |
| Solomon Islands | SLB | 0.533 | 0.197 | 1.263 | 0 | 1.2923 |
| Somalia | SOM | 6.27 | 1.19 | 21.586 | 0 | 1.451 |
| Sri Lanka | LKA | 42.115 | 25.226 | 66.719 | 4 | 1.449 |
| Sudan | SDN | 0.209 | 0.095 | 0.373 | 0 | 1.1359 |
| Suriname | SUR | 14.35 | 8.022 | 28.524 | 0 | 1.2329 |
| Taiwan | TWN | 1.257 | 0.44 | 2.519 | 0 | 0.4714 |
| Thailand | THA | 35.469 | 20.774 | 59.448 | 9 | 1.4315 |
| Timor-Leste | TLS | 24.973 | 8.632 | 65.461 | 0 | 0.9963 |
| Tonga | TON | 30.098 | 9.976 | 78.475 | 0 |  |
| Trinidad and Tobago | TTO | 19.256 | 5.512 | 43.551 | 0 | 1.4749 |
| Tuvalu | TUV | 3.509 | 1.729 | 5.934 | 0 |  |
| United Republic of Tanzania | TZA | 6.86 | 1.256 | 22.309 | 1 | 0.6273 |
| United States Virgin Islands | VIR | 0.351 | 0.166 | 0.627 | 0 |  |
| Vanuatu | VUT | 2.13 | 1.078 | 3.715 | 0 | 1.0948 |
| Venezuela (Bolivarian Republic of) | VEN | 8.24 | 5.555 | 12.852 | 4 | 1.1576 |
| Viet Nam | VNM | 14.356 | 5.779 | 33.723 | 11 | 0.9483 |
| Yemen | YEM | 5.371 | 2.429 | 9.791 | 0 | 0.8234 |

| **Supplementary Table S3.** Sensitivity analyses of the association between country-level publication output and alternative estimates of the 2023 age-standardized dengue DALY rate among 80 dengue-endemic countries and territories. | | | |
| --- | --- | --- | --- |
| DALY measure | Number of countries | Spearman's rho | P value |
| DALY rate point estimate | 80 | 0.36 | <0.001 |
| Lower bound of DALY rate 95% UI | 80 | 0.4 | <0.001 |
| Upper bound of DALY rate 95% UI | 80 | 0.31 | 0.005 |

| **Supplementary Table S4.** Bibliographic information and reasons for exclusion of records during title and abstract screening | | | | | | | |
| --- | --- | --- | --- | --- | --- | --- | --- |
| **No.** | **First author** | **Year** | **Title** | **Journal** | **DOI** | **Primary exclusion reason** | **Brief record-specific justification** |
| 1 | Lim | 2021 | Mosquito abundance in relation to extremely high temperatures in urban and rural areas of Incheon Metropolitan City, South Korea from 2015 to 2020: an observational study | PARASITES & VECTORS | 10.1186/s13071-021-05071-z | Vector-only study without a dengue-related outcome or context | The study focused on mosquito biology, ecology, abundance, distribution, or control without a substantive dengue-related outcome or analysis. |
| 2 | Riojas-Rodriguez | 2018 | Climate Change and Potential Health Effects in Mexican Children | ANNALS OF GLOBAL HEALTH | 10.29024/aogh.915 | Not dengue-specific | Neither the title nor the abstract treated dengue as a study subject or reported a dengue-related outcome or analysis. |
| 3 | Lau | 2020 | Impacts of Low Temperatures on Wolbachia (Rickettsiales: Rickettsiaceae)-Infected Aedes aegypti (Diptera: Culicidae) | JOURNAL OF MEDICAL ENTOMOLOGY | 10.1093/jme/tjaa074 | Vector-only study without a dengue-related outcome or context | The study focused on mosquito biology, ecology, abundance, distribution, or control without a substantive dengue-related outcome or analysis. |
| 4 | Petkova | 2015 | Climate Change and Health on the US Gulf Coast: Public Health Adaptation is Needed to Address Future Risks | INTERNATIONAL JOURNAL OF ENVIRONMENTAL RESEARCH AND PUBLIC HEALTH | 10.3390/ijerph120809342 | Not dengue-specific | Neither the title nor the abstract treated dengue as a study subject or reported a dengue-related outcome or analysis. |
| 5 | Senadeera | 2026 | Workplace health and safety under climate stress in Sri Lankan apparel SMEs | BMC PUBLIC HEALTH | 10.1186/s12889-026-26344-1 | General climate-health, disaster, or other-disease study without dengue-specific analysis | The study concerned general climate-health, disasters, multiple diseases, or another disease; dengue appeared only as background, in a disease list, or as a secondary context without an identifiable dengue-specific analysis. |
| 6 | Muturi | 2012 | Temperature and density-dependent effects of larval environment on Aedes aegypti competence for an alphavirus | JOURNAL OF VECTOR ECOLOGY | 10.1111/j.1948-7134.2012.00212.x | Vector-only study without a dengue-related outcome or context | The study focused on mosquito biology, ecology, abundance, distribution, or control without a substantive dengue-related outcome or analysis. |
| 7 | Salinas | 2021 | Temperatures Influence Susceptibility to Insecticides in Aedes aegypti and Aedes albopictus (Diptera: Culicidae) Mosquitoes | PATHOGENS | 10.3390/pathogens10080992 | Vector-only study without a dengue-related outcome or context | Dengue was mentioned only to describe the vector importance of the mosquito; the measured outcomes concerned mosquito biology, ecology, or control. |
| 8 | Boerlijst | 2024 | In the heat of the moment: Including realistic thermal fluctuations results in dramatically altered key population parameters | ECOLOGY AND EVOLUTION | 10.1002/ece3.70124 | Vector-only study without a dengue-related outcome or context | The study focused on mosquito biology, ecology, abundance, distribution, or control without a substantive dengue-related outcome or analysis. |
| 9 | Mabhaudhi | 2019 | The Water-Energy-Food Nexus as a Tool to Transform Rural Livelihoods and Well-Being in Southern Africa | INTERNATIONAL JOURNAL OF ENVIRONMENTAL RESEARCH AND PUBLIC HEALTH | 10.3390/ijerph16162970 | General climate-health, disaster, or other-disease study without dengue-specific analysis | The study concerned general climate-health, disasters, multiple diseases, or another disease; dengue appeared only as background, in a disease list, or as a secondary context without an identifiable dengue-specific analysis. |
| 10 | Lakhoo | 2024 | Protocol of an individual participant data meta-analysis to quantify the impact of high ambient temperatures on maternal and child health in Africa (HE2AT IPD) | BMJ OPEN | 10.1136/bmjopen-2023-077768 | Not dengue-specific | Neither the title nor the abstract treated dengue as a study subject or reported a dengue-related outcome or analysis. |
| 11 | Rahman | 2021 | COVID-19 pandemic, dengue epidemic, and climate change vulnerability in Bangladesh: Scenario assessment for strategic management and policy implications | ENVIRONMENTAL RESEARCH | 10.1016/j.envres.2020.110303 | General climate-health, disaster, or other-disease study without dengue-specific analysis | The study concerned general climate-health, disasters, multiple diseases, or another disease; dengue appeared only as background, in a disease list, or as a secondary context without an identifiable dengue-specific analysis. |
| 12 | Chaturvedi | 2026 | Extreme events and infant mortality: A mathematical modeling approach to understand climate-induced vulnerability in India | PHYSICS AND CHEMISTRY OF THE EARTH | 10.1016/j.pce.2025.104192 | Dengue mentioned only as background or an example | Dengue was mentioned only in the background, discussion, or as an example, without an identifiable dengue-specific analysis. |
| 13 | Sweileh | 2020 | Bibliometric analysis of peer-reviewed literature on climate change and human health with an emphasis on infectious diseases | GLOBALIZATION AND HEALTH | 10.1186/s12992-020-00576-1 | Dengue mentioned only as background or an example | The article discussed climate and health or another disease; dengue appeared only in the background or a disease list and was not analyzed separately. |
| 14 | Won | 2025 | Study of Aedes albopictus Hatching Rate by Low-Temperature Stress | ENTOMOLOGICAL RESEARCH | 10.1111/1748-5967.70018 | Vector-only study without a dengue-related outcome or context | The study focused on mosquito biology, ecology, abundance, distribution, or control without a substantive dengue-related outcome or analysis. |
| 15 | Ray | 2021 | Optimized ensemble deep learning framework for scalable forecasting of dynamics containing extreme events | CHAOS | 10.1063/5.0074213 | General climate-health, disaster, or other-disease study without dengue-specific analysis | The study concerned general climate-health, disasters, multiple diseases, or another disease; dengue appeared only as background, in a disease list, or as a secondary context without an identifiable dengue-specific analysis. |
| 16 | Zhou | 2010 | Effects of low temperature on the schistosome-transmitting snail Oncomelania hupensis and the implications of global climate change | MOLLUSCAN RESEARCH |  | Not dengue-specific | Neither the title nor the abstract treated dengue as a study subject or reported a dengue-related outcome or analysis. |
| 17 | Phommasone | 2015 | Temperature and the Field Stability of a Dengue Rapid Diagnostic Test in the Tropics | AMERICAN JOURNAL OF TROPICAL MEDICINE AND HYGIENE | 10.4269/ajtmh.15-0142 | Temperature/heat term represented a technical or biomedical condition, not meteorological exposure | Temperature or heat referred to assay stability, sample preservation, experimental heat stress, or a molecular mechanism rather than environmental weather or climate exposure. |
| 18 | Gonzalez-Rete | 2021 | Higher temperatures reduce the number of Trypanosoma cruzi parasites in the vector Triatoma pallidipennis | PARASITES & VECTORS | 10.1186/s13071-021-04872-6 | Not dengue-specific | Neither the title nor the abstract treated dengue as a study subject or reported a dengue-related outcome or analysis. |
| 19 | Achari | 2022 | Biochemical response of Aedes aegypti and Aedes albopictus mosquitoes after exposure to thermal stress and toxin of Bacillus thuringiensis israelensis | INTERNATIONAL JOURNAL OF TROPICAL INSECT SCIENCE | 10.1007/s42690-021-00587-4 | Vector-only study without a dengue-related outcome or context | The study focused on mosquito biology, ecology, abundance, distribution, or control without a substantive dengue-related outcome or analysis. |
| 20 | Hug | 2024 | Unexpected behavioural adaptation of yellow fever mosquitoes in response to high temperatures | SCIENTIFIC REPORTS | 10.1038/s41598-024-54374-5 | Vector-only study without a dengue-related outcome or context | The study focused on mosquito biology, ecology, abundance, distribution, or control without a substantive dengue-related outcome or analysis. |
| 21 | Jian | 2023 | Effects of constant temperature and daily fluctuating temperature on the transovarial transmission and life cycle of Aedes albopictus infected with Zika virus | FRONTIERS IN MICROBIOLOGY | 10.3389/fmicb.2022.1075362 | Vector-only study without a dengue-related outcome or context | The study focused on mosquito biology, ecology, abundance, distribution, or control without a substantive dengue-related outcome or analysis. |
| 22 | Haselkorn | 2013 | Density dynamics of diverse Spiroplasma strains naturally infecting different species of Drosophila | FLY | 10.4161/fly.25469 | Not dengue-specific | Neither the title nor the abstract treated dengue as a study subject or reported a dengue-related outcome or analysis. |
| 23 | Ulrich | 2016 | Heat Sensitivity of wMelWolbachia during Aedes aegypti Development | PLOS NEGLECTED TROPICAL DISEASES | 10.1371/journal.pntd.0004873 | Vector-only study without a dengue-related outcome or context | Dengue was mentioned only to describe the vector importance of the mosquito; the measured outcomes concerned mosquito biology, ecology, or control. |
| 24 | Agusto | 2020 | Optimal Control and Temperature Variations of Malaria Transmission Dynamics | COMPLEXITY | 10.1155/2020/5056432 | Vector-only study without a dengue-related outcome or context | The study focused on mosquito biology, ecology, abundance, distribution, or control without a substantive dengue-related outcome or analysis. |
| 25 | Costanzo | 2018 | Genetic and environmental influences on the size-fecundity relationship in Aedes albopictus (Diptera: Culicidae): Impacts on population growth estimates? | PLOS ONE | 10.1371/journal.pone.0201465 | Vector-only study without a dengue-related outcome or context | The study focused on mosquito biology, ecology, abundance, distribution, or control without a substantive dengue-related outcome or analysis. |
| 26 | Muniz | 2018 | Impact of short-term temperature challenges on the larvicidal activities of the entomopathogenic watermold Leptolegnia chapmanii against Aedes aegypti, and development on infected dead larvae | FUNGAL BIOLOGY | 10.1016/j.funbio.2017.10.002 | Vector-only study without a dengue-related outcome or context | The study focused on mosquito biology, ecology, abundance, distribution, or control without a substantive dengue-related outcome or analysis. |
| 27 | Gary | 2009 | Effect of sugar on male Anopheles gambiae mating performance, as modified by temperature, space, and body size | PARASITES & VECTORS | 10.1186/1756-3305-2-19 | Not dengue-specific | Neither the title nor the abstract treated dengue as a study subject or reported a dengue-related outcome or analysis. |
| 28 | Marini | 2016 | The Role of Climatic and Density Dependent Factors in Shaping Mosquito Population Dynamics: The Case of Culex pipiens in Northwestern Italy | PLOS ONE | 10.1371/journal.pone.0154018 | Vector-only study without a dengue-related outcome or context | The study focused on mosquito biology, ecology, abundance, distribution, or control without a substantive dengue-related outcome or analysis. |
| 29 | Ross | 2018 | Continued Susceptibility of the wMel Wolbachia Infection in Aedes aegypti to Heat Stress Following Field Deployment and Selection | INSECTS | 10.3390/insects9030078 | Vector-only study without a dengue-related outcome or context | The study focused on mosquito biology, ecology, abundance, distribution, or control without a substantive dengue-related outcome or analysis. |
| 30 | Boulanger | 2010 | A Europe-South America network for climate change assessment and impact studies | CLIMATIC CHANGE | 10.1007/s10584-009-9734-8 | Not dengue-specific | Neither the title nor the abstract treated dengue as a study subject or reported a dengue-related outcome or analysis. |
| 31 | Flor-Weiler | 2017 | CHARACTERIZATION OF TOLYPOCLADIUM CYLINDROSPORUM (HYPOCREALES: OPHIOCORDYCIPITACEAE) AND ITS IMPACT AGAINST AEDES AEGYPTI AND AEDES ALBOPICTUS EGGS AT LOW TEMPERATURE | JOURNAL OF THE AMERICAN MOSQUITO CONTROL ASSOCIATION | 10.2987/16-6596R.1 | Vector-only study without a dengue-related outcome or context | The study focused on mosquito biology, ecology, abundance, distribution, or control without a substantive dengue-related outcome or analysis. |
| 32 | Jeon | 2020 | Improvement of reproducibility and thermal stability of surface-enhanced Raman scattering-based lateral flow assay strips using silica-encapsulated gold nanoparticles | SENSORS AND ACTUATORS B-CHEMICAL | 10.1016/j.snb.2020.128521 | Temperature/heat term represented a technical or biomedical condition, not meteorological exposure | Temperature or heat referred to assay stability, sample preservation, experimental heat stress, or a molecular mechanism rather than environmental weather or climate exposure. |
| 33 | Pindi | 2021 | Identifying crucial E-protein residues responsible for unusual stability of Zika virus envelope | BIOPHYSICAL JOURNAL | 10.1016/j.bpj.2021.08.011 | Dengue mentioned only as background or an example | The article discussed climate and health or another disease; dengue appeared only in the background or a disease list and was not analyzed separately. |
| 34 | da Silva | 2021 | An epidemiological index for drought vulnerability in the Rio Grande do Norte State, Brazil | INTERNATIONAL JOURNAL OF BIOMETEOROLOGY | 10.1007/s00484-020-02034-4 | Not dengue-specific | Neither the title nor the abstract treated dengue as a study subject or reported a dengue-related outcome or analysis. |
| 35 | Thomas | 2012 | Low-temperature threshold for egg survival of a post-diapause and non-diapause European aedine strain, Aedes albopictus (Diptera: Culicidae) | PARASITES & VECTORS | 10.1186/1756-3305-5-100 | Vector-only study without a dengue-related outcome or context | The study focused on mosquito biology, ecology, abundance, distribution, or control without a substantive dengue-related outcome or analysis. |
| 36 | Tsai | 2018 | Critical low temperature for the survival of Aedes aegypti in Taiwan | PARASITES & VECTORS | 10.1186/s13071-017-2606-6 | Vector-only study without a dengue-related outcome or context | The study focused on mosquito biology, ecology, abundance, distribution, or control without a substantive dengue-related outcome or analysis. |
| 37 | Ritchie | 2022 | Transgenic cytoplasmic incompatibility persists across age and temperature variation in Drosophila melanogaster | ISCIENCE | 10.1016/j.isci.2022.105327 | Not dengue-specific | Neither the title nor the abstract treated dengue as a study subject or reported a dengue-related outcome or analysis. |
| 38 | Hoffmann | 2024 | Introduction of Aedes aegypti mosquitoes carrying wAlbB Wolbachia sharply decreases dengue incidence in disease hotspots | ISCIENCE | 10.1016/j.isci.2024.108942 | Vector-control or intervention study without a climate–dengue association | The study evaluated Wolbachia, insecticides, vector control, health education, or mosquito habitats without directly estimating an association between climate/meteorological exposure and a dengue outcome. |
| 39 | Larrieu | 2014 | Lessons raised by the major 2010 dengue epidemics in the French West Indies | ACTA TROPICA | 10.1016/j.actatropica.2013.11.023 | Dengue epidemiology, transmission, or social-factor study without climate-exposure analysis | The study focused on dengue outbreak description, spatial distribution, human mobility, public-health management, or social factors without directly analyzing a climate/meteorological exposure–dengue outcome association. |
| 40 | Moura | 2020 | What does not kill it does not always make it stronger: High temperatures in pyriproxyfen treatments produce Aedes aegypti adults with reduced longevity and smaller females | JOURNAL OF ASIA-PACIFIC ENTOMOLOGY | 10.1016/j.aspen.2020.04.001 | Vector-only study without a dengue-related outcome or context | The study focused on mosquito biology, ecology, abundance, distribution, or control without a substantive dengue-related outcome or analysis. |
| 41 | Ross | 2019 | Loss of cytoplasmic incompatibility in Wolbachia-infected Aedes aegypti under field conditions | PLOS NEGLECTED TROPICAL DISEASES | 10.1371/journal.pntd.0007357 | Vector-only study without a dengue-related outcome or context | Dengue was mentioned only to describe the vector importance of the mosquito; the measured outcomes concerned mosquito biology, ecology, or control. |
| 42 | Shocket | 2018 | Temperature explains broad patterns of Ross River virus transmission | ELIFE | 10.7554/eLife.37762 | Not dengue-specific | Neither the title nor the abstract treated dengue as a study subject or reported a dengue-related outcome or analysis. |
| 43 | Martin | 2022 | Gregarine parasites are adapted to mosquito winter diapause | PARASITES & VECTORS | 10.1186/s13071-022-05365-w | Vector-only study without a dengue-related outcome or context | The study focused on mosquito biology, ecology, abundance, distribution, or control without a substantive dengue-related outcome or analysis. |
| 44 | Lardeux | 2008 | A physiological time analysis of the duration of the gonotrophic cycle of Anopheles pseudopunctipennis and its implications for malaria transmission in Bolivia | MALARIA JOURNAL | 10.1186/1475-2875-7-141 | Vector-only study without a dengue-related outcome or context | The study focused on mosquito biology, ecology, abundance, distribution, or control without a substantive dengue-related outcome or analysis. |
| 45 | Palayam | 2018 | Structural insights into a multifunctional inhibitor, `AMTIN' from tubers of Alocasia macrorrhizos and its possible role in dengue protease (NS2B-NS3) inhibition | INTERNATIONAL JOURNAL OF BIOLOGICAL MACROMOLECULES | 10.1016/j.ijbiomac.2018.03.010 | Clinical, diagnostic, immunological, pathogenesis, or therapeutic dengue study | The study evaluated dengue clinical features, diagnosis, immune responses, molecular mechanisms, animal models, or treatment without using a climatic or meteorological factor as the exposure. |
| 46 | Vulu | 2024 | Geographic expansion of the introduced Aedes albopictus and other native Aedes species in the Democratic Republic of the Congo | PARASITES & VECTORS | 10.1186/s13071-024-06137-4 | Vector-only study without a dengue-related outcome or context | The study focused on mosquito biology, ecology, abundance, distribution, or control without a substantive dengue-related outcome or analysis. |
| 47 | Kim | 2021 | Temperature-dependent secretion of Zika virus envelope and non-structural protein 1 in mammalian cells for clinical applications | JOURNAL OF VIROLOGICAL METHODS | 10.1016/j.jviromet.2021.114175 | Vector-only study without a dengue-related outcome or context | The study focused on mosquito biology, ecology, abundance, distribution, or control without a substantive dengue-related outcome or analysis. |
| 48 | Liang | 2017 | Climate change and human infectious diseases: A synthesis of research findings from global and spatio-temporal perspectives | ENVIRONMENT INTERNATIONAL | 10.1016/j.envint.2017.03.011 | Not dengue-specific | Neither the title nor the abstract treated dengue as a study subject or reported a dengue-related outcome or analysis. |
| 49 | Lv | 2025 | Molecular cloning and functional characterizations of transient receptor potential A1 (TRPA1) in Aedes albopictus | PESTICIDE BIOCHEMISTRY AND PHYSIOLOGY | 10.1016/j.pestbp.2025.106295 | Vector-only study without a dengue-related outcome or context | The study focused on mosquito biology, ecology, abundance, distribution, or control without a substantive dengue-related outcome or analysis. |
| 50 | Konorov | 2022 | Genome Variation of Endosymbiotic Wolbachia in Introduced Populations of Asian Tiger Mosquito Aedes albopictus from Krasnodar Krai | RUSSIAN JOURNAL OF GENETICS | 10.1134/S1022795422020065 | Vector-only study without a dengue-related outcome or context | The study focused on mosquito biology, ecology, abundance, distribution, or control without a substantive dengue-related outcome or analysis. |
| 51 | Zhao | 2009 | Mitochondrial Gene Cytochrome b Developmental and Environmental Expression in Aedes aegypti (Diptera: Culicidae) | JOURNAL OF MEDICAL ENTOMOLOGY | 10.1603/033.046.0615 | Vector-only study without a dengue-related outcome or context | The study focused on mosquito biology, ecology, abundance, distribution, or control without a substantive dengue-related outcome or analysis. |
| 52 | Kai | 2024 | Evaluation of long-term preservation methods for viral RNA in mosquitoes at room temperature | JOURNAL OF VIROLOGICAL METHODS | 10.1016/j.jviromet.2024.114887 | Dengue mentioned only as background or an example | Dengue was mentioned only in the background, discussion, or as an example, without an identifiable dengue-specific analysis. |
| 53 | Mancini | 2024 | Wolbachia strain wAlbB shows favourable characteristics for dengue control use in Aedes aegypti from Burkina Faso | ENVIRONMENTAL MICROBIOLOGY | 10.1111/1462-2920.16588 | Vector-only study without a dengue-related outcome or context | The measured outcomes concerned vector biology or ecology rather than dengue transmission, incidence, or disease risk. |
| 54 | Baffoe | 2012 | Importance of temperature for the performance and biocontrol efficiency of the parasitoid Perilitus brevicollis (Hymenoptera: Braconidae) on Salix | BIOCONTROL | 10.1007/s10526-012-9443-5 | Not dengue-specific | Neither the title nor the abstract treated dengue as a study subject or reported a dengue-related outcome or analysis. |
| 55 | Ross | 2020 | Heatwaves cause fluctuations in wMel Wolbachia densities and frequencies in Aedes aegypti | PLOS NEGLECTED TROPICAL DISEASES | 10.1371/journal.pntd.0007958 | Vector-control or intervention study without a climate–dengue association | The study evaluated Wolbachia, insecticides, vector control, health education, or mosquito habitats without directly estimating an association between climate/meteorological exposure and a dengue outcome. |
| 56 | Thergarajan | 2019 | Effect of Blastocystis sp. in dengue patients-Increase in the treatment cost and exacerbation of symptoms | PLOS ONE | 10.1371/journal.pone.0211034 | Clinical, diagnostic, immunological, pathogenesis, or therapeutic dengue study | The study evaluated dengue clinical features, diagnosis, immune responses, molecular mechanisms, animal models, or treatment without using a climatic or meteorological factor as the exposure. |
| 57 | Mall | 2017 | Impact of climate variability on human health: A pilot study in tertiary care hospital of Eastern Uttar Pradesh, India | MAUSAM |  | Dengue mentioned only as background or an example | Dengue was mentioned only in the background, discussion, or as an example, without an identifiable dengue-specific analysis. |
| 58 | Masuh | 2008 | Aedes aegypti (Diptera: Culicidae):: monitoring of populations to improve control strategies in Argentina | PARASITOLOGY RESEARCH | 10.1007/s00436-008-0945-0 | Vector-only study without a dengue-related outcome or context | Dengue was mentioned only to describe the vector importance of the mosquito; the measured outcomes concerned mosquito biology, ecology, or control. |
| 59 | Zeng | 2022 | A standalone incompatible insect technique enables mosquito suppression in the urban subtropics | COMMUNICATIONS BIOLOGY | 10.1038/s42003-022-04332-6 | Vector-only study without a dengue-related outcome or context | Dengue was mentioned only to describe the vector importance of the mosquito; the measured outcomes concerned mosquito biology, ecology, or control. |
| 60 | Luehken | 2024 | High vector competence for chikungunya virus but heavily reduced locomotor activity of Aedes albopictus from Germany at low temperatures | PARASITES & VECTORS | 10.1186/s13071-024-06594-x | Vector-only study without a dengue-related outcome or context | The study focused on mosquito biology, ecology, abundance, distribution, or control without a substantive dengue-related outcome or analysis. |
| 61 | Lopes | 2023 | Exploring the impact of temperature on the efficacy of replacing a wild Aedes aegypti population by a Wolbachia-carrying one | APPLIED MATHEMATICAL MODELLING | 10.1016/j.apm.2023.07.007 | Vector-only study without a dengue-related outcome or context | The study focused on mosquito biology, ecology, abundance, distribution, or control without a substantive dengue-related outcome or analysis. |
| 62 | Culbert | 2019 | Investigating the impact of chilling temperature on male Aedes aegypti and Aedes albopictus survival | PLOS ONE | 10.1371/journal.pone.0221822 | Vector-only study without a dengue-related outcome or context | The study focused on mosquito biology, ecology, abundance, distribution, or control without a substantive dengue-related outcome or analysis. |
| 63 | Hendy | 2020 | The vertical stratification of potential bridge vectors of mosquito-borne viruses in a central Amazonian forest bordering Manaus, Brazil | SCIENTIFIC REPORTS | 10.1038/s41598-020-75178-3 | Vector-only study without a dengue-related outcome or context | The study focused on mosquito biology, ecology, abundance, distribution, or control without a substantive dengue-related outcome or analysis. |
| 64 | Wang | 2010 | Hyperthermia induces platelet apoptosis and glycoprotein Ibα ectodomain shedding | PLA℡ETS | 10.3109/09537100903443949 | Temperature/heat term represented a technical or biomedical condition, not meteorological exposure | Temperature or heat referred to assay stability, sample preservation, experimental heat stress, or a molecular mechanism rather than environmental weather or climate exposure. |
| 65 | Pekanska | 2025 | Elevated developmental temperatures below the lethal limit reduce Aedes aegypti fertility | JOURNAL OF EXPERIMENTAL BIOLOGY | 10.1242/jeb.249803 | Vector-only study without a dengue-related outcome or context | Dengue was mentioned only to describe the vector importance of the mosquito; the measured outcomes concerned mosquito biology, ecology, or control. |
| 66 | Byttebier | 2014 | Hatching Response of Aedes aegypti (Diptera: Culicidae) Eggs at Low Temperatures: Effects of Hatching Media and Storage Conditions | JOURNAL OF MEDICAL ENTOMOLOGY | 10.1603/ME13066 | Vector-only study without a dengue-related outcome or context | The study focused on mosquito biology, ecology, abundance, distribution, or control without a substantive dengue-related outcome or analysis. |
| 67 | Lahondere | 2012 | Mosquitoes Cool Down during Blood Feeding to Avoid Overheating | CURRENT BIOLOGY | 10.1016/j.cub.2011.11.029 | Vector-only study without a dengue-related outcome or context | The study focused on mosquito biology, ecology, abundance, distribution, or control without a substantive dengue-related outcome or analysis. |
| 68 | Huang | 2026 | Dynamics and suppression paradox of a stage-structured model for mosquito control | JOURNAL OF MATHEMATICAL BIOLOGY | 10.1007/s00285-026-02387-1 | Vector-only study without a dengue-related outcome or context | The study focused on mosquito biology, ecology, abundance, distribution, or control without a substantive dengue-related outcome or analysis. |
| 69 | Fernandes Silva Chagas do Nascimento | 2025 | Systematic Review of the Ovitrap Surveillance of Aedes Mosquitoes in Brazil (2012-2022) | TROPICAL MEDICINE AND INFECTIOUS DISEASE | 10.3390/tropicalmed10080212 | Vector-only study without a dengue-related outcome or context | Dengue was mentioned only to describe the vector importance of the mosquito; the measured outcomes concerned mosquito biology, ecology, or control. |
| 70 | Kress | 2017 | Cold tolerance of the Asian tiger mosquito Aedes albopictus and its response to epigenetic alterations | JOURNAL OF INSECT PHYSIOLOGY | 10.1016/j.jinsphys.2017.04.003 | Vector-only study without a dengue-related outcome or context | The study focused on mosquito biology, ecology, abundance, distribution, or control without a substantive dengue-related outcome or analysis. |
| 71 | Salmon-Rousseau | 2016 | Hajj-associated infections | MEDECINE ET MALADIES INFECTIEUSES | 10.1016/j.medmal.2016.04.002 | General climate-health, disaster, or other-disease study without dengue-specific analysis | The study concerned general climate-health, disasters, multiple diseases, or another disease; dengue appeared only as background, in a disease list, or as a secondary context without an identifiable dengue-specific analysis. |
| 72 | Chen | 2024 | Roles of a newly lethal cuticular structural protein, AaCPR100A, and its upstream interaction protein, G12-like, in Aedes aegypti | INTERNATIONAL JOURNAL OF BIOLOGICAL MACROMOLECULES | 10.1016/j.ijbiomac.2024.131704 | Vector-only study without a dengue-related outcome or context | The study focused on mosquito biology, ecology, abundance, distribution, or control without a substantive dengue-related outcome or analysis. |
| 73 | Devillers | 2020 | Fate and ecotoxicological effects of pyriproxyfen in aquatic ecosystems | ENVIRONMENTAL SCIENCE AND POLLUTION RESEARCH | 10.1007/s11356-020-08345-8 | Not dengue-specific | Neither the title nor the abstract treated dengue as a study subject or reported a dengue-related outcome or analysis. |
| 74 | Silva Pessoa Vieira | 2020 | Ecological aspects of potential arbovirus vectors (Diptera: Culicidae) in an urban landscape of Southern Amazon, Brazil | ACTA TROPICA | 10.1016/j.actatropica.2019.105276 | Vector-only study without a dengue-related outcome or context | The study focused on mosquito biology, ecology, abundance, distribution, or control without a substantive dengue-related outcome or analysis. |
| 75 | Kaczmarek | 2021 | The metabolism and role of free fatty acids in key physiological processes in insects of medical, veterinary and forensic importance | PEERJ | 10.7717/peerj.12563 | Not dengue-specific | Neither the title nor the abstract treated dengue as a study subject or reported a dengue-related outcome or analysis. |
| 76 | Ekwudu | 2020 | Wolbachia strain wAlbB blocks replication of flaviviruses and alphaviruses in mosquito cell culture | PARASITES & VECTORS | 10.1186/s13071-020-3936-3 | Vector-only study without a dengue-related outcome or context | Dengue was mentioned only to describe the vector importance of the mosquito; the measured outcomes concerned mosquito biology, ecology, or control. |
| 77 | Brustolin | 2016 | Culex pipiens and Stegomyia albopicta (=Aedes albopictus) populations as vectors for lineage 1 and 2 West Nile virus in Europe | MEDICAL AND VETERINARY ENTOMOLOGY | 10.1111/mve.12164 | Not dengue-specific | Neither the title nor the abstract treated dengue as a study subject or reported a dengue-related outcome or analysis. |
| 78 | Rijkers | 2026 | Climate change and the immune system | EXPERT REVIEW OF CLINICAL IMMUNOLOGY | 10.1080/1744666X.2026.2625969 | Dengue mentioned only as background or an example | Dengue was mentioned only in the background, discussion, or as an example, without an identifiable dengue-specific analysis. |
| 79 | Farjana | 2013 | Multiple Blood Feeding and Host-Seeking Behavior in Aedes aegypti and Aedes albopictus (Diptera: Culicidae) | JOURNAL OF MEDICAL ENTOMOLOGY | 10.1603/ME12146 | Vector-only study without a dengue-related outcome or context | The study focused on mosquito biology, ecology, abundance, distribution, or control without a substantive dengue-related outcome or analysis. |
| 80 | Munoz | 2016 | Analyzing climate variations at multiple timescales can guide Zika virus response measures | GIGASCIENCE | 10.1186/s13742-016-0146-1 | Not dengue-specific | Neither the title nor the abstract treated dengue as a study subject or reported a dengue-related outcome or analysis. |
| 81 | Orozco-Gonzales | 2024 | Comparing the long-term persistence of different Wolbachia strains after the release of bacteria-carrying mosquitoes | MATHEMATICAL BIOSCIENCES | 10.1016/j.mbs.2024.109190 | Vector-only study without a dengue-related outcome or context | The study focused on mosquito biology, ecology, abundance, distribution, or control without a substantive dengue-related outcome or analysis. |
| 82 | Moras | 2022 | Early diagnostic markers in predicting the severity of dengue disease | 3 BIOTECH | 10.1007/s13205-022-03334-9 | Clinical, diagnostic, immunological, pathogenesis, or therapeutic dengue study | The study evaluated dengue clinical features, diagnosis, immune responses, molecular mechanisms, animal models, or treatment without using a climatic or meteorological factor as the exposure. |
| 83 | Hikke | 2014 | Salmonid alphavirus replication in mosquito cells: towards a novel vaccine production system | MICROBIAL BIOTECHNOLOGY | 10.1111/1751-7915.12100 | Not dengue-specific | Neither the title nor the abstract treated dengue as a study subject or reported a dengue-related outcome or analysis. |
| 84 | Soliman | 2020 | Ensemble forecasting of the Zika space-time spread with topological data analysis | ENVIRONMETRICS | 10.1002/env.2629 | Vector-only study without a dengue-related outcome or context | The study focused on mosquito biology, ecology, abundance, distribution, or control without a substantive dengue-related outcome or analysis. |
| 85 | De Majo | 2021 | Comparative Study on the Thermal Performance of Three Aedes aegypti (Diptera: Culicidae) Populations From Argentina | JOURNAL OF MEDICAL ENTOMOLOGY | 10.1093/jme/tjab017 | Vector-only study without a dengue-related outcome or context | The study focused on mosquito biology, ecology, abundance, distribution, or control without a substantive dengue-related outcome or analysis. |
| 86 | Thergarajan | 2018 | In vitro and in vivo thermal stress induces proliferation of Blastocystis sp. | PARASITOLOGY RESEARCH | 10.1007/s00436-017-5688-3 | Dengue mentioned only as background or an example | Dengue was mentioned only in the background, discussion, or as an example, without an identifiable dengue-specific analysis. |
| 87 | Macchioni | 2019 | Azadirachta indica (Sapindales: Meliaceae) Neem Oil as a Repellent Against Aedes albopictus (Diptera: Culicidae) Mosquitoes | JOURNAL OF INSECT SCIENCE | 10.1093/jisesa/iez111 | Vector-only study without a dengue-related outcome or context | The study focused on mosquito biology, ecology, abundance, distribution, or control without a substantive dengue-related outcome or analysis. |
| 88 | Mossenta | 2017 | Role of N-glycosylation on Zika virus E protein secretion, viral assembly and infectivity | BIOCHEMICAL AND BIOPHYSICAL RESEARCH COMMUNICATIONS | 10.1016/j.bbrc.2017.01.022 | Dengue mentioned only as background or an example | The article discussed climate and health or another disease; dengue appeared only in the background or a disease list and was not analyzed separately. |
| 89 | Ross | 2021 | A wAlbB Wolbachia Transinfection Displays Stable Phenotypic Effects across Divergent Aedes aegypti Mosquito Backgrounds | APPLIED AND ENVIRONMENTAL MICROBIOLOGY | 10.1128/AEM.01264-21 | Vector-only study without a dengue-related outcome or context | Dengue was mentioned only to describe the vector importance of the mosquito; the measured outcomes concerned mosquito biology, ecology, or control. |
| 90 | Lee | 2024 | Temperature and photoperiod differentially impact maternal phenotypes in diapause egg-laying Aedes albopictus mosquitoes | PLOS NEGLECTED TROPICAL DISEASES | 10.1371/journal.pntd.0012626 | Vector-only study without a dengue-related outcome or context | The study focused on mosquito biology, ecology, abundance, distribution, or control without a substantive dengue-related outcome or analysis. |
| 91 | Kim | 2018 | Analysis of habitat characteristics of mosquitoes in Danwon-gu, Ansan city, Korea, based on civil complaint data | ENTOMOLOGICAL RESEARCH | 10.1111/1748-5967.12330 | Vector-only study without a dengue-related outcome or context | The study focused on mosquito biology, ecology, abundance, distribution, or control without a substantive dengue-related outcome or analysis. |
| 92 | Wimalasiri-Yapa | 2019 | Chikungunya Virus Transmission at Low Temperature by Aedes albopictus Mosquitoes | PATHOGENS | 10.3390/pathogens8030149 | Not dengue-specific | Neither the title nor the abstract treated dengue as a study subject or reported a dengue-related outcome or analysis. |
| 93 | Chen | 2020 | Elizabethkingia anophelis: Physiologic and Transcriptomic Responses to Iron Stress | FRONTIERS IN MICROBIOLOGY | 10.3389/fmicb.2020.00804 | Vector-only study without a dengue-related outcome or context | The study focused on mosquito biology, ecology, abundance, distribution, or control without a substantive dengue-related outcome or analysis. |
| 94 | Verhulst | 2020 | Thermal preferences of subtropical Aedes aegypti and temperate Ae. japonicus mosquitoes | JOURNAL OF THERMAL BIOLOGY | 10.1016/j.jtherbio.2020.102637 | Vector-only study without a dengue-related outcome or context | The study focused on mosquito biology, ecology, abundance, distribution, or control without a substantive dengue-related outcome or analysis. |
| 95 | Gonzalez-Rizo | 2019 | Effect of Chlorine and Temperature on Larvicidal Activity of Cuban Bacillus thuringiensis Isolates | JOURNAL OF ARTHROPOD-BORNE DISEASES |  | Vector-only study without a dengue-related outcome or context | The study focused on mosquito biology, ecology, abundance, distribution, or control without a substantive dengue-related outcome or analysis. |
| 96 | Moura | 2021 | Be quick or be dead: high temperatures reduce Aedes aegypti (Diptera: Culicidae) larval development time and pyriproxyfen larvicide efficiency in laboratory conditions | INTERNATIONAL JOURNAL OF TROPICAL INSECT SCIENCE | 10.1007/s42690-020-00367-6 | Vector-only study without a dengue-related outcome or context | The study focused on mosquito biology, ecology, abundance, distribution, or control without a substantive dengue-related outcome or analysis. |
| 97 | Tran | 2023 | Climate change and human health in Vietnam: a systematic review and additional analyses on current impacts, future risk, and adaptation | LANCET REGIONAL HEALTH-WESTERN PACIFIC | 10.1016/j.lanwpc.2023.100943 | Not dengue-specific | Neither the title nor the abstract treated dengue as a study subject or reported a dengue-related outcome or analysis. |
| 98 | Caragata | 2020 | A Nonlive Preparation of Chromobacterium sp. Panama (Csp_P) Is a Highly Effective Larval Mosquito Biopesticide | APPLIED AND ENVIRONMENTAL MICROBIOLOGY | 10.1128/AEM.00240-20 | Vector-only study without a dengue-related outcome or context | Dengue was mentioned only to describe the vector importance of the mosquito; the measured outcomes concerned mosquito biology, ecology, or control. |
| 99 | Adelman | 2013 | Cooler Temperatures Destabilize RNA Interference and Increase Susceptibility of Disease Vector Mosquitoes to Viral Infection | PLOS NEGLECTED TROPICAL DISEASES | 10.1371/journal.pntd.0002239 | Vector-only study without a dengue-related outcome or context | The study focused on mosquito biology, ecology, abundance, distribution, or control without a substantive dengue-related outcome or analysis. |
| 100 | Shen | 2015 | Multiple Sources of Infection and Potential Endemic Characteristics of the Large Outbreak of Dengue in Guangdong in 2014 | SCIENTIFIC REPORTS | 10.1038/srep16913 | Dengue epidemiology, transmission, or social-factor study without climate-exposure analysis | The study focused on dengue outbreak description, spatial distribution, human mobility, public-health management, or social factors without directly analyzing a climate/meteorological exposure–dengue outcome association. |
| 101 | Trzebny | 2024 | High temperatures and low humidity promote the occurrence of microsporidians (Microsporidia) in mosquitoes (Culicidae) | PARASITES & VECTORS | 10.1186/s13071-024-06254-0 | Not dengue-specific | Neither the title nor the abstract treated dengue as a study subject or reported a dengue-related outcome or analysis. |
| 102 | Kontopoulos | 2018 | Use and misuse of temperature normalisation in meta-analyses of thermal responses of biological traits | PEERJ | 10.7717/peerj.4363 | Not dengue-specific | Neither the title nor the abstract treated dengue as a study subject or reported a dengue-related outcome or analysis. |
| 103 | Diptyanusa | 2020 | Implementation of geographical information systems for the study of diseases caused by vector-borne arboviruses in Southeast Asia: A review based on the publication record | GEOSPATIAL HEALTH | 10.4081/gh.2020.862 | Vector-only study without a dengue-related outcome or context | The measured outcomes concerned vector biology or ecology rather than dengue transmission, incidence, or disease risk. |
| 104 | Devlin | 2026 | Substantial cold tolerance in all life stages of Culicoides nubeculosus (Diptera: Ceratopogonidae) | JOURNAL OF MEDICAL ENTOMOLOGY | 10.1093/jme/tjag020 | Not dengue-specific | Neither the title nor the abstract treated dengue as a study subject or reported a dengue-related outcome or analysis. |
| 105 | Lindauer | 2020 | Daily fluctuating temperatures decrease growth and reproduction rate of a lethal amphibian fungal pathogen in culture | BMC ECOLOGY | 10.1186/s12898-020-00286-7 | Not dengue-specific | Neither the title nor the abstract treated dengue as a study subject or reported a dengue-related outcome or analysis. |
| 106 | Sivan | 2017 | Expression of heat shock proteins (HSPs) in Aedes aegypti (L) and Aedes albopictus (Skuse) (Diptera: Culicidae) larvae in response to thermal stress | ACTA TROPICA | 10.1016/j.actatropica.2016.12.017 | Vector-only study without a dengue-related outcome or context | The study focused on mosquito biology, ecology, abundance, distribution, or control without a substantive dengue-related outcome or analysis. |
| 107 | Ferraguti | 2024 | Landscape and climatic factors shaping mosquito abundance and species composition in southern Spain: A machine learning approach to the study of vector ecology | ECOLOGICAL INFORMATICS | 10.1016/j.ecoinf.2024.102860 | Vector-only study without a dengue-related outcome or context | The study focused on mosquito biology, ecology, abundance, distribution, or control without a substantive dengue-related outcome or analysis. |
| 108 | Barmak | 2016 | Modelling dengue epidemic spreading with human mobility | PHYSICA A-STATISTICAL MECHANICS AND ITS APPLICATIONS | 10.1016/j.physa.2015.12.015 | Dengue epidemiology, transmission, or social-factor study without climate-exposure analysis | The study focused on dengue outbreak description, spatial distribution, human mobility, public-health management, or social factors without directly analyzing a climate/meteorological exposure–dengue outcome association. |
| 109 | Phung | 2018 | The effects of socioecological factors on variation of communicable diseases: A multiple-disease study at the national scale of Vietnam | PLOS ONE | 10.1371/journal.pone.0193246 | Dengue mentioned only as background or an example | Dengue was mentioned only in the background, discussion, or as an example, without an identifiable dengue-specific analysis. |
| 110 | Ioannou | 2024 | Insecticide resistant mosquitoes remain thermal stress resistant, without loss of thermal plasticity | SCIENCE OF THE TOTAL ENVIRONMENT | 10.1016/j.scitotenv.2023.169443 | Vector-only study without a dengue-related outcome or context | The study focused on mosquito biology, ecology, abundance, distribution, or control without a substantive dengue-related outcome or analysis. |
| 111 | Kristan | 2018 | Effect of environmental variables and kdr resistance genotype on survival probability and infection rates in Anopheles gambiae (s.s.) | PARASITES & VECTORS | 10.1186/s13071-018-3150-8 | Vector-only study without a dengue-related outcome or context | The study focused on mosquito biology, ecology, abundance, distribution, or control without a substantive dengue-related outcome or analysis. |
| 112 | Ueda | 2018 | A sensitive and efficient procedure for the high-throughput determination of nine urinary metabolites of pyrethroids by GC-MS/MS and its application in a sample of Japanese children | ANALYTICAL AND BIOANALYTICAL CHEMISTRY | 10.1007/s00216-018-1229-x | Not dengue-specific | Neither the title nor the abstract treated dengue as a study subject or reported a dengue-related outcome or analysis. |
| 113 | Acuna | 2025 | Impact of the COVID-19 Pandemic on a Biodiversity Biobank: Response and Adaptation | BIOPRESERVATION AND BIOBANKING | 10.1177/19475535251380706 | Dengue mentioned only as background or an example | The article discussed climate and health or another disease; dengue appeared only in the background or a disease list and was not analyzed separately. |
| 114 | Baz | 2025 | Efficacy of mosquito repellent finishes on polyester fabrics using four essential oils against the vector of the West Nile virus, Culex pipiens (Diptera: Culicidae) | JOURNAL OF THE TEXTILE INSTITUTE | 10.1080/00405000.2024.2331846 | Vector-only study without a dengue-related outcome or context | The study focused on mosquito biology, ecology, abundance, distribution, or control without a substantive dengue-related outcome or analysis. |
| 115 | Zhang | 2025 | LUPP-LAMP: one-pot multiplatform universal LAMP assay for simultaneous detection and genotyping of DENV and ZIKV | JOURNAL OF NANOBIOTECHNOLOGY | 10.1186/s12951-025-03581-x | Vector-only study without a dengue-related outcome or context | The measured outcomes concerned vector biology or ecology rather than dengue transmission, incidence, or disease risk. |
| 116 | Zakaria | 2025 | Perceptions of coastal dwellers about the effects of extreme temperature and saline water on human health: evidence from Bangladesh | FRONTIERS IN PUBLIC HEALTH | 10.3389/fpubh.2025.1451933 | General climate-health, disaster, or other-disease study without dengue-specific analysis | The study concerned general climate-health, disasters, multiple diseases, or another disease; dengue appeared only as background, in a disease list, or as a secondary context without an identifiable dengue-specific analysis. |
| 117 | Gardini Sanches Palasio | 2023 | Zika, chikungunya and co-occurrence in Brazil: space-time clusters and associated environmental-socioeconomic factors | SCIENTIFIC REPORTS | 10.1038/s41598-023-42930-4 | Not dengue-specific | Neither the title nor the abstract treated dengue as a study subject or reported a dengue-related outcome or analysis. |
| 118 | Ferreira | 2020 | Temperature Dramatically Shapes Mosquito Gene Expression With Consequences for Mosquito-Zika Virus Interactions | FRONTIERS IN MICROBIOLOGY | 10.3389/fmicb.2020.00901 | Not dengue-specific | Neither the title nor the abstract treated dengue as a study subject or reported a dengue-related outcome or analysis. |
| 119 | Ziegler | 2022 | Video analysis of the locomotory behaviour of Aedes aegypti and Ae. japonicus mosquitoes under different temperature regimes in a laboratory setting | JOURNAL OF THERMAL BIOLOGY | 10.1016/j.jtherbio.2022.103205 | Vector-only study without a dengue-related outcome or context | The study focused on mosquito biology, ecology, abundance, distribution, or control without a substantive dengue-related outcome or analysis. |
| 120 | Phung | 2018 | Spatial variation of heat-related morbidity: A hierarchical Bayesian analysis in multiple districts of the Mekong Delta Region | SCIENCE OF THE TOTAL ENVIRONMENT | 10.1016/j.scitotenv.2018.05.131 | Not dengue-specific | Neither the title nor the abstract treated dengue as a study subject or reported a dengue-related outcome or analysis. |
| 121 | Tuno | 2020 | Climate Change May Restrict the Predation Efficiency of Mesocyclops aspericornis (Copepoda: Cyclopidae) on Aedes aegypti (Diptera: Culicidae) Larvae | INSECTS | 10.3390/insects11050307 | Vector-only study without a dengue-related outcome or context | Dengue was mentioned only to describe the vector importance of the mosquito; the measured outcomes concerned mosquito biology, ecology, or control. |
| 122 | Alomar | 2021 | Juvenile hormone analog enhances Zika virus infection in Aedes aegypti | SCIENTIFIC REPORTS | 10.1038/s41598-021-00432-1 | Vector-only study without a dengue-related outcome or context | Dengue was mentioned only to describe the vector importance of the mosquito; the measured outcomes concerned mosquito biology, ecology, or control. |
| 123 | Lumley | 2018 | Competence of mosquitoes native to the United Kingdom to support replication and transmission of Rift Valley fever virus | PARASITES & VECTORS | 10.1186/s13071-018-2884-7 | Vector-only study without a dengue-related outcome or context | The study focused on mosquito biology, ecology, abundance, distribution, or control without a substantive dengue-related outcome or analysis. |
| 124 | Alvarez-Duhart | 2026 | Temperature variability increases Trypanosoma cruzi load but not the extrinsic incubation period in Triatoma infestans | PARASITES & VECTORS | 10.1186/s13071-026-07270-y | Vector-only study without a dengue-related outcome or context | The study focused on mosquito biology, ecology, abundance, distribution, or control without a substantive dengue-related outcome or analysis. |
| 125 | Hossain | 2024 | Impact of chilling temperature and its exposure period on survival, flight ability and sterility of Aedes aegypti (Linnaeus) (Diptera: Culicidae) | INTERNATIONAL JOURNAL OF TROPICAL INSECT SCIENCE | 10.1007/s42690-024-01332-3 | Vector-only study without a dengue-related outcome or context | Dengue was mentioned only to describe the vector importance of the mosquito; the measured outcomes concerned mosquito biology, ecology, or control. |
| 126 | Swain | 2009 | Impact of temperature on susceptible and resistant strains of Culex quinquefasciatus to synthetic pyrethroids | ACTA TROPICA | 10.1016/j.actatropica.2009.08.020 | Not dengue-specific | Neither the title nor the abstract treated dengue as a study subject or reported a dengue-related outcome or analysis. |
| 127 | Ogunlade | 2021 | A Review: Aedes-Borne Arboviral Infections, Controls and Wolbachia-Based Strategies | VACCINES | 10.3390/vaccines9010032 | Dengue mentioned only as background or an example | Dengue was mentioned only in the background, discussion, or as an example, without an identifiable dengue-specific analysis. |
| 128 | Gale | 2019 | Towards a thermodynamic mechanistic model for the effect of temperature on arthropod vector competence for transmission of arboviruses | MICROBIAL RISK ANALYSIS | 10.1016/j.mran.2019.03.001 | Dengue mentioned only as background or an example | Dengue was mentioned only in the background, discussion, or as an example, without an identifiable dengue-specific analysis. |
| 129 | Danforth | 2016 | The Impact of Cycling Temperature on the Transmission of West Nile Virus | JOURNAL OF MEDICAL ENTOMOLOGY | 10.1093/jme/tjw013 | Dengue mentioned only as background or an example | The article discussed climate and health or another disease; dengue appeared only in the background or a disease list and was not analyzed separately. |
| 130 | Vega-Rua | 2015 | Chikungunya Virus Transmission Potential by Local Aedes Mosquitoes in the Americas and Europe | PLOS NEGLECTED TROPICAL DISEASES | 10.1371/journal.pntd.0003780 | Not dengue-specific | Neither the title nor the abstract treated dengue as a study subject or reported a dengue-related outcome or analysis. |
| 131 | Spanoudis | 2019 | Effect of Temperature on Biological Parameters of the West Nile Virus Vector Culex pipiens form `molestus' (Diptera: Culicidae) in Greece: Constant vs Fluctuating Temperatures | JOURNAL OF MEDICAL ENTOMOLOGY | 10.1093/jme/tjy224 | Vector-only study without a dengue-related outcome or context | The study focused on mosquito biology, ecology, abundance, distribution, or control without a substantive dengue-related outcome or analysis. |
| 132 | Maffey | 2022 | City puzzles: Does urban land scape affect genetic population structure in Aedes aegypti? | PLOS NEGLECTED TROPICAL DISEASES | 10.1371/journal.pntd.0010549 | Vector-only study without a dengue-related outcome or context | Dengue was mentioned only to describe the vector importance of the mosquito; the measured outcomes concerned mosquito biology, ecology, or control. |
| 133 | Agyekum | 2022 | Relationship between temperature and Anopheles gambiae sensu lato mosquitoes' susceptibility to pyrethroids and expression of metabolic enzymes | PARASITES & VECTORS | 10.1186/s13071-022-05273-z | Vector-only study without a dengue-related outcome or context | The study focused on mosquito biology, ecology, abundance, distribution, or control without a substantive dengue-related outcome or analysis. |
| 134 | Hernandez-Triana | 2021 | Oral susceptibility of aedine and culicine mosquitoes (Diptera: Culicidae) to Batai Orthobunyavirus | PARASITES & VECTORS | 10.1186/s13071-021-05070-0 | Not dengue-specific | Neither the title nor the abstract treated dengue as a study subject or reported a dengue-related outcome or analysis. |
| 135 | Jemal | 2018 | Combining GIS application and climatic factors CrossMark for mosquito control in Eastern Province, Saudi Arabia | SAUDI JOURNAL OF BIOLOGICAL SCIENCES | 10.1016/j.sjbs.2016.04.001 | Vector-only study without a dengue-related outcome or context | The study focused on mosquito biology, ecology, abundance, distribution, or control without a substantive dengue-related outcome or analysis. |
| 136 | Agyekum | 2021 | A Systematic Review of the Effects of Temperature on Anopheles Mosquito Development and Survival: Implications for Malaria Control in a Future Warmer Climate | INTERNATIONAL JOURNAL OF ENVIRONMENTAL RESEARCH AND PUBLIC HEALTH | 10.3390/ijerph18147255 | Vector-only study without a dengue-related outcome or context | The study focused on mosquito biology, ecology, abundance, distribution, or control without a substantive dengue-related outcome or analysis. |
| 137 | Jacob | 2026 | Diurnal fluctuating temperature and larval resource level interact to influence the life history and behaviour of disease-transmitting mosquitoes | PARASITES & VECTORS | 10.1186/s13071-026-07313-4 | Vector-only study without a dengue-related outcome or context | The study focused on mosquito biology, ecology, abundance, distribution, or control without a substantive dengue-related outcome or analysis. |
| 138 | Mbaika | 2016 | Vector competence of Aedes aegypti in transmitting Chikungunya virus: effects and implications of extrinsic incubation temperature on dissemination and infection rates | VIROLOGY JOURNAL | 10.1186/s12985-016-0566-7 | Vector-only study without a dengue-related outcome or context | The study focused on mosquito biology, ecology, abundance, distribution, or control without a substantive dengue-related outcome or analysis. |
| 139 | Zhang | 2020 | Toward implementation of combined incompatible and sterile insect techniques for mosquito control: Optimized chilling conditions for handlingAedes albopictusmale adults prior to release | PLOS NEGLECTED TROPICAL DISEASES | 10.1371/journal.pntd.0008561 | Vector-only study without a dengue-related outcome or context | Dengue was mentioned only to describe the vector importance of the mosquito; the measured outcomes concerned mosquito biology, ecology, or control. |
| 140 | Beggs | 2024 | The 2023 report of the MJA-Lancet Countdown on health and climate change: sustainability needed in Australia's health care sector | MEDICAL JOURNAL OF AUSTRALIA | 10.5694/mja2.52245 | Not dengue-specific | Neither the title nor the abstract treated dengue as a study subject or reported a dengue-related outcome or analysis. |
| 141 | Pena-Garcia | 2019 | Correlation of the basic reproduction number (R0) and eco-environmental variables in Colombian municipalities with chikungunya outbreaks during 2014-2016 | PLOS NEGLECTED TROPICAL DISEASES | 10.1371/journal.pntd.0007878 | Not dengue-specific | Neither the title nor the abstract treated dengue as a study subject or reported a dengue-related outcome or analysis. |
| 142 | Canyon | 2016 | Forecasted Impact of Climate Change on Infectious Disease and leHealth Security in Hawaii by 2050 | DISASTER MEDICINE AND PUBLIC HEALTH PREPAREDNESS | 10.1017/dmp.2016.73 | Vector-only study without a dengue-related outcome or context | The study focused on mosquito biology, ecology, abundance, distribution, or control without a substantive dengue-related outcome or analysis. |
| 143 | Caminade | 2019 | Impact of recent and future climate change on vector-borne diseases | ANNALS OF THE NEW YORK ACADEMY OF SCIENCES | 10.1111/nyas.13950 | Not dengue-specific | Neither the title nor the abstract treated dengue as a study subject or reported a dengue-related outcome or analysis. |
| 144 | Bouzid | 2013 | The Effectiveness of Public Health Interventions to Reduce the Health Impact of Climate Change: A Systematic Review of Systematic Reviews | PLOS ONE | 10.1371/journal.pone.0062041 | Not dengue-specific | Neither the title nor the abstract treated dengue as a study subject or reported a dengue-related outcome or analysis. |
| 145 | Andersen | 2015 | The effects of the El Nino Southern Oscillation on skin and skin-related diseases: a message from the International Society of Dermatology Climate Change Task Force | INTERNATIONAL JOURNAL OF DERMATOLOGY | 10.1111/ijd.12941 | General climate-health, disaster, or other-disease study without dengue-specific analysis | The study concerned general climate-health, disasters, multiple diseases, or another disease; dengue appeared only as background, in a disease list, or as a secondary context without an identifiable dengue-specific analysis. |
| 146 | Moore | 2017 | El Nino and the shifting geography of cholera in Africa | PROCEEDINGS OF THE NATIONAL ACADEMY OF SCIENCES OF THE UNITED STATES OF AMERICA | 10.1073/pnas.1617218114 | Dengue mentioned only as background or an example | Dengue was mentioned only in the background, discussion, or as an example, without an identifiable dengue-specific analysis. |
| 147 | Heft | 2008 | Effects of the El Nino - Southern Oscillation (ENSO) cycle on mosquito populations in southern California | JOURNAL OF VECTOR ECOLOGY | 10.3376/1081-1710(2008)33[17:EOTENS]2.0.CO;2 | Vector-only study without a dengue-related outcome or context | The study focused on mosquito biology, ecology, abundance, distribution, or control without a substantive dengue-related outcome or analysis. |
| 148 | Gardner | 2015 | Risk of Dengue Spread from the Philippines Through International Air Travel | TRANSPORTATION RESEARCH RECORD | 10.3141/2501-04 | No relevant climate or meteorological exposure | The study addressed dengue but did not analyze a climatic, weather, meteorological, or extreme-weather exposure in relation to a dengue outcome. |
| 149 | Caminade | 2017 | Global risk model for vector-borne transmission of Zika virus reveals the role of El Nino 2015 | PROCEEDINGS OF THE NATIONAL ACADEMY OF SCIENCES OF THE UNITED STATES OF AMERICA | 10.1073/pnas.1614303114 | Vector-only study without a dengue-related outcome or context | The study focused on mosquito biology, ecology, abundance, distribution, or control without a substantive dengue-related outcome or analysis. |
| 150 | Morris | 2014 | Complex temporal climate signals drive the emergence of human water-borne disease | EMERGING MICROBES & INFECTIONS | 10.1038/emi.2014.56 | Not dengue-specific | Neither the title nor the abstract treated dengue as a study subject or reported a dengue-related outcome or analysis. |
| 151 | Rao | 2019 | Future increase in extreme El Nino events under greenhouse warming increases Zika virus incidence in South America | NPJ CLIMATE AND ATMOSPHERIC SCIENCE | 10.1038/s41612-019-0061-0 | Vector-only study without a dengue-related outcome or context | The study focused on mosquito biology, ecology, abundance, distribution, or control without a substantive dengue-related outcome or analysis. |
| 152 | Troncoso | 2016 | Zika threatens to become a huge worldwide pandemic | ASIAN PACIFIC JOURNAL OF TROPICAL BIOMEDICINE | 10.1016/j.apjtb.2016.04.004 | Dengue mentioned only as background or an example | The article discussed climate and health or another disease; dengue appeared only in the background or a disease list and was not analyzed separately. |
| 153 | Asad | 2018 | Effects of climate change on the spread of zika virus: a public health threat | REVIEWS ON ENVIRONMENTAL HEALTH | 10.1515/reveh-2017-0042 | Dengue mentioned only as background or an example | The article discussed climate and health or another disease; dengue appeared only in the background or a disease list and was not analyzed separately. |
| 154 | Iqbal | 2025 | Clinical characteristics and serotype association of dengue and dengue like illness in Pakistan | PLOS NEGLECTED TROPICAL DISEASES | 10.1371/journal.pntd.0012978 | Clinical, diagnostic, immunological, pathogenesis, or therapeutic dengue study | The study evaluated dengue clinical features, diagnosis, immune responses, molecular mechanisms, animal models, or treatment without using a climatic or meteorological factor as the exposure. |
| 155 | Roiz | 2015 | Autochthonous Chikungunya Transmission and Extreme Climate Events in Southern France | PLOS NEGLECTED TROPICAL DISEASES | 10.1371/journal.pntd.0003854 | Vector-only study without a dengue-related outcome or context | The study focused on mosquito biology, ecology, abundance, distribution, or control without a substantive dengue-related outcome or analysis. |
| 156 | Isarel Hernandez-Gaytan | 2017 | 20 Years Spatial-Temporal Analysis of Dengue Fever and Hemorrhagic Fever in Mexico | ARCHIVES OF MEDICAL RESEARCH | 10.1016/j.arcmed.2018.01.003 | No relevant climate or meteorological exposure | The study addressed dengue but did not analyze a climatic, weather, meteorological, or extreme-weather exposure in relation to a dengue outcome. |
| 157 | Kramer | 2026 | Heatwaves Constrain the Future Persistence of Mosquito Vectors in Europe | GLOBAL CHANGE BIOLOGY | 10.1111/gcb.70876 | Vector-only study without a dengue-related outcome or context | The study focused on mosquito biology, ecology, abundance, distribution, or control without a substantive dengue-related outcome or analysis. |
| 158 | Huang | 2025 | Impact of El Nino Southern Oscillation and Climate Change on Infectious Diseases with Ophthalmic Manifestations | TROPICAL MEDICINE AND INFECTIOUS DISEASE | 10.3390/tropicalmed10100297 | Dengue mentioned only as background or an example | The article discussed climate and health or another disease; dengue appeared only in the background or a disease list and was not analyzed separately. |
| 159 | Batchelor | 2012 | Influence of climate variability on human leptospirosis cases in Jamaica | CLIMATE RESEARCH | 10.3354/cr01120 | Not dengue-specific | Neither the title nor the abstract treated dengue as a study subject or reported a dengue-related outcome or analysis. |
| 160 | Peng | 2024 | The 2023 extreme coastal El Nino: Atmospheric and air-sea coupling mechanisms | SCIENCE ADVANCES | 10.1126/sciadv.adk8646 | General climate-health, disaster, or other-disease study without dengue-specific analysis | The study concerned general climate-health, disasters, multiple diseases, or another disease; dengue appeared only as background, in a disease list, or as a secondary context without an identifiable dengue-specific analysis. |
| 161 | Kreppel | 2014 | A Non-Stationary Relationship between Global Climate Phenomena and Human Plague Incidence in Madagascar | PLOS NEGLECTED TROPICAL DISEASES | 10.1371/journal.pntd.0003155 | Not dengue-specific | Neither the title nor the abstract treated dengue as a study subject or reported a dengue-related outcome or analysis. |
| 162 | Huang | 2019 | El Nino Southern Oscillation, overseas arrivals and imported chikungunya cases in Australia: A time series analysis | PLOS NEGLECTED TROPICAL DISEASES | 10.1371/journal.pntd.0007376 | Vector-only study without a dengue-related outcome or context | The study focused on mosquito biology, ecology, abundance, distribution, or control without a substantive dengue-related outcome or analysis. |
| 163 | Harapan | 2020 | Effects of El Nino Southern Oscillation and Dipole Mode Index on Chikungunya Infection in Indonesia | TROPICAL MEDICINE AND INFECTIOUS DISEASE | 10.3390/tropicalmed5030119 | Not dengue-specific | Neither the title nor the abstract treated dengue as a study subject or reported a dengue-related outcome or analysis. |
| 164 | Nagao | 2008 | Decreases in dengue transmission may act to increase the incidence of dengue hemorrhagic fever | PROCEEDINGS OF THE NATIONAL ACADEMY OF SCIENCES OF THE UNITED STATES OF AMERICA | 10.1073/pnas.0709029105 | No relevant climate or meteorological exposure | The study addressed dengue but did not analyze a climatic, weather, meteorological, or extreme-weather exposure in relation to a dengue outcome. |
| 165 | Espinoza | 2021 | Conflicting diagnostic and prognostic framing of epidemics? Newspaper representations of dengue as a public health problem in Peru | SOCIAL SCIENCE & MEDICINE | 10.1016/j.socscimed.2021.114398 | Clinical, diagnostic, immunological, pathogenesis, or therapeutic dengue study | The study evaluated dengue clinical features, diagnosis, immune responses, molecular mechanisms, animal models, or treatment without using a climatic or meteorological factor as the exposure. |
| 166 | Zhang | 2026 | Dengue Virus-Susceptible Animal Models: Research Progress, Core Bottlenecks, and Future Perspectives | VACCINES | 10.3390/vaccines14040319 | Clinical, diagnostic, immunological, pathogenesis, or therapeutic dengue study | The study evaluated dengue clinical features, diagnosis, immune responses, molecular mechanisms, animal models, or treatment without using a climatic or meteorological factor as the exposure. |
| 167 | Hamer | 2015 | Spatial Distribution of Epidemiological Cases of Dengue Fever in Suriname, 2001-2012 | WEST INDIAN MEDICAL JOURNAL | 10.7727/wimj.2014.118 | Dengue epidemiology, transmission, or social-factor study without climate-exposure analysis | The study focused on dengue outbreak description, spatial distribution, human mobility, public-health management, or social factors without directly analyzing a climate/meteorological exposure–dengue outcome association. |
| 168 | Martinez | 2024 | Cryptic transmission and novel introduction of Dengue 1 and 2 genotypes in Colombia | VIRUS EVOLUTION | 10.1093/ve/veae068 | Clinical, diagnostic, immunological, pathogenesis, or therapeutic dengue study | The study evaluated dengue clinical features, diagnosis, immune responses, molecular mechanisms, animal models, or treatment without using a climatic or meteorological factor as the exposure. |
| 169 | Moreno-Madrinan | 2017 | Factors of Concern Regarding Zika and Other Aedes aegypti-Transmitted Viruses in the United States | JOURNAL OF MEDICAL ENTOMOLOGY | 10.1093/jme/tjw212 | Vector-only study without a dengue-related outcome or context | The study focused on mosquito biology, ecology, abundance, distribution, or control without a substantive dengue-related outcome or analysis. |
| 170 | Suttitheptumrong | 2013 | Compound A, a dissociated glucocorticoid receptor modulator, reduces dengue virus-induced cytokine secretion and dengue virus production | BIOCHEMICAL AND BIOPHYSICAL RESEARCH COMMUNICATIONS | 10.1016/j.bbrc.2013.05.094 | Clinical, diagnostic, immunological, pathogenesis, or therapeutic dengue study | The study evaluated dengue clinical features, diagnosis, immune responses, molecular mechanisms, animal models, or treatment without using a climatic or meteorological factor as the exposure. |
| 171 | Komarudin | 2025 | Natural Compounds and Their Analogs as Antivirals Against Dengue Virus: A Review | PHYTOTHERAPY RESEARCH | 10.1002/ptr.8408 | Clinical, diagnostic, immunological, pathogenesis, or therapeutic dengue study | The study evaluated dengue clinical features, diagnosis, immune responses, molecular mechanisms, animal models, or treatment without using a climatic or meteorological factor as the exposure. |
| 172 | Phumesin | 2023 | Cepharanthine inhibits dengue virus production and cytokine secretion | VIRUS RESEARCH | 10.1016/j.virusres.2022.199030 | Clinical, diagnostic, immunological, pathogenesis, or therapeutic dengue study | The study evaluated dengue clinical features, diagnosis, immune responses, molecular mechanisms, animal models, or treatment without using a climatic or meteorological factor as the exposure. |
| 173 | Weaver | 2010 | Present and future arboviral threats | ANTIVIRAL RESEARCH | 10.1016/j.antiviral.2009.10.008 | Vector-only study without a dengue-related outcome or context | The measured outcomes concerned vector biology or ecology rather than dengue transmission, incidence, or disease risk. |
| 174 | Mustafa | 2024 | Distinct inflammatory markers in primary and secondary dengue infection: can cytokines CXCL5, CXCL9, and CCL17 act as surrogate markers? | PATHOGENS AND GLOBAL HEALTH | 10.1080/20477724.2024.2365581 | Clinical, diagnostic, immunological, pathogenesis, or therapeutic dengue study | The study evaluated dengue clinical features, diagnosis, immune responses, molecular mechanisms, animal models, or treatment without using a climatic or meteorological factor as the exposure. |
| 175 | Montenegro | 2024 | Wolbachia-based emerging strategies for control of vector-transmitted disease | ACTA TROPICA | 10.1016/j.actatropica.2024.107410 | Vector-control or intervention study without a climate–dengue association | The study evaluated Wolbachia, insecticides, vector control, health education, or mosquito habitats without directly estimating an association between climate/meteorological exposure and a dengue outcome. |
| 176 | Kakarla | 2019 | Temperature dependent transmission potential model for chikungunya in India | SCIENCE OF THE TOTAL ENVIRONMENT | 10.1016/j.scitotenv.2018.07.461 | Vector-only study without a dengue-related outcome or context | The study focused on mosquito biology, ecology, abundance, distribution, or control without a substantive dengue-related outcome or analysis. |
| 177 | Epelboin | 2017 | Zika virus: An updated review of competent or naturally infected mosquitoes | PLOS NEGLECTED TROPICAL DISEASES | 10.1371/journal.pntd.0005933 | Not dengue-specific | Neither the title nor the abstract treated dengue as a study subject or reported a dengue-related outcome or analysis. |
| 178 | Tsai | 2017 | Targeting heat shock factor 1 as an antiviral strategy against dengue virus replication in vitro and in vivo | ANTIVIRAL RESEARCH | 10.1016/j.antiviral.2017.07.008 | Temperature/heat term represented a technical or biomedical condition, not meteorological exposure | Temperature or heat referred to assay stability, sample preservation, experimental heat stress, or a molecular mechanism rather than environmental weather or climate exposure. |
| 179 | Chan | 2016 | Zika fever and congenital Zika syndrome: An unexpected emerging arboviral disease | JOURNAL OF INFECTION | 10.1016/j.jinf.2016.02.011 | Dengue mentioned only as background or an example | The article discussed climate and health or another disease; dengue appeared only in the background or a disease list and was not analyzed separately. |
| 180 | Lin | 2020 | Epidemiological characteristics of dengue in mainland China from 1990 to 2019 A descriptive analysis | MEDICINE | 10.1097/MD.0000000000021982 | Dengue epidemiology, transmission, or social-factor study without climate-exposure analysis | The study focused on dengue outbreak description, spatial distribution, human mobility, public-health management, or social factors without directly analyzing a climate/meteorological exposure–dengue outcome association. |
| 181 | Carlson | 2016 | An Ecological Assessment of the Pandemic Threat of Zika Virus | PLOS NEGLECTED TROPICAL DISEASES | 10.1371/journal.pntd.0004968 | General climate-health, disaster, or other-disease study without dengue-specific analysis | The study concerned general climate-health, disasters, multiple diseases, or another disease; dengue appeared only as background, in a disease list, or as a secondary context without an identifiable dengue-specific analysis. |
| 182 | Moreno-Legorreta | 2015 | DECADES OF EXPERIENCE IN THE DIAGNOSIS OF DENGUE FEVER IN THE NORTHWEST OF MEXICO | REVISTA DE INVESTIGACION CLINICA-CLINICAL AND TRANSLATIONAL INVESTIGATION |  | No relevant climate or meteorological exposure | The study addressed dengue but did not analyze a climatic, weather, meteorological, or extreme-weather exposure in relation to a dengue outcome. |
| 183 | Caldwell | 2021 | Climate predicts geographic and temporal variation in mosquito-borne disease dynamics on two continents | NATURE COMMUNICATIONS | 10.1038/s41467-021-21496-7 | Vector-only study without a dengue-related outcome or context | Dengue was mentioned only to describe the vector importance of the mosquito; the measured outcomes concerned mosquito biology, ecology, or control. |
| 184 | Yongpitakwattana | 2021 | Alpha-mangostin inhibits dengue virus production and pro-inflammatory cytokine/chemokine expression in dendritic cells | ARCHIVES OF VIROLOGY | 10.1007/s00705-021-05017-x | Clinical, diagnostic, immunological, pathogenesis, or therapeutic dengue study | The study evaluated dengue clinical features, diagnosis, immune responses, molecular mechanisms, animal models, or treatment without using a climatic or meteorological factor as the exposure. |
| 185 | Simon-Loriere | 2015 | High Anti-Dengue Virus Activity of the OAS Gene Family Is Associated With Increased Severity of Dengue | JOURNAL OF INFECTIOUS DISEASES | 10.1093/infdis/jiv321 | Clinical, diagnostic, immunological, pathogenesis, or therapeutic dengue study | The study evaluated dengue clinical features, diagnosis, immune responses, molecular mechanisms, animal models, or treatment without using a climatic or meteorological factor as the exposure. |
| 186 | Ogony | 2025 | Public health facility vulnerabilities, preparedness, and health outcomes for Plasmodium falciparum and dengue virus-infected children under 5 years with acute febrile illnesses in Western Kenya | FRONTIERS IN PUBLIC HEALTH | 10.3389/fpubh.2025.1526558 | Dengue epidemiology, transmission, or social-factor study without climate-exposure analysis | The study focused on dengue outbreak description, spatial distribution, human mobility, public-health management, or social factors without directly analyzing a climate/meteorological exposure–dengue outcome association. |
| 187 | Aune | 2022 | Extreme Precipitation Events and Infectious Disease Risk: A Scoping Review and Framework for Infectious Respiratory Viruses | INTERNATIONAL JOURNAL OF ENVIRONMENTAL RESEARCH AND PUBLIC HEALTH | 10.3390/ijerph19010165 | Not dengue-specific | Neither the title nor the abstract treated dengue as a study subject or reported a dengue-related outcome or analysis. |
| 188 | Yang | 2016 | Lower activation-induced T-cell apoptosis is related to the pathological immune response in secondary infection with hetero-serotype dengue virus | IMMUNOBIOLOGY | 10.1016/j.imbio.2015.11.009 | Clinical, diagnostic, immunological, pathogenesis, or therapeutic dengue study | The study evaluated dengue clinical features, diagnosis, immune responses, molecular mechanisms, animal models, or treatment without using a climatic or meteorological factor as the exposure. |
| 189 | Zeller | 2016 | Chikungunya: Its History in Africa and Asia and Its Spread to New Regions in 2013-2014 | JOURNAL OF INFECTIOUS DISEASES | 10.1093/infdis/jiw391 | Vector-only study without a dengue-related outcome or context | The study focused on mosquito biology, ecology, abundance, distribution, or control without a substantive dengue-related outcome or analysis. |
| 190 | Gu | 2022 | A wMel Wolbachia variant in Aedes aegypti from field-collected Drosophila melanogaster with increased phenotypic stability under heat stress | ENVIRONMENTAL MICROBIOLOGY | 10.1111/1462-2920.15966 | Vector-only study without a dengue-related outcome or context | Dengue was mentioned only to describe the vector importance of the mosquito; the measured outcomes concerned mosquito biology, ecology, or control. |
| 191 | Dexheimer Paploski | 2016 | Storm drains as larval development and adult resting sites for Aedes aegypti and Aedes albopictus in Salvador, Brazil | PARASITES & VECTORS | 10.1186/s13071-016-1705-0 | Vector-only study without a dengue-related outcome or context | Dengue was mentioned only to describe the vector importance of the mosquito; the measured outcomes concerned mosquito biology, ecology, or control. |
| 192 | Hassan | 2013 | Dengue Fever Outbreak in Lahore | JCPSP-JOURNAL OF THE COLLEGE OF PHYSICIANS AND SURGEONS PAKISTAN |  | Dengue epidemiology, transmission, or social-factor study without climate-exposure analysis | The study focused on dengue outbreak description, spatial distribution, human mobility, public-health management, or social factors without directly analyzing a climate/meteorological exposure–dengue outcome association. |
| 193 | Lertjuthaporn | 2021 | Kinetic Changes of Peripheral Blood Monocyte Subsets and Expression of Co-Stimulatory Molecules during Acute Dengue Virus Infection | PATHOGENS | 10.3390/pathogens10111458 | Dengue epidemiology, transmission, or social-factor study without climate-exposure analysis | The study focused on dengue outbreak description, spatial distribution, human mobility, public-health management, or social factors without directly analyzing a climate/meteorological exposure–dengue outcome association. |
| 194 | Liang | 2024 | The global incidence and trends of three common flavivirus infections (Dengue, yellow fever, and Zika) from 2011 to 2021 | FRONTIERS IN MICROBIOLOGY | 10.3389/fmicb.2024.1458166 | No relevant climate or meteorological exposure | The study addressed dengue but did not analyze a climatic, weather, meteorological, or extreme-weather exposure in relation to a dengue outcome. |
| 195 | Lorenz | 2026 | Brazil is already experiencing the brutal impacts of climate change | REVISTA DE SAUDE PUBLICA | 10.11606/s1518-8787.2026060007009 | Dengue mentioned only as background or an example | Dengue was mentioned only in the background, discussion, or as an example, without an identifiable dengue-specific analysis. |
| 196 | Nanda | 2021 | IL-18: The Forgotten Cytokine in Dengue Immunopathogenesis | JOURNAL OF IMMUNOLOGY RESEARCH | 10.1155/2021/8214656 | Clinical, diagnostic, immunological, pathogenesis, or therapeutic dengue study | The study evaluated dengue clinical features, diagnosis, immune responses, molecular mechanisms, animal models, or treatment without using a climatic or meteorological factor as the exposure. |
| 197 | Verma | 2022 | Clinicopathological alteration of symptoms with serotype among dengue infected pediatric patients | JOURNAL OF MEDICAL VIROLOGY | 10.1002/jmv.27862 | Clinical, diagnostic, immunological, pathogenesis, or therapeutic dengue study | The study evaluated dengue clinical features, diagnosis, immune responses, molecular mechanisms, animal models, or treatment without using a climatic or meteorological factor as the exposure. |
| 198 | Wang | 2025 | A novel immunocompetent transgenic mouse model of DHF reveals Syk-mediated Th2-polarized cytokine storm as a key driver of vascular leakage | EMERGING MICROBES & INFECTIONS | 10.1080/22221751.2025.2531178 | Clinical, diagnostic, immunological, pathogenesis, or therapeutic dengue study | The study evaluated dengue clinical features, diagnosis, immune responses, molecular mechanisms, animal models, or treatment without using a climatic or meteorological factor as the exposure. |
| 199 | Zaki | 2010 | Clinical manifestations of dengue and leptospirosis in children in Mumbai: an observational study | INFECTION | 10.1007/s15010-010-0030-3 | Clinical, diagnostic, immunological, pathogenesis, or therapeutic dengue study | The study evaluated dengue clinical features, diagnosis, immune responses, molecular mechanisms, animal models, or treatment without using a climatic or meteorological factor as the exposure. |
| 200 | Lai | 2020 | Roles of Macrophage Migration Inhibitory Factor in Dengue Pathogenesis: From Pathogenic Factor to Therapeutic Target | MICROORGANISMS | 10.3390/microorganisms8060891 | Vector-only study without a dengue-related outcome or context | The measured outcomes concerned vector biology or ecology rather than dengue transmission, incidence, or disease risk. |
| 201 | Rama Ravo | 2025 | Mechanistic Insights into Hepatic Pathogenesis of Dengue Virus Serotype-2: Host-Virus Interactions, Immune Dysregulation, and Therapeutic Perspectives | INTERNATIONAL JOURNAL OF MOLECULAR SCIENCES | 10.3390/ijms262210904 | Clinical, diagnostic, immunological, pathogenesis, or therapeutic dengue study | The study evaluated dengue clinical features, diagnosis, immune responses, molecular mechanisms, animal models, or treatment without using a climatic or meteorological factor as the exposure. |
| 202 | Ochoa-Sanchez | 2025 | Detection and attribution of climate change impacts in coupled natural-human systems in the Andes | COMMUNICATIONS EARTH & ENVIRONMENT | 10.1038/s43247-025-02092-9 | Not dengue-specific | Neither the title nor the abstract treated dengue as a study subject or reported a dengue-related outcome or analysis. |
| 203 | Nissan | 2021 | Climate-proofing a malaria eradication strategy | MALARIA JOURNAL | 10.1186/s12936-021-03718-x | Not dengue-specific | Neither the title nor the abstract treated dengue as a study subject or reported a dengue-related outcome or analysis. |
| 204 | Teixeira | 2022 | Suppression of TGF-β/Smad2 signaling by GW788388 enhances DENV-2 clearance in macrophages | JOURNAL OF MEDICAL VIROLOGY | 10.1002/jmv.27879 | Dengue epidemiology, transmission, or social-factor study without climate-exposure analysis | The study focused on dengue outbreak description, spatial distribution, human mobility, public-health management, or social factors without directly analyzing a climate/meteorological exposure–dengue outcome association. |
| 205 | Her | 2017 | Severity of Plasma Leakage Is Associated With High Levels of Interferon γ-Inducible Protein 10, Hepatocyte Growth Factor, Matrix Metalloproteinase 2 (MMP-2), and MMP-9 During Dengue Virus Infection | JOURNAL OF INFECTIOUS DISEASES | 10.1093/infdis/jiw494 | Clinical, diagnostic, immunological, pathogenesis, or therapeutic dengue study | The study evaluated dengue clinical features, diagnosis, immune responses, molecular mechanisms, animal models, or treatment without using a climatic or meteorological factor as the exposure. |
| 206 | Ali | 2022 | A Privacy-Preserved Internet-of-Medical-Things Scheme for Eradication and Control of Dengue Using UAV | MICROMACHINES | 10.3390/mi13101702 | Dengue epidemiology, transmission, or social-factor study without climate-exposure analysis | The study focused on dengue outbreak description, spatial distribution, human mobility, public-health management, or social factors without directly analyzing a climate/meteorological exposure–dengue outcome association. |
| 207 | Vasquez | 2023 | wMel replacement of dengue-competent mosquitoes is robust to near-term change | NATURE CLIMATE CHANGE | 10.1038/s41558-023-01746-w | Vector-only study without a dengue-related outcome or context | The measured outcomes concerned vector biology or ecology rather than dengue transmission, incidence, or disease risk. |
| 208 | Arora | 2018 | Zika outbreak aftermath: status, progress, concerns and new insights | FUTURE VIROLOGY | 10.2217/fvl-2018-0040 | Not dengue-specific | Neither the title nor the abstract treated dengue as a study subject or reported a dengue-related outcome or analysis. |
| 209 | Zell | 2008 | Impact of global warming on viral diseases: what is the evidence? | CURRENT OPINION IN BIOTECHNOLOGY | 10.1016/j.copbio.2008.10.009 | Not dengue-specific | Neither the title nor the abstract treated dengue as a study subject or reported a dengue-related outcome or analysis. |
| 210 | Rothman | 2011 | Immunity to dengue virus: a tale of original antigenic sin and tropical cytokine storms | NATURE REVIEWS IMMUNOLOGY | 10.1038/nri3014 | Clinical, diagnostic, immunological, pathogenesis, or therapeutic dengue study | The study evaluated dengue clinical features, diagnosis, immune responses, molecular mechanisms, animal models, or treatment without using a climatic or meteorological factor as the exposure. |
| 211 | Kurane | 2009 | The Emerging and Forecasted Effect of Climate Change on Human Health | JOURNAL OF HEALTH SCIENCE | 10.1248/jhs.55.865 | Vector-only study without a dengue-related outcome or context | The study focused on mosquito biology, ecology, abundance, distribution, or control without a substantive dengue-related outcome or analysis. |
| 212 | Melebari | 2021 | The epidemiology and incidence of dengue in Makkah, Saudi Arabia, during 2017-2019 | SAUDI MEDICAL JOURNAL | 10.15537/smj.2021.42.11.20210124 | Dengue epidemiology, transmission, or social-factor study without climate-exposure analysis | The study focused on dengue outbreak description, spatial distribution, human mobility, public-health management, or social factors without directly analyzing a climate/meteorological exposure–dengue outcome association. |
| 213 | Lee | 2024 | Inhibitory peptides derived from Hepatitis C virus NS5A for reducing clinical symptoms of dengue virus infection | ANTIVIRAL RESEARCH | 10.1016/j.antiviral.2024.106018 | Clinical, diagnostic, immunological, pathogenesis, or therapeutic dengue study | The study evaluated dengue clinical features, diagnosis, immune responses, molecular mechanisms, animal models, or treatment without using a climatic or meteorological factor as the exposure. |
| 214 | Rattanaburee | 2015 | Inhibition of dengue virus production and cytokine/chemokine expression by ribavirin and compound A | ANTIVIRAL RESEARCH | 10.1016/j.antiviral.2015.10.005 | Clinical, diagnostic, immunological, pathogenesis, or therapeutic dengue study | The study evaluated dengue clinical features, diagnosis, immune responses, molecular mechanisms, animal models, or treatment without using a climatic or meteorological factor as the exposure. |
| 215 | Ortiz-Prado | 2024 | Urgent Response Needed: Addressing the Dengue Crisis in the Andean and Southern Cone Latin American Regions | AMERICAN JOURNAL OF TROPICAL MEDICINE AND HYGIENE | 10.4269/ajtmh.24-0178 | Dengue epidemiology, transmission, or social-factor study without climate-exposure analysis | The study focused on dengue outbreak description, spatial distribution, human mobility, public-health management, or social factors without directly analyzing a climate/meteorological exposure–dengue outcome association. |
| 216 | Sumaye | 2019 | Rift Valley fever: An open-source transmission dynamics simulation model | PLOS ONE | 10.1371/journal.pone.0209929 | Not dengue-specific | Neither the title nor the abstract treated dengue as a study subject or reported a dengue-related outcome or analysis. |
| 217 | Valle | 2013 | Improving the Modeling of Disease Data from the Government Surveillance System: A Case Study on Malaria in the Brazilian Amazon | PLOS COMPUTATIONAL BIOLOGY | 10.1371/journal.pcbi.1003312 | Not dengue-specific | Neither the title nor the abstract treated dengue as a study subject or reported a dengue-related outcome or analysis. |
| 218 | Panya | 2021 | Triphala in Traditional Ayurvedic Medicine Inhibits Dengue Virus Infection in Huh7 Hepatoma Cells | PHARMACEUTICALS | 10.3390/ph14121236 | Clinical, diagnostic, immunological, pathogenesis, or therapeutic dengue study | The study evaluated dengue clinical features, diagnosis, immune responses, molecular mechanisms, animal models, or treatment without using a climatic or meteorological factor as the exposure. |
| 219 | Deng | 2025 | Fosaprepitant Dimeglumine Alleviates Dengue Virus Infection and Virus-Induced Inflammatory Responses | ACS INFECTIOUS DISEASES | 10.1021/acsinfecdis.5c00490 | Dengue epidemiology, transmission, or social-factor study without climate-exposure analysis | The study focused on dengue outbreak description, spatial distribution, human mobility, public-health management, or social factors without directly analyzing a climate/meteorological exposure–dengue outcome association. |
| 220 | Bonds | 2012 | Ultra-low-volume space sprays in mosquito control: a critical review | MEDICAL AND VETERINARY ENTOMOLOGY | 10.1111/j.1365-2915.2011.00992.x | Vector-only study without a dengue-related outcome or context | The study focused on mosquito biology, ecology, abundance, distribution, or control without a substantive dengue-related outcome or analysis. |
| 221 | Lim | 2021 | Recent insights on anti-dengue virus (DENV) medicinal plants: review on in vitro, in vivo and in silico discoveries | ALL LIFE | 10.1080/26895293.2020.1856192 | Clinical, diagnostic, immunological, pathogenesis, or therapeutic dengue study | The study evaluated dengue clinical features, diagnosis, immune responses, molecular mechanisms, animal models, or treatment without using a climatic or meteorological factor as the exposure. |
| 222 | Poveda | 2020 | Concomitant malaria, dengue and COVID-19: extraordinary challenge for Colombia's public health system | CURRENT OPINION IN ENVIRONMENTAL SUSTAINABILITY |  | Dengue epidemiology, transmission, or social-factor study without climate-exposure analysis | The study focused on dengue outbreak description, spatial distribution, human mobility, public-health management, or social factors without directly analyzing a climate/meteorological exposure–dengue outcome association. |
| 223 | Foo | 2019 | Cross-Generational Effects of Heat Stress on Fitness and Wolbachia Density in Aedes aegypti Mosquitoes | TROPICAL MEDICINE AND INFECTIOUS DISEASE | 10.3390/tropicalmed4010013 | Vector-only study without a dengue-related outcome or context | The study focused on mosquito biology, ecology, abundance, distribution, or control without a substantive dengue-related outcome or analysis. |
| 224 | Manrique-Saide | 2012 | STORM SEWERS AS LARVAL HABITATS FOR AEDES AEGYPTI AND CULEX SPP. IN A NEIGHBORHOOD OF MERIDA, MEXICO | JOURNAL OF THE AMERICAN MOSQUITO CONTROL ASSOCIATION | 10.2987/12-6244R.1 | Vector-control or intervention study without a climate–dengue association | The study evaluated Wolbachia, insecticides, vector control, health education, or mosquito habitats without directly estimating an association between climate/meteorological exposure and a dengue outcome. |
| 225 | Satria | 2022 | Elevated TNF-α Induces Thrombophagocytosis by Mononuclear Cells in ex vivo Whole-Blood Co-Culture with Dengue Virus | JOURNAL OF INFLAMMATION RESEARCH | 10.2147/JIR.S356742 | No relevant climate or meteorological exposure | The study addressed dengue but did not analyze a climatic, weather, meteorological, or extreme-weather exposure in relation to a dengue outcome. |
| 226 | Jacups | 2014 | Residual and pre- treatment application of starycide insect growth regulator ( triflumuron) to control Aedes aegypti in containers | PEST MANAGEMENT SCIENCE | 10.1002/ps.3576 | Vector-only study without a dengue-related outcome or context | Dengue was mentioned only to describe the vector importance of the mosquito; the measured outcomes concerned mosquito biology, ecology, or control. |
| 227 | Valero | 2019 | Increased serum ferritin and interleukin-18 levels in children with dengue | BRAZILIAN JOURNAL OF MICROBIOLOGY | 10.1007/s42770-019-00105-2 | Dengue epidemiology, transmission, or social-factor study without climate-exposure analysis | The study focused on dengue outbreak description, spatial distribution, human mobility, public-health management, or social factors without directly analyzing a climate/meteorological exposure–dengue outcome association. |
| 228 | Gandini | 2013 | Dengue Virus Activates Membrane TRAIL Relocalization and IFN-α Production by Human Plasmacytoid Dendritic Cells In Vitro and In Vivo | PLOS NEGLECTED TROPICAL DISEASES | 10.1371/journal.pntd.0002257 | Clinical, diagnostic, immunological, pathogenesis, or therapeutic dengue study | The study evaluated dengue clinical features, diagnosis, immune responses, molecular mechanisms, animal models, or treatment without using a climatic or meteorological factor as the exposure. |
| 229 | Weller | 2014 | Seroprevalence of Alphavirus Antibodies in a Cross-Sectional Study in Southwestern Tanzania Suggests Endemic Circulation of Chikungunya | PLOS NEGLECTED TROPICAL DISEASES | 10.1371/journal.pntd.0002979 | Vector-only study without a dengue-related outcome or context | The study focused on mosquito biology, ecology, abundance, distribution, or control without a substantive dengue-related outcome or analysis. |
| 230 | Costa | 2022 | Targeting the Annexin A1-FPR2/ALX pathway for host-directed therapy in dengue disease | ELIFE | 10.7554/eLife.73853 | Clinical, diagnostic, immunological, pathogenesis, or therapeutic dengue study | The study evaluated dengue clinical features, diagnosis, immune responses, molecular mechanisms, animal models, or treatment without using a climatic or meteorological factor as the exposure. |
| 231 | Zhang | 2025 | The Role of Emerging/Re-Emerging RNA Viruses in Bone-Related Diseases With a Focus on DENV, CHIKV, and SARS-CoV-2 | REVIEWS IN MEDICAL VIROLOGY | 10.1002/rmv.70053 | Dengue epidemiology, transmission, or social-factor study without climate-exposure analysis | The study focused on dengue outbreak description, spatial distribution, human mobility, public-health management, or social factors without directly analyzing a climate/meteorological exposure–dengue outcome association. |
| 232 | Tarasuk | 2022 | Alpha-mangostin inhibits viral replication and suppresses nuclear factor kappa B (NF-κB)-mediated inflammation in dengue virus infection | SCIENTIFIC REPORTS | 10.1038/s41598-022-20284-7 | Clinical, diagnostic, immunological, pathogenesis, or therapeutic dengue study | The study evaluated dengue clinical features, diagnosis, immune responses, molecular mechanisms, animal models, or treatment without using a climatic or meteorological factor as the exposure. |
| 233 | Lopez-Zeron | 2024 | INSECTICIDE SUSCEPTIBILITY IN TWO POPULATIONS OF Aedes aegypti L. (Diptera: Culicidae) FROM GUERRERO, MEXICO, AND RECOMMENDATIONS FOR THEIR MANAGEMENT | AGROCIENCIA | 10.47163/agrociencia.v58i7.3041 | Vector-only study without a dengue-related outcome or context | Dengue was mentioned only to describe the vector importance of the mosquito; the measured outcomes concerned mosquito biology, ecology, or control. |
| 234 | Amin | 2025 | Dengue virus infection: how platelet-leukocyte crosstalk shapes thrombotic events and inflammation | MOLECULAR BIOLOGY REPORTS | 10.1007/s11033-025-10222-x | Clinical, diagnostic, immunological, pathogenesis, or therapeutic dengue study | The study evaluated dengue clinical features, diagnosis, immune responses, molecular mechanisms, animal models, or treatment without using a climatic or meteorological factor as the exposure. |
| 235 | Trajer | 2014 | Seasonality and geographical occurrence of West Nile fever and distribution of Asian tiger mosquito | IDOJARAS |  | Vector-only study without a dengue-related outcome or context | The study focused on mosquito biology, ecology, abundance, distribution, or control without a substantive dengue-related outcome or analysis. |
| 236 | Parker | 2023 | Determinants of exposure to Aedes mosquitoes: A comprehensive geospatial analysis in peri-urban Cambodia | ACTA TROPICA | 10.1016/j.actatropica.2023.106829 | Vector-only study without a dengue-related outcome or context | Dengue was mentioned only to describe the vector importance of the mosquito; the measured outcomes concerned mosquito biology, ecology, or control. |
| 237 | Yenew | 2025 | Scoping review on assessing climate-sensitive health risks | BMC PUBLIC HEALTH | 10.1186/s12889-025-22148-x | Dengue mentioned only as background or an example | Dengue was mentioned only in the background, discussion, or as an example, without an identifiable dengue-specific analysis. |
| 238 | Tarasuk | 2024 | Dual action effects of ethyl-p-methoxycinnamate against dengue virus infection and inflammation via NF-κB pathway suppression | SCIENTIFIC REPORTS | 10.1038/s41598-024-60070-1 | Dengue epidemiology, transmission, or social-factor study without climate-exposure analysis | The study focused on dengue outbreak description, spatial distribution, human mobility, public-health management, or social factors without directly analyzing a climate/meteorological exposure–dengue outcome association. |
| 239 | Desjardins | 2026 | Utilizing perceptions of public health officials in Cali, Colombia to support the surveillance of co-circulating vector-borne diseases | SOCIAL SCIENCE & MEDICINE | 10.1016/j.socscimed.2025.118890 | Dengue mentioned only as background or an example | The article discussed climate and health or another disease; dengue appeared only in the background or a disease list and was not analyzed separately. |
| 240 | van de Weg | 2013 | Microbial Translocation Is Associated with Extensive Immune Activation in Dengue Virus Infected Patients with Severe Disease | PLOS NEGLECTED TROPICAL DISEASES | 10.1371/journal.pntd.0002236 | Clinical, diagnostic, immunological, pathogenesis, or therapeutic dengue study | The study evaluated dengue clinical features, diagnosis, immune responses, molecular mechanisms, animal models, or treatment without using a climatic or meteorological factor as the exposure. |
| 241 | Kouadio | 2012 | Infectious diseases following natural disasters: prevention and control measures | EXPERT REVIEW OF ANTI-INFECTIVE THERAPY | 10.1586/ERI.11.155 | General climate-health, disaster, or other-disease study without dengue-specific analysis | The study concerned general climate-health, disasters, multiple diseases, or another disease; dengue appeared only as background, in a disease list, or as a secondary context without an identifiable dengue-specific analysis. |
| 242 | Sarathy | 2015 | Mouse models of dengue virus infection for vaccine testing | VACCINE | 10.1016/j.vaccine.2015.09.112 | Vector-only study without a dengue-related outcome or context | The measured outcomes concerned vector biology or ecology rather than dengue transmission, incidence, or disease risk. |
| 243 | Bernardes Terzian | 2017 | Viral Load and Cytokine Response Profile Does Not Support Antibody-Dependent Enhancement in Dengue-Primed Zika Virus-Infected Patients | CLINICAL INFECTIOUS DISEASES | 10.1093/cid/cix558 | No relevant climate or meteorological exposure | The study addressed dengue but did not analyze a climatic, weather, meteorological, or extreme-weather exposure in relation to a dengue outcome. |
| 244 | Grossi-Soyster | 2017 | Serological and spatial analysis of alphavirus and flavivirus prevalence and risk factors in a rural community in western Kenya | PLOS NEGLECTED TROPICAL DISEASES | 10.1371/journal.pntd.0005998 | Dengue mentioned only as background or an example | Dengue was mentioned only in the background, discussion, or as an example, without an identifiable dengue-specific analysis. |
| 245 | Martins | 2026 | Cyclodextrin-based delivery of Annexin A1 mimetic Ac2-26 enhances anti-inflammatory effect and prevents dengue-induced lethality combined with antiviral therapy | BRITISH JOURNAL OF PHARMACOLOGY | 10.1111/bph.70303 | Clinical, diagnostic, immunological, pathogenesis, or therapeutic dengue study | The study evaluated dengue clinical features, diagnosis, immune responses, molecular mechanisms, animal models, or treatment without using a climatic or meteorological factor as the exposure. |
| 246 | Knoblauch | 2024 | High-resolution mapping of urban Aedes aegypti immature abundance through breeding site detection based on satellite and street view imagery | SCIENTIFIC REPORTS | 10.1038/s41598-024-67914-w | Vector-only study without a dengue-related outcome or context | Dengue was mentioned only to describe the vector importance of the mosquito; the measured outcomes concerned mosquito biology, ecology, or control. |
| 247 | Hernandez | 2025 | Leptospirosis? An epidemiologic investigation following the historic 2024 floods in Rio Grande do Sul, Brazil | ONE HEALTH | 10.1016/j.onehlt.2025.101146 | General climate-health, disaster, or other-disease study without dengue-specific analysis | The study concerned general climate-health, disasters, multiple diseases, or another disease; dengue appeared only as background, in a disease list, or as a secondary context without an identifiable dengue-specific analysis. |
| 248 | Filippelli | 2020 | Climate change impacts on human health at an actionable scale: a state-level assessment of Indiana, USA | CLIMATIC CHANGE | 10.1007/s10584-020-02710-9 | Not dengue-specific | Neither the title nor the abstract treated dengue as a study subject or reported a dengue-related outcome or analysis. |
| 249 | Le Tong | 2025 | Climate Change and the Impact on Ocular Infectious Diseases: A Narrative Review | OPHTHALMOLOGY AND THERAPY | 10.1007/s40123-025-01185-0 | General climate-health, disaster, or other-disease study without dengue-specific analysis | The study concerned general climate-health, disasters, multiple diseases, or another disease; dengue appeared only as background, in a disease list, or as a secondary context without an identifiable dengue-specific analysis. |
| 250 | Beebe | 2013 | Tracing the Tiger: Population Genetics Provides Valuable Insights into the Aedes (Stegomyia) albopictus Invasion of the Australasian Region | PLOS NEGLECTED TROPICAL DISEASES | 10.1371/journal.pntd.0002361 | Dengue mentioned only as background or an example | Dengue was mentioned only in the background, discussion, or as an example, without an identifiable dengue-specific analysis. |
| 251 | Raekiansyah | 2014 | Dengue Virus neither Directly Mediates Hyperpermeability nor Enhances Tumor Necrosis Factor-α-Induced Permeability In Vitro | JAPANESE JOURNAL OF INFECTIOUS DISEASES | 10.7883/yoken.67.86 | Clinical, diagnostic, immunological, pathogenesis, or therapeutic dengue study | The study evaluated dengue clinical features, diagnosis, immune responses, molecular mechanisms, animal models, or treatment without using a climatic or meteorological factor as the exposure. |
| 252 | Kuczera | 2018 | Highlights for Dengue Immunopathogenesis: Antibody-Dependent Enhancement, Cytokine Storm, and Beyond | JOURNAL OF INTERFERON AND CYTOKINE RESEARCH | 10.1089/jir.2017.0037 | Clinical, diagnostic, immunological, pathogenesis, or therapeutic dengue study | The study evaluated dengue clinical features, diagnosis, immune responses, molecular mechanisms, animal models, or treatment without using a climatic or meteorological factor as the exposure. |
| 253 | Zhang | 2023 | Environmental emissions and pollution characteristics of mosquitocides for the control of dengue fever in a typical urban area | SCIENCE OF THE TOTAL ENVIRONMENT | 10.1016/j.scitotenv.2023.161513 | Vector-control or intervention study without a climate–dengue association | The study evaluated Wolbachia, insecticides, vector control, health education, or mosquito habitats without directly estimating an association between climate/meteorological exposure and a dengue outcome. |
| 254 | How | 2023 | Dengue vigilance in COVID-19 pandemic | KUWAIT MEDICAL JOURNAL |  | No relevant climate or meteorological exposure | The study addressed dengue but did not analyze a climatic, weather, meteorological, or extreme-weather exposure in relation to a dengue outcome. |
| 255 | Wang | 2025 | Long-Term Stabilization of Dengue Virus RNA at 37 °C for 14 Months Using Silk Fibroin Films | VIRUSES-BASEL | 10.3390/v17111452 | Temperature/heat term represented a technical or biomedical condition, not meteorological exposure | Temperature or heat referred to assay stability, sample preservation, experimental heat stress, or a molecular mechanism rather than environmental weather or climate exposure. |
| 256 | Ahmed | 2020 | Climate Change, Water Quality and Water-Related Challenges: A Review with Focus on Pakistan | INTERNATIONAL JOURNAL OF ENVIRONMENTAL RESEARCH AND PUBLIC HEALTH | 10.3390/ijerph17228518 | General climate-health, disaster, or other-disease study without dengue-specific analysis | The study concerned general climate-health, disasters, multiple diseases, or another disease; dengue appeared only as background, in a disease list, or as a secondary context without an identifiable dengue-specific analysis. |
| 257 | Ciota | 2013 | Vector-Virus Interactions and Transmission Dynamics of West Nile Virus | VIRUSES-BASEL | 10.3390/v5123021 | Vector-only study without a dengue-related outcome or context | The study focused on mosquito biology, ecology, abundance, distribution, or control without a substantive dengue-related outcome or analysis. |
| 258 | Povoa | 2016 | Peripheral Organs of Dengue Fatal Cases Present Strong Pro-Inflammatory Response with Participation of IFN-Gamma-, TNF-Alpha- and RANTES-Producing Cells | PLOS ONE | 10.1371/journal.pone.0168973 | Dengue epidemiology, transmission, or social-factor study without climate-exposure analysis | The study focused on dengue outbreak description, spatial distribution, human mobility, public-health management, or social factors without directly analyzing a climate/meteorological exposure–dengue outcome association. |
| 259 | Ocampo | 2014 | Reduction in dengue cases observed during mass control of Aedes (Stegomyia) in street catch basins in an endemic urban area in Colombia | ACTA TROPICA | 10.1016/j.actatropica.2013.12.019 | Vector-control or intervention study without a climate–dengue association | The study evaluated Wolbachia, insecticides, vector control, health education, or mosquito habitats without directly estimating an association between climate/meteorological exposure and a dengue outcome. |
| 260 | Teillet | 2025 | Characterizing urban landscapes using very-high resolution satellite imagery to predict Ae. albopictus larval presence probability in public spaces | PLOS ONE | 10.1371/journal.pone.0335794 | Vector-control or intervention study without a climate–dengue association | The study evaluated Wolbachia, insecticides, vector control, health education, or mosquito habitats without directly estimating an association between climate/meteorological exposure and a dengue outcome. |
| 261 | Fulcher | 2015 | EVALUATION OF A NEW SPRAYING MACHINE FOR BARRIER TREATMENT AND PENETRATION OF BIFENTHRIN ON VEGETATION AGAINST MOSQUITOES | JOURNAL OF THE AMERICAN MOSQUITO CONTROL ASSOCIATION | 10.2987/14-6424R.1 | Vector-only study without a dengue-related outcome or context | The study focused on mosquito biology, ecology, abundance, distribution, or control without a substantive dengue-related outcome or analysis. |
| 262 | Gunasekaran | 2022 | Sensitivity of wMel and wAlbB Wolbachia infections in Aedes aegypti Puducherry (Indian) strains to heat stress during larval development | PARASITES & VECTORS | 10.1186/s13071-022-05345-0 | Vector-only study without a dengue-related outcome or context | Dengue was mentioned only to describe the vector importance of the mosquito; the measured outcomes concerned mosquito biology, ecology, or control. |
| 263 | Alcayna | 2025 | Identifying the climate sensitivity of infectious diseases: a conceptual framework | LANCET PLANETARY HEALTH | 10.1016/j.lanplh.2025.101291 | Not dengue-specific | Neither the title nor the abstract treated dengue as a study subject or reported a dengue-related outcome or analysis. |
| 264 | Nanaware | 2021 | Dengue Virus Infection: A Tale of Viral Exploitations and Host Responses | VIRUSES-BASEL | 10.3390/v13101967 | No relevant climate or meteorological exposure | The study addressed dengue but did not analyze a climatic, weather, meteorological, or extreme-weather exposure in relation to a dengue outcome. |
| 265 | Chen | 2025 | Impact of climate change and extreme temperature on the incidence of infectious disease among children and adolescents in China: A nationwide case-crossover study with over 8.7 million cases between 2008 and 2019 | JOURNAL OF INFECTION | 10.1016/j.jinf.2025.106547 | Dengue mentioned only as background or an example | Dengue was mentioned only in the background, discussion, or as an example, without an identifiable dengue-specific analysis. |
| 266 | Wang | 2024 | Progress and challenges in development of animal models for dengue virus infection | EMERGING MICROBES & INFECTIONS | 10.1080/22221751.2024.2404159 | Clinical, diagnostic, immunological, pathogenesis, or therapeutic dengue study | The study evaluated dengue clinical features, diagnosis, immune responses, molecular mechanisms, animal models, or treatment without using a climatic or meteorological factor as the exposure. |
| 267 | Holmes | 2023 | Dehydration Alters Transcript Levels in the Mosquito Midgut, Likely Facilitating Rapid Rehydration following a Bloodmeal | INSECTS | 10.3390/insects14030274 | Vector-only study without a dengue-related outcome or context | Dengue was mentioned only to describe the vector importance of the mosquito; the measured outcomes concerned mosquito biology, ecology, or control. |
| 268 | Su | 2026 | Unveiling the insights of controlling invasive Aedes mosquitoes in simulated urban catch basins by Sumilarv 0.5G Sachet containing pyriproxyfen | JOURNAL OF MEDICAL ENTOMOLOGY | 10.1093/jme/tjag015 | Vector-only study without a dengue-related outcome or context | The study focused on mosquito biology, ecology, abundance, distribution, or control without a substantive dengue-related outcome or analysis. |
| 269 | Nizhu | 2025 | Therapeutic plasma exchange in expanded dengue patients having acute liver failure: A case series | TROPICAL DOCTOR | 10.1177/00494755241286564 | Clinical, diagnostic, immunological, pathogenesis, or therapeutic dengue study | The study evaluated dengue clinical features, diagnosis, immune responses, molecular mechanisms, animal models, or treatment without using a climatic or meteorological factor as the exposure. |
| 270 | Khan | 2024 | From mosquitoes to menace: Unveiling the dangers of West Nile virus in Pakistan | TROPICAL DOCTOR | 10.1177/00494755231224095 | Not dengue-specific | Neither the title nor the abstract treated dengue as a study subject or reported a dengue-related outcome or analysis. |
| 271 | Roosa | 2022 | A general modeling framework for exploring the impact of individual concern and personal protection on vector-borne disease dynamics | PARASITES & VECTORS | 10.1186/s13071-022-05481-7 | Vector-only study without a dengue-related outcome or context | The study focused on mosquito biology, ecology, abundance, distribution, or control without a substantive dengue-related outcome or analysis. |
| 272 | Patra | 2021 | The relationship between changes in IL-2/IL-18 and liver enzyme with dengue severity | CYTOKINE | 10.1016/j.cyto.2021.155713 | Clinical, diagnostic, immunological, pathogenesis, or therapeutic dengue study | The study evaluated dengue clinical features, diagnosis, immune responses, molecular mechanisms, animal models, or treatment without using a climatic or meteorological factor as the exposure. |
| 273 | Ritchie | 2015 | Application of wMelPop Wolbachia Strain to Crash Local Populations of Aedes aegypti | PLOS NEGLECTED TROPICAL DISEASES | 10.1371/journal.pntd.0003930 | Vector-only study without a dengue-related outcome or context | Dengue was mentioned only to describe the vector importance of the mosquito; the measured outcomes concerned mosquito biology, ecology, or control. |
| 274 | Singh | 2024 | Anti-dengue therapeutic potential of Tinospora cordifolia and its bioactives | JOURNAL OF ETHNOPHARMACOLOGY | 10.1016/j.jep.2024.118242 | Vector-only study without a dengue-related outcome or context | The measured outcomes concerned vector biology or ecology rather than dengue transmission, incidence, or disease risk. |
| 275 | Perez-Guerra | 2023 | Community perceptions on challenges and solutions to implement an Aedes aegypti control project in Ponce, Puerto Rico (USA) | PLOS ONE | 10.1371/journal.pone.0284430 | Vector-only study without a dengue-related outcome or context | The study focused on mosquito biology, ecology, abundance, distribution, or control without a substantive dengue-related outcome or analysis. |
| 276 | Aik | 2023 | Climate change and population health in Singapore: a systematic review | LANCET REGIONAL HEALTH-WESTERN PACIFIC | 10.1016/j.lanwpc.2023.100947 | Dengue mentioned only as background or an example | Dengue was mentioned only in the background, discussion, or as an example, without an identifiable dengue-specific analysis. |
| 277 | Hsieh | 2017 | Ascertaining the impact of catastrophic events on dengue outbreak: The 2014 gas explosions in Kaohsiung, Taiwan | PLOS ONE | 10.1371/journal.pone.0177422 | Dengue epidemiology, transmission, or social-factor study without climate-exposure analysis | The study focused on dengue outbreak description, spatial distribution, human mobility, public-health management, or social factors without directly analyzing a climate/meteorological exposure–dengue outcome association. |
| 278 | Runde | 2022 | WIDE-AREA LARVICIDING WITH A BUFFALO TURBINE® MIST SPRAYER AND VECTOLEX® WDG | JOURNAL OF THE AMERICAN MOSQUITO CONTROL ASSOCIATION |  | Vector-only study without a dengue-related outcome or context | The study focused on mosquito biology, ecology, abundance, distribution, or control without a substantive dengue-related outcome or analysis. |
| 279 | Hubalek | 2008 | Mosquito-borne viruses in Europe | PARASITOLOGY RESEARCH | 10.1007/s00436-008-1064-7 | Vector-only study without a dengue-related outcome or context | Dengue was mentioned only to describe the vector importance of the mosquito; the measured outcomes concerned mosquito biology, ecology, or control. |
| 280 | Tsakni | 2025 | In Vitro Determination of Antimicrobial, Antioxidant and Antiviral Properties of Greek Plant Extracts | MICROORGANISMS | 10.3390/microorganisms13010177 | No relevant climate or meteorological exposure | The study addressed dengue but did not analyze a climatic, weather, meteorological, or extreme-weather exposure in relation to a dengue outcome. |
| 281 | Segala | 2025 | Insights into the ecological and climate crisis: Emerging infections threatening human health | ACTA TROPICA | 10.1016/j.actatropica.2025.107531 | Dengue mentioned only as background or an example | Dengue was mentioned only in the background, discussion, or as an example, without an identifiable dengue-specific analysis. |
| 282 | Nelder | 2010 | Suppressing Aedes albopictus, an Emerging Vector of Dengue and Chikungunya Viruses, by a Novel Combination of a Monomolecular Film and an Insect-Growth Regulator | AMERICAN JOURNAL OF TROPICAL MEDICINE AND HYGIENE | 10.4269/ajtmh.2010.09-0546 | Vector-only study without a dengue-related outcome or context | The measured outcomes concerned vector biology or ecology rather than dengue transmission, incidence, or disease risk. |
| 283 | Ahmed | 2021 | The Emergence of Rift Valley Fever in Gedaref State Urges the Need for a Cross-Border One Health Strategy and Enforcement of the International Health Regulations | PATHOGENS | 10.3390/pathogens10070885 | Dengue mentioned only as background or an example | The article discussed climate and health or another disease; dengue appeared only in the background or a disease list and was not analyzed separately. |
| 284 | Guabiraba | 2010 | Role of the Chemokine Receptors CCR1, CCR2 and CCR4 in the Pathogenesis of Experimental Dengue Infection in Mice | PLOS ONE | 10.1371/journal.pone.0015680 | Clinical, diagnostic, immunological, pathogenesis, or therapeutic dengue study | The study evaluated dengue clinical features, diagnosis, immune responses, molecular mechanisms, animal models, or treatment without using a climatic or meteorological factor as the exposure. |
| 285 | Garcia | 2024 | Exploring the Contrasts and Similarities of Dengue and SARS-CoV-2 Infections During the COVID-19 Era | INTERNATIONAL JOURNAL OF MOLECULAR SCIENCES | 10.3390/ijms252111624 | Dengue epidemiology, transmission, or social-factor study without climate-exposure analysis | The study focused on dengue outbreak description, spatial distribution, human mobility, public-health management, or social factors without directly analyzing a climate/meteorological exposure–dengue outcome association. |
| 286 | Klobucar | 2016 | An overview of mosquitoes and emerging arboviral infections in the Zagreb area, Croatia | JOURNAL OF INFECTION IN DEVELOPING COUNTRIES | 10.3855/jidc.7988 | Vector-only study without a dengue-related outcome or context | The study focused on mosquito biology, ecology, abundance, distribution, or control without a substantive dengue-related outcome or analysis. |
| 287 | Chang | 2016 | Characterizing Hospital Admissions to a Tertiary Care Hospital After Typhoon Haiyan | DISASTER MEDICINE AND PUBLIC HEALTH PREPAREDNESS | 10.1017/dmp.2015.165 | No relevant climate or meteorological exposure | The study addressed dengue but did not analyze a climatic, weather, meteorological, or extreme-weather exposure in relation to a dengue outcome. |
| 288 | Travis | 2025 | Topography and environmental deficiencies are associated with chikungunya virus exposure in urban informal settlements in Salvador, Brazil | PLOS NEGLECTED TROPICAL DISEASES | 10.1371/journal.pntd.0013477 | Vector-only study without a dengue-related outcome or context | The study focused on mosquito biology, ecology, abundance, distribution, or control without a substantive dengue-related outcome or analysis. |
| 289 | Kurosu | 2023 | Dengue virus infection induces selective expansion of Vγ4 and Vγ6TCR γδ T cells in the small intestine and a cytokine storm driving vascular leakage in mice | PLOS NEGLECTED TROPICAL DISEASES | 10.1371/journal.pntd.0011743 | Clinical, diagnostic, immunological, pathogenesis, or therapeutic dengue study | The study evaluated dengue clinical features, diagnosis, immune responses, molecular mechanisms, animal models, or treatment without using a climatic or meteorological factor as the exposure. |
| 290 | Groat-Carmona | 2025 | Sex Ratio Distortion of Aedes aegypti (L.) in El Salvador: Biocontrol Implications for Seasonally Dry Urban Neotropical Environments | DIVERSITY-BASEL | 10.3390/d17040257 | Vector-only study without a dengue-related outcome or context | The study focused on mosquito biology, ecology, abundance, distribution, or control without a substantive dengue-related outcome or analysis. |
| 291 | Hu | 2021 | Mosquito Control Based on Pesticides and Endosymbiotic Bacterium Wolbachia | BULLETIN OF MATHEMATICAL BIOLOGY | 10.1007/s11538-021-00881-9 | Vector-control or intervention study without a climate–dengue association | The study evaluated Wolbachia, insecticides, vector control, health education, or mosquito habitats without directly estimating an association between climate/meteorological exposure and a dengue outcome. |
| 292 | Martinez | 2022 | Genomic and Phenotypic Comparisons Reveal Distinct Variants of Wolbachia Strain wAlbB | APPLIED AND ENVIRONMENTAL MICROBIOLOGY | 10.1128/aem.01412-22 | Vector-only study without a dengue-related outcome or context | Dengue was mentioned only to describe the vector importance of the mosquito; the measured outcomes concerned mosquito biology, ecology, or control. |
| 293 | Day | 2016 | Mosquito Oviposition Behavior and Vector Control | INSECTS | 10.3390/insects7040065 | Vector-only study without a dengue-related outcome or context | Dengue was mentioned only to describe the vector importance of the mosquito; the measured outcomes concerned mosquito biology, ecology, or control. |
| 294 | Dayrit | 2018 | Impact of climate change on dermatological conditions related to flooding: update from the International Society of Dermatology Climate Change Committee | INTERNATIONAL JOURNAL OF DERMATOLOGY | 10.1111/ijd.13901 | General climate-health, disaster, or other-disease study without dengue-specific analysis | The study concerned general climate-health, disasters, multiple diseases, or another disease; dengue appeared only as background, in a disease list, or as a secondary context without an identifiable dengue-specific analysis. |
| 295 | Medlock | 2015 | Impacts of the creation, expansion and management of English wetlands on mosquito presence and abundance - developing strategies for future disease mitigation | PARASITES & VECTORS | 10.1186/s13071-015-0751-3 | Vector-only study without a dengue-related outcome or context | Dengue was mentioned only to describe the vector importance of the mosquito; the measured outcomes concerned mosquito biology, ecology, or control. |
| 296 | Malavige | 2022 | Dengue and COVID-19: two sides of the same coin | JOURNAL OF BIOMEDICAL SCIENCE | 10.1186/s12929-022-00833-y | Dengue epidemiology, transmission, or social-factor study without climate-exposure analysis | The study focused on dengue outbreak description, spatial distribution, human mobility, public-health management, or social factors without directly analyzing a climate/meteorological exposure–dengue outcome association. |
| 297 | Martin | 2008 | The impact of climate change on the epidemiology and control of Rift Valley fever | REVUE SCIENTIFIQUE ET TECHNIQUE-OFFICE INTERNATIONAL DES EPIZOOTIES | 10.20506/rst.27.2.1802 | Dengue mentioned only as background or an example | The article discussed climate and health or another disease; dengue appeared only in the background or a disease list and was not analyzed separately. |
| 298 | Bruehl | 2020 | Environmental and socioeconomic effects of mosquito control in Europe using the biocide Bacillus thuringiensis subsp. israelensis (Bti) | SCIENCE OF THE TOTAL ENVIRONMENT | 10.1016/j.scitotenv.2020.137800 | Not dengue-specific | Neither the title nor the abstract treated dengue as a study subject or reported a dengue-related outcome or analysis. |
| 299 | Colozza | 2025 | The impact of climate change on child nutrition in Indonesia: a conceptual framework and scoping review of the available evidence | BMJ PAEDIATRICS OPEN | 10.1136/bmjpo-2024-002980 | Not dengue-specific | Neither the title nor the abstract treated dengue as a study subject or reported a dengue-related outcome or analysis. |
| 300 | Kabir | 2016 | Climate change and health in Bangladesh: a baseline cross-sectional survey | GLOBAL HEALTH ACTION | 10.3402/gha.v9.29609 | Dengue mentioned only as background or an example | Dengue was mentioned only in the background, discussion, or as an example, without an identifiable dengue-specific analysis. |
| 301 | Naik | 2026 | A framework for assessing global health impacts of polar change: An urgent call for interdisciplinary research | AMBIO | 10.1007/s13280-025-02255-0 | Not dengue-specific | Neither the title nor the abstract treated dengue as a study subject or reported a dengue-related outcome or analysis. |
| 302 | AhbiRami | 2020 | School-based health education for dengue control in Kelantan, Malaysia: Impact on knowledge, attitude and practice | PLOS NEGLECTED TROPICAL DISEASES | 10.1371/journal.pntd.0008075 | Vector-control or intervention study without a climate–dengue association | The study evaluated Wolbachia, insecticides, vector control, health education, or mosquito habitats without directly estimating an association between climate/meteorological exposure and a dengue outcome. |
| 303 | Shabbir | 2026 | Animal models illuminate dengue immunopathogenesis to guide vaccine and therapeutic developments | JOURNAL OF VIROLOGICAL METHODS | 10.1016/j.jviromet.2026.115350 | Clinical, diagnostic, immunological, pathogenesis, or therapeutic dengue study | The study evaluated dengue clinical features, diagnosis, immune responses, molecular mechanisms, animal models, or treatment without using a climatic or meteorological factor as the exposure. |
| 304 | Holmes | 2019 | Biological Adaptations Associated with Dehydration in Mosquitoes | INSECTS | 10.3390/insects10110375 | Vector-only study without a dengue-related outcome or context | The study focused on mosquito biology, ecology, abundance, distribution, or control without a substantive dengue-related outcome or analysis. |
| 305 | Mas-Coma | 2025 | Infectious disease risk after the October 2024 flash flood in Valencia, Spain: Disaster evolution, strategic scenario analysis, and extrapolative baseline for a One Health assessment | ONE HEALTH | 10.1016/j.onehlt.2025.101093 | Not dengue-specific | Neither the title nor the abstract treated dengue as a study subject or reported a dengue-related outcome or analysis. |
| 306 | Kim | 2023 | Mitigating the effects of climate change on human health with vaccines and vaccinations | FRONTIERS IN PUBLIC HEALTH | 10.3389/fpubh.2023.1252910 | Dengue mentioned only as background or an example | The article discussed climate and health or another disease; dengue appeared only in the background or a disease list and was not analyzed separately. |
| 307 | Kabir | 2025 | Understanding climate-sensitive diseases in Bangladesh using systematic review and government data repository | PLOS ONE | 10.1371/journal.pone.0313031 | Dengue mentioned only as background or an example | The article discussed climate and health or another disease; dengue appeared only in the background or a disease list and was not analyzed separately. |
| 308 | Harrison | 2009 | RAPID ASSESSMENT OF MOSQUITOES AND ARBOVIRUS ACTIVITY AFTER FLOODS IN SOUTHEASTERN KANSAS, 2007 | JOURNAL OF THE AMERICAN MOSQUITO CONTROL ASSOCIATION | 10.2987/08-5754.1 | Not dengue-specific | Neither the title nor the abstract treated dengue as a study subject or reported a dengue-related outcome or analysis. |
| 309 | Abbasi | 2025 | Climate Change and Vector-Borne Disease Transmission: The Role of Insect Behavioral and Physiological Adaptations | INTEGRATIVE ORGANISMAL BIOLOGY | 10.1093/iob/obaf011 | Vector-only study without a dengue-related outcome or context | The study focused on mosquito biology, ecology, abundance, distribution, or control without a substantive dengue-related outcome or analysis. |
| 310 | Alarcon-Elbal | 2024 | Where does Aedes albopictus (Diptera: Culicidae) really breed in a Mediterranean residential area? Results from a field study in Valencia, Eastern Spain | BULLETIN OF ENTOMOLOGICAL RESEARCH | 10.1017/S0007485324000191 | Vector-only study without a dengue-related outcome or context | Dengue was mentioned only to describe the vector importance of the mosquito; the measured outcomes concerned mosquito biology, ecology, or control. |
| 311 | Hoh | 2014 | A novel rare copy number variant of the ABCF1 gene identified among dengue fever patients from Peninsular Malaysia | GENETICS AND MOLECULAR RESEARCH | 10.4238/2014.February.19.9 | Clinical, diagnostic, immunological, pathogenesis, or therapeutic dengue study | The study evaluated dengue clinical features, diagnosis, immune responses, molecular mechanisms, animal models, or treatment without using a climatic or meteorological factor as the exposure. |
| 312 | Guo | 2019 | The clinical significance of myeloid-derived suppressor cells in dengue fever patients | BMC INFECTIOUS DISEASES | 10.1186/s12879-019-4574-2 | Clinical, diagnostic, immunological, pathogenesis, or therapeutic dengue study | The study evaluated dengue clinical features, diagnosis, immune responses, molecular mechanisms, animal models, or treatment without using a climatic or meteorological factor as the exposure. |
| 313 | Karakullukcu | 2026 | Climate change and water-related diseases: a bibliometric, knowledge map, topic modeling, and content analysis | JOURNAL OF WATER AND HEALTH | 10.2166/wh.2026.252 | General climate-health, disaster, or other-disease study without dengue-specific analysis | The study concerned general climate-health, disasters, multiple diseases, or another disease; dengue appeared only as background, in a disease list, or as a secondary context without an identifiable dengue-specific analysis. |
| 314 | Rajput | 2021 | Jaundice and Thrombocytopenia in an acute abdomen with concurrent Appendicitis and spontaneous Rectal perforation: An unusual presentation of human leptospirosis | TROPICAL DOCTOR | 10.1177/0049475520981298 | General climate-health, disaster, or other-disease study without dengue-specific analysis | The study concerned general climate-health, disasters, multiple diseases, or another disease; dengue appeared only as background, in a disease list, or as a secondary context without an identifiable dengue-specific analysis. |
| 315 | Garcia | 2019 | Matching the genetics of released and local Aedes aegypti populations is critical to assure Wolbachia invasion | PLOS NEGLECTED TROPICAL DISEASES | 10.1371/journal.pntd.0007023 | Vector-only study without a dengue-related outcome or context | Dengue was mentioned only to describe the vector importance of the mosquito; the measured outcomes concerned mosquito biology, ecology, or control. |
| 316 | Saulnier | 2018 | The Effect of Seasonal Floods on Health: Analysis of Six Years of National Health Data and Flood Maps | INTERNATIONAL JOURNAL OF ENVIRONMENTAL RESEARCH AND PUBLIC HEALTH | 10.3390/ijerph15040665 | Not dengue-specific | Neither the title nor the abstract treated dengue as a study subject or reported a dengue-related outcome or analysis. |
| 317 | Deichmeister | 2011 | Abundance of West Nile virus mosquito vectors in relation to climate and landscape variables | JOURNAL OF VECTOR ECOLOGY | 10.1111/j.1948-7134.2011.00143.x | Vector-only study without a dengue-related outcome or context | The study focused on mosquito biology, ecology, abundance, distribution, or control without a substantive dengue-related outcome or analysis. |
| 318 | Amer | 2021 | Effect of Selection for Pyrethroid Resistance on Abiotic Stress Tolerance in Aedes aegypti from Merida, Yucatan, Mexico | INSECTS | 10.3390/insects12020124 | Vector-only study without a dengue-related outcome or context | Dengue was mentioned only to describe the vector importance of the mosquito; the measured outcomes concerned mosquito biology, ecology, or control. |
| 319 | Findlay | 2015 | Cytokines and viral hemorrhagic fever: potential for therapeutic intervention | FUTURE VIROLOGY | 10.2217/FVL.15.5 | Dengue mentioned only as background or an example | Dengue was mentioned only in the background, discussion, or as an example, without an identifiable dengue-specific analysis. |
| 320 | Bin Asad | 2019 | Erupt of malaria, dengue and chikungunya in Pakistan: Recent insights about prevalence, diagnosis and treatment | PAKISTAN JOURNAL OF PHARMACEUTICAL SCIENCES |  | Vector-only study without a dengue-related outcome or context | The measured outcomes concerned vector biology or ecology rather than dengue transmission, incidence, or disease risk. |
| 321 | Semenza | 2021 | Climate change and infectious disease in Europe: Impact, projection and adaptation | LANCET REGIONAL HEALTH-EUROPE | 10.1016/j.lanepe.2021.100230 | Dengue mentioned only as background or an example | Dengue was mentioned only in the background, discussion, or as an example, without an identifiable dengue-specific analysis. |
| 322 | Diem | 2017 | Heat in the southeastern United States: Characteristics, trends, and potential health impact | PLOS ONE | 10.1371/journal.pone.0177937 | Vector-only study without a dengue-related outcome or context | The study focused on mosquito biology, ecology, abundance, distribution, or control without a substantive dengue-related outcome or analysis. |
| 323 | Sehrawat | 2018 | Role of Cytokines as Molecular Marker of Dengue Severity | MEDITERRANEAN JOURNAL OF HEMATOLOGY AND INFECTIOUS DISEASES | 10.4084/MJHID.2018.023 | No relevant climate or meteorological exposure | The study addressed dengue but did not analyze a climatic, weather, meteorological, or extreme-weather exposure in relation to a dengue outcome. |
| 324 | Pradeep | 2021 | Innate Immune Cytokine Profiling and Biomarker Identification for Outcome in Dengue Patients | FRONTIERS IN IMMUNOLOGY | 10.3389/fimmu.2021.677874 | Clinical, diagnostic, immunological, pathogenesis, or therapeutic dengue study | The study evaluated dengue clinical features, diagnosis, immune responses, molecular mechanisms, animal models, or treatment without using a climatic or meteorological factor as the exposure. |
| 325 | Chakraborty | 2025 | Regulation of miRNA in Cytokine Storm (CS) of COVID-19 and Other Viral Infection: An Exhaustive Review | REVIEWS IN MEDICAL VIROLOGY | 10.1002/rmv.70026 | Dengue mentioned only as background or an example | The article discussed climate and health or another disease; dengue appeared only in the background or a disease list and was not analyzed separately. |
| 326 | Watts | 2021 | The rise of West Nile Virus in Southern and Southeastern Europe: A spatial-temporal analysis investigating the combined effects of climate, land use and economic changes | ONE HEALTH | 10.1016/j.onehlt.2021.100315 | Not dengue-specific | Neither the title nor the abstract treated dengue as a study subject or reported a dengue-related outcome or analysis. |
| 327 | Rout | 2011 | HIGH PREVALENCE OF BACTERIAL SPORE-FORMERS ACTIVE AGAINST MOSQUITO LARVAE IN TEMPORARY MONSOON FLOODED SITES IN ORISSA, INDIA | JOURNAL OF THE AMERICAN MOSQUITO CONTROL ASSOCIATION | 10.2987/10-6067.1 | Vector-only study without a dengue-related outcome or context | The study focused on mosquito biology, ecology, abundance, distribution, or control without a substantive dengue-related outcome or analysis. |
| 328 | Guertler | 2025 | Viral sepsis - pathophysiology and disease manifestation | INFECTION | 10.1007/s15010-025-02486-z | No relevant climate or meteorological exposure | The study addressed dengue but did not analyze a climatic, weather, meteorological, or extreme-weather exposure in relation to a dengue outcome. |
| 329 | Vujic | 2010 | SPECIES COMPOSITION AND SEASONAL DYNAMICS OF MOSQUITOES (DIPTERA: CULICIDAE) IN FLOODED AREAS OF VOJVODINA, SERBIA | ARCHIVES OF BIOLOGICAL SCIENCES | 10.2298/ABS1004193V | Vector-only study without a dengue-related outcome or context | The study focused on mosquito biology, ecology, abundance, distribution, or control without a substantive dengue-related outcome or analysis. |
| 330 | Angurana | 2025 | Clinical Profile, Intensive Care Needs, and Short-Term Outcome of Acute Necrotizing Encephalopathy of Childhood: A Retrospective Study From a Tertiary Care Hospital in North India | PEDIATRIC NEUROLOGY | 10.1016/j.pediatrneurol.2025.06.016 | Dengue mentioned only as background or an example | Dengue was mentioned only in the background, discussion, or as an example, without an identifiable dengue-specific analysis. |
| 331 | Bett | 2017 | Effects of flood irrigation on the risk of selected zoonotic pathogens in an arid and semi-arid area in the eastern Kenya | PLOS ONE | 10.1371/journal.pone.0172626 | General climate-health, disaster, or other-disease study without dengue-specific analysis | The study concerned general climate-health, disasters, multiple diseases, or another disease; dengue appeared only as background, in a disease list, or as a secondary context without an identifiable dengue-specific analysis. |
| 332 | Gee | 2022 | EVALUATING THE OCCURRENCE AND RELATIVE ABUNDANCE OF MOSQUITOES IN RAINWATER HARVESTING SYSTEMS | JOURNAL OF THE ASABE | 10.13031/ja.15189 | Vector-only study without a dengue-related outcome or context | The study focused on mosquito biology, ecology, abundance, distribution, or control without a substantive dengue-related outcome or analysis. |
| 333 | Rochlin | 2019 | West Nile Virus Mosquito Vectors in North America | JOURNAL OF MEDICAL ENTOMOLOGY | 10.1093/jme/tjz146 | Vector-only study without a dengue-related outcome or context | The study focused on mosquito biology, ecology, abundance, distribution, or control without a substantive dengue-related outcome or analysis. |
| 334 | Gain | 2015 | An integrated approach of flood risk assessment in the eastern part of Dhaka City | NATURAL HAZARDS | 10.1007/s11069-015-1911-7 | Not dengue-specific | Neither the title nor the abstract treated dengue as a study subject or reported a dengue-related outcome or analysis. |
| 335 | Soti | 2012 | Combining Hydrology and Mosquito Population Models to Identify the Drivers of Rift Valley Fever Emergence in Semi-Arid Regions of West Africa | PLOS NEGLECTED TROPICAL DISEASES | 10.1371/journal.pntd.0001795 | Vector-only study without a dengue-related outcome or context | The study focused on mosquito biology, ecology, abundance, distribution, or control without a substantive dengue-related outcome or analysis. |
| 336 | Masetti | 2026 | Safeguarding future generations: a One Health perspective on children, climate change, and infectious threats | FRONTIERS IN PUBLIC HEALTH | 10.3389/fpubh.2026.1771844 | Not dengue-specific | Neither the title nor the abstract treated dengue as a study subject or reported a dengue-related outcome or analysis. |
| 337 | Rijal | 2020 | Guiding Documents for Disaster Risk Reduction and Management in Health Care System of Nepal | JOURNAL OF NEPAL MEDICAL ASSOCIATION | 10.31729/jnma.5041 | Dengue mentioned only as background or an example | Dengue was mentioned only in the background, discussion, or as an example, without an identifiable dengue-specific analysis. |
| 338 | Jayaramu | 2023 | Leptospirosis modelling using hydrometeorological indices and random forest machine learning | INTERNATIONAL JOURNAL OF BIOMETEOROLOGY | 10.1007/s00484-022-02422-y | Not dengue-specific | Neither the title nor the abstract treated dengue as a study subject or reported a dengue-related outcome or analysis. |
| 339 | Fakhri | 2021 | Modulating Neurological Complications of Emerging Infectious Diseases: Mechanistic Approaches to Candidate Phytochemicals | FRONTIERS IN PHARMACOLOGY | 10.3389/fphar.2021.742146 | Dengue mentioned only as background or an example | The article discussed climate and health or another disease; dengue appeared only in the background or a disease list and was not analyzed separately. |
| 340 | de Vries | 2018 | Leptospirosis among Returned Travelers: A GeoSentinel Site Survey and Multicenter Analysis-1997-2016 | AMERICAN JOURNAL OF TROPICAL MEDICINE AND HYGIENE | 10.4269/ajtmh.18-0020 | Not dengue-specific | Neither the title nor the abstract treated dengue as a study subject or reported a dengue-related outcome or analysis. |
| 341 | Zain | 2024 | Climate change and its impact on infectious diseases in Asia | SINGAPORE MEDICAL JOURNAL | 10.4103/singaporemedj.SMJ-2023-180 | Not dengue-specific | Neither the title nor the abstract treated dengue as a study subject or reported a dengue-related outcome or analysis. |
| 342 | Parker | 2023 | The burden of water insecurity: a review of the challenges to water resource management and connected health risks associated with water stress in small island developing states | JOURNAL OF WATER AND CLIMATE CHANGE | 10.2166/wcc.2023.239 | General climate-health, disaster, or other-disease study without dengue-specific analysis | The study concerned general climate-health, disasters, multiple diseases, or another disease; dengue appeared only as background, in a disease list, or as a secondary context without an identifiable dengue-specific analysis. |
| 343 | Veronesi | 2015 | MACROCYCLOPS ALBIDUS (COPEPODA: CYCLOPIDAE) FOR THE BIOCONTROL OF AEDES ALBOPICTUS AND CULEX PIPIENS IN ITALY | JOURNAL OF THE AMERICAN MOSQUITO CONTROL ASSOCIATION | 10.2987/13-6381.1 | Vector-only study without a dengue-related outcome or context | The study focused on mosquito biology, ecology, abundance, distribution, or control without a substantive dengue-related outcome or analysis. |
| 344 | Medelez | 2026 | Influence of invasive guinea grass on Aedes aegypti behavior in urban semi-arid subtropical environments | BIOLOGICAL INVASIONS | 10.1007/s10530-025-03733-y | Vector-only study without a dengue-related outcome or context | Dengue was mentioned only to describe the vector importance of the mosquito; the measured outcomes concerned mosquito biology, ecology, or control. |
| 345 | Neaterour | 2017 | Case Report: Fatal Leptospira spp./Zika Virus Coinfection-Puerto Rico, 2016 | AMERICAN JOURNAL OF TROPICAL MEDICINE AND HYGIENE | 10.4269/ajtmh.17-0250 | No relevant climate or meteorological exposure | The study addressed dengue but did not analyze a climatic, weather, meteorological, or extreme-weather exposure in relation to a dengue outcome. |
| 346 | Garziera | 2017 | Effect of interruption of over-flooding releases of transgenic mosquitoes over wild population of Aedes aegypti: two case studies in Brazil | ENTOMOLOGIA EXPERIMENTALIS ET APPLICATA | 10.1111/eea.12618 | Vector-only study without a dengue-related outcome or context | The study focused on mosquito biology, ecology, abundance, distribution, or control without a substantive dengue-related outcome or analysis. |
| 347 | Parker | 2019 | DO GUTTER GUARDS AFFECT MOSQUITO PRODUCTION IN ROOF GUTTER HABITATS? | JOURNAL OF THE AMERICAN MOSQUITO CONTROL ASSOCIATION | 10.2987/18-6782.1 | Vector-only study without a dengue-related outcome or context | The study focused on mosquito biology, ecology, abundance, distribution, or control without a substantive dengue-related outcome or analysis. |
| 348 | Ahmed | 2017 | Yellow fever from Angola and Congo: a storm gathers | TROPICAL DOCTOR | 10.1177/0049475517699726 | Dengue mentioned only as background or an example | The article discussed climate and health or another disease; dengue appeared only in the background or a disease list and was not analyzed separately. |
| 349 | Sheffield | 2011 | Global Climate Change and Children's Health: Threats and Strategies for Prevention | ENVIRONMENTAL HEALTH PERSPECTIVES | 10.1289/ehp.1002233 | Dengue mentioned only as background or an example | Dengue was mentioned only in the background, discussion, or as an example, without an identifiable dengue-specific analysis. |
| 350 | Elbers | 2015 | Mosquitoes and Culicoides biting midges: vector range and the influence of climate change | REVUE SCIENTIFIQUE ET TECHNIQUE-OFFICE INTERNATIONAL DES EPIZOOTIES | 10.20506/rst.34.1.2349 | Vector-only study without a dengue-related outcome or context | The study focused on mosquito biology, ecology, abundance, distribution, or control without a substantive dengue-related outcome or analysis. |
| 351 | Crowley | 2025 | Assessing the knowledge, attitudes, and practices (KAP) of dengue in Thailand: a systematic review and meta-analysis | ARCHIVES OF PUBLIC HEALTH | 10.1186/s13690-025-01522-6 | Dengue epidemiology, transmission, or social-factor study without climate-exposure analysis | The study focused on dengue outbreak description, spatial distribution, human mobility, public-health management, or social factors without directly analyzing a climate/meteorological exposure–dengue outcome association. |
| 352 | Rodrigue Simonet | 2020 | Diversity and Abundance of Potential Vectors of Rift Valley Fever Virus in the North Region of Cameroon | INSECTS | 10.3390/insects11110814 | Vector-only study without a dengue-related outcome or context | The study focused on mosquito biology, ecology, abundance, distribution, or control without a substantive dengue-related outcome or analysis. |
| 353 | Hug | 2025 | Avoid the light to avoid the heat? Thermal and light preferences of Aedes aegypti mosquito larvae | ANIMAL BEHAVIOUR | 10.1016/j.anbehav.2024.10.025 | Vector-only study without a dengue-related outcome or context | The study focused on mosquito biology, ecology, abundance, distribution, or control without a substantive dengue-related outcome or analysis. |
| 354 | Biswas | 2020 | Coagulation Disorders in COVID-19: Role of Toll-like Receptors | JOURNAL OF INFLAMMATION RESEARCH | 10.2147/JIR.S271768 | Not dengue-specific | Neither the title nor the abstract treated dengue as a study subject or reported a dengue-related outcome or analysis. |
| 355 | Wang | 2017 | The impact of weather and storm water management ponds on the transmission of West Nile virus | ROYAL SOCIETY OPEN SCIENCE | 10.1098/rsos.170017 | Vector-only study without a dengue-related outcome or context | The study focused on mosquito biology, ecology, abundance, distribution, or control without a substantive dengue-related outcome or analysis. |
| 356 | Park | 2025 | Heat activation desensitizes Aedes aegypti transient receptor potential ankyrin 1 (AaTRPA1) to chemical agonists that repel mosquitoes | PESTICIDE BIOCHEMISTRY AND PHYSIOLOGY | 10.1016/j.pestbp.2025.106326 | Vector-only study without a dengue-related outcome or context | The study focused on mosquito biology, ecology, abundance, distribution, or control without a substantive dengue-related outcome or analysis. |
| 357 | Papadopoulos | 2023 | Beauty and the beast: host microRNA-155 versus SARS-CoV-2 | HUMAN CELL | 10.1007/s13577-023-00867-w | Dengue mentioned only as background or an example | The article discussed climate and health or another disease; dengue appeared only in the background or a disease list and was not analyzed separately. |
| 358 | Monath | 2008 | Treatment of yellow fever | ANTIVIRAL RESEARCH | 10.1016/j.antiviral.2007.10.009 | Dengue mentioned only as background or an example | The article discussed climate and health or another disease; dengue appeared only in the background or a disease list and was not analyzed separately. |
| 359 | See | 2024 | Dengue-Associated Hemophagocytic Lymphohistiocytosis: A Narrative Review of Its Identification and Treatment | PATHOGENS | 10.3390/pathogens13040332 | Clinical, diagnostic, immunological, pathogenesis, or therapeutic dengue study | The study evaluated dengue clinical features, diagnosis, immune responses, molecular mechanisms, animal models, or treatment without using a climatic or meteorological factor as the exposure. |
| 360 | Mpandeli | 2018 | Climate Change Adaptation through the Water-Energy-Food Nexus in Southern Africa | INTERNATIONAL JOURNAL OF ENVIRONMENTAL RESEARCH AND PUBLIC HEALTH | 10.3390/ijerph15102306 | General climate-health, disaster, or other-disease study without dengue-specific analysis | The study concerned general climate-health, disasters, multiple diseases, or another disease; dengue appeared only as background, in a disease list, or as a secondary context without an identifiable dengue-specific analysis. |
| 361 | Hamer | 2011 | EVALUATION OF A NOVEL EMERGENCE TRAP TO STUDY CULEX MOSQUITOES IN URBAN CATCH BASINS | JOURNAL OF THE AMERICAN MOSQUITO CONTROL ASSOCIATION | 10.2987/10-6090.1 | Vector-only study without a dengue-related outcome or context | The study focused on mosquito biology, ecology, abundance, distribution, or control without a substantive dengue-related outcome or analysis. |
| 362 | Viennet | 2025 | Blood under pressure: how climate change threatens blood safety and supply chains | LANCET PLANETARY HEALTH | 10.1016/S2542-5196(25)00051-8 | Not dengue-specific | Neither the title nor the abstract treated dengue as a study subject or reported a dengue-related outcome or analysis. |
| 363 | Srikiatkhachorn | 2017 | Immune-mediated cytokine storm and its role in severe dengue | SEMINARS IN IMMUNOPATHOLOGY | 10.1007/s00281-017-0625-1 | Clinical, diagnostic, immunological, pathogenesis, or therapeutic dengue study | The study evaluated dengue clinical features, diagnosis, immune responses, molecular mechanisms, animal models, or treatment without using a climatic or meteorological factor as the exposure. |
| 364 | Wilke | 2018 | Construction sites in Miami-Dade County, Florida are highly favorable environments for vector mosquitoes | PLOS ONE | 10.1371/journal.pone.0209625 | Vector-only study without a dengue-related outcome or context | The study focused on mosquito biology, ecology, abundance, distribution, or control without a substantive dengue-related outcome or analysis. |
| 365 | Ricardo | 2018 | Knowledge, attitudes and practices (KAP) regarding leptospirosis among residents of riverside settlements of Santa Fe, Argentina | PLOS NEGLECTED TROPICAL DISEASES | 10.1371/journal.pntd.0006470 | Not dengue-specific | Neither the title nor the abstract treated dengue as a study subject or reported a dengue-related outcome or analysis. |
| 366 | Moise | 2024 | Effects of Hurricane Irma on mosquito abundance and species composition in a metropolitan Gulf coastal city, 2016-2018 | SCIENTIFIC REPORTS | 10.1038/s41598-024-72734-z | Vector-only study without a dengue-related outcome or context | The study focused on mosquito biology, ecology, abundance, distribution, or control without a substantive dengue-related outcome or analysis. |
| 367 | Tang | 2019 | Estimating human health damage factors related to CO2 emissions by considering updated climate-related relative risks | INTERNATIONAL JOURNAL OF LIFE CYCLE ASSESSMENT | 10.1007/s11367-018-1561-6 | Dengue mentioned only as background or an example | Dengue was mentioned only in the background, discussion, or as an example, without an identifiable dengue-specific analysis. |
| 368 | Cabanova | 2018 | Urbanization impact on mosquito community and the transmission potential of filarial infection in central Europe | PARASITES & VECTORS | 10.1186/s13071-018-2845-1 | Vector-only study without a dengue-related outcome or context | The study focused on mosquito biology, ecology, abundance, distribution, or control without a substantive dengue-related outcome or analysis. |
| 369 | Iyer | 2022 | Pathophysiologic and prognostic role of proinflammatory and regulatory cytokines as a proinflammatory and regulatory cytokine in dengue fever* | INDIAN JOURNAL OF MEDICAL MICROBIOLOGY | 10.1016/j.ijmmb.2022.01.010 | Clinical, diagnostic, immunological, pathogenesis, or therapeutic dengue study | The study evaluated dengue clinical features, diagnosis, immune responses, molecular mechanisms, animal models, or treatment without using a climatic or meteorological factor as the exposure. |
| 370 | Diouf | 2020 | Zika virus in southeastern Senegal: survival of the vectors and the virus during the dry season | BMC INFECTIOUS DISEASES | 10.1186/s12879-020-05093-5 | Vector-only study without a dengue-related outcome or context | The study focused on mosquito biology, ecology, abundance, distribution, or control without a substantive dengue-related outcome or analysis. |
| 371 | Nisar | 2024 | Mortality Associated with Priority Diseases in Flood-Affected Areas Using District Health Information System (DHIS2) During September-December 2022: Pakistan Experience | DISASTER MEDICINE AND PUBLIC HEALTH PREPAREDNESS | 10.1017/dmp.2024.77 | Dengue mentioned only as background or an example | Dengue was mentioned only in the background, discussion, or as an example, without an identifiable dengue-specific analysis. |
| 372 | Di Pol | 2022 | Modelling the temperature suitability for the risk of West Nile Virus establishment in European Culex pipiens populations | TRANSBOUNDARY AND EMERGING DISEASES | 10.1111/tbed.14513 | Vector-only study without a dengue-related outcome or context | The study focused on mosquito biology, ecology, abundance, distribution, or control without a substantive dengue-related outcome or analysis. |
| 373 | Accoti | 2025 | Dehydration-induced Ae-Aper50 regulates midgut infection in Aedes aegypti mosquitoes | MBIO | 10.1128/mbio.01207-24 | Vector-only study without a dengue-related outcome or context | The study focused on mosquito biology, ecology, abundance, distribution, or control without a substantive dengue-related outcome or analysis. |
| 374 | Johnson | 2012 | The roles of mosquito and bird communities on the prevalence of West Nile virus in urban wetland and residential habitats | URBAN ECOSYSTEMS | 10.1007/s11252-012-0248-1 | Vector-only study without a dengue-related outcome or context | The study focused on mosquito biology, ecology, abundance, distribution, or control without a substantive dengue-related outcome or analysis. |
| 375 | Sun | 2012 | Host Cytokine Storm Is Associated With Disease Severity of Severe Fever With Thrombocytopenia Syndrome | JOURNAL OF INFECTIOUS DISEASES | 10.1093/infdis/jis452 | Not dengue-specific | Neither the title nor the abstract treated dengue as a study subject or reported a dengue-related outcome or analysis. |
| 376 | Prapty | 2022 | Coinfection of COVID-19 and Dengue: A Case Report | FRONTIERS IN MEDICINE | 10.3389/fmed.2022.872627 | Clinical, diagnostic, immunological, pathogenesis, or therapeutic dengue study | The study evaluated dengue clinical features, diagnosis, immune responses, molecular mechanisms, animal models, or treatment without using a climatic or meteorological factor as the exposure. |
| 377 | Dechet | 2012 | Leptospirosis Outbreak following Severe Flooding: A Rapid Assessment and Mass Prophylaxis Campaign; Guyana, January-February 2005 | PLOS ONE | 10.1371/journal.pone.0039672 | Not dengue-specific | Neither the title nor the abstract treated dengue as a study subject or reported a dengue-related outcome or analysis. |
| 378 | Cloud | 2025 | Evaluating vector mosquito occurrence in residential rain barrels in central Illinois | JOURNAL OF MEDICAL ENTOMOLOGY | 10.1093/jme/tjaf019 | Vector-only study without a dengue-related outcome or context | The study focused on mosquito biology, ecology, abundance, distribution, or control without a substantive dengue-related outcome or analysis. |
| 379 | Alto | 2014 | Reproductive Biology and Susceptibility of Florida Culex coronator to Infection with West Nile Virus | VECTOR-BORNE AND ZOONOTIC DISEASES | 10.1089/vbz.2013.1501 | Vector-only study without a dengue-related outcome or context | The study focused on mosquito biology, ecology, abundance, distribution, or control without a substantive dengue-related outcome or analysis. |
| 380 | Sahimin | 2024 | Seroprevalence of dengue and chikungunya viruses among urban refugees in Klang Valley, Malaysia | TRANSACTIONS OF THE ROYAL SOCIETY OF TROPICAL MEDICINE AND HYGIENE | 10.1093/trstmh/trad097 | Vector-only study without a dengue-related outcome or context | The measured outcomes concerned vector biology or ecology rather than dengue transmission, incidence, or disease risk. |
| 381 | Dhara | 2013 | Climate change & infectious diseases in India: Implications for health care providers | INDIAN JOURNAL OF MEDICAL RESEARCH |  | Dengue mentioned only as background or an example | The article discussed climate and health or another disease; dengue appeared only in the background or a disease list and was not analyzed separately. |
| 382 | Prummongkol | 2019 | EFFICACY OF LIQUID BACTERIAL FORMULATION OF BACILLUS THURINGIENSIS SUBSP. ISRAELENSIS AND BACILLUS SPHAERICUS 1593 MIXTURE IN CONTROLLING MOSQUITO LARVAE IN BANGKOK | SOUTHEAST ASIAN JOURNAL OF TROPICAL MEDICINE AND PUBLIC HEALTH |  | Vector-only study without a dengue-related outcome or context | The study focused on mosquito biology, ecology, abundance, distribution, or control without a substantive dengue-related outcome or analysis. |
| 383 | Rodriguez-Valero | 2018 | Leptospirosis in Spanish travelers returning from Chiang Mai: A case series | TRAVEL MEDICINE AND INFECTIOUS DISEASE | 10.1016/j.tmaid.2018.02.013 | Dengue mentioned only as background or an example | The article discussed climate and health or another disease; dengue appeared only in the background or a disease list and was not analyzed separately. |
| 384 | Wichgers Schreur | 2021 | Reproducing the Rift Valley fever virus mosquito-lamb-mosquito transmission cycle | SCIENTIFIC REPORTS | 10.1038/s41598-020-79267-1 | Vector-only study without a dengue-related outcome or context | The study focused on mosquito biology, ecology, abundance, distribution, or control without a substantive dengue-related outcome or analysis. |
| 385 | My | 2010 | Comprehensive analyses and characterization of haemophagocytic lymphohistiocytosis in Vietnamese children | BRITISH JOURNAL OF HAEMATOLOGY | 10.1111/j.1365-2141.2009.07957.x | No relevant climate or meteorological exposure | The study addressed dengue but did not analyze a climatic, weather, meteorological, or extreme-weather exposure in relation to a dengue outcome. |
| 386 | Unlu | 2021 | Heterodissemination: precision insecticide delivery to mosquito larval habitats by cohabiting vertebrates | SCIENTIFIC REPORTS | 10.1038/s41598-021-93492-2 | Vector-only study without a dengue-related outcome or context | The study focused on mosquito biology, ecology, abundance, distribution, or control without a substantive dengue-related outcome or analysis. |
| 387 | Rey | 2012 | North American Wetlands and Mosquito Control | INTERNATIONAL JOURNAL OF ENVIRONMENTAL RESEARCH AND PUBLIC HEALTH | 10.3390/ijerph9124537 | Vector-only study without a dengue-related outcome or context | The study focused on mosquito biology, ecology, abundance, distribution, or control without a substantive dengue-related outcome or analysis. |
| 388 | Tan | 2010 | A Non Mouse-Adapted Dengue Virus Strain as a New Model of Severe Dengue Infection in AG129 Mice | PLOS NEGLECTED TROPICAL DISEASES | 10.1371/journal.pntd.0000672 | Clinical, diagnostic, immunological, pathogenesis, or therapeutic dengue study | The study evaluated dengue clinical features, diagnosis, immune responses, molecular mechanisms, animal models, or treatment without using a climatic or meteorological factor as the exposure. |
| 389 | Cavalerie | 2015 | A Stochastic Model to Study Rift Valley Fever Persistence with Different Seasonal Patterns of Vector Abundance: New Insights on the Endemicity in the Tropical Island of Mayotte | PLOS ONE | 10.1371/journal.pone.0130838 | Vector-only study without a dengue-related outcome or context | The study focused on mosquito biology, ecology, abundance, distribution, or control without a substantive dengue-related outcome or analysis. |
| 390 | Maucourant | 2019 | Zika virus in the eye of the cytokine storm | EUROPEAN CYTOKINE NETWORK | 10.1684/ecn.2019.0433 | Vector-only study without a dengue-related outcome or context | The study focused on mosquito biology, ecology, abundance, distribution, or control without a substantive dengue-related outcome or analysis. |
| 391 | Kusirisin | 2023 | Acute kidney injury in the tropics | NEPHROLOGY | 10.1111/nep.14118 | Dengue mentioned only as background or an example | Dengue was mentioned only in the background, discussion, or as an example, without an identifiable dengue-specific analysis. |
| 392 | He | 2024 | Investigating the Nexus of NLRP3 Inflammasomes and COVID-19 Pathogenesis: Unraveling Molecular Triggers and Therapeutic Strategies | VIRUSES-BASEL | 10.3390/v16020213 | Not dengue-specific | Neither the title nor the abstract treated dengue as a study subject or reported a dengue-related outcome or analysis. |
| 393 | Moreira | 2024 | Impact of urban disturbance on soil insect communities in a Brazilian Atlantic Forest biological station | URBAN ECOSYSTEMS | 10.1007/s11252-024-01613-2 | Not dengue-specific | Neither the title nor the abstract treated dengue as a study subject or reported a dengue-related outcome or analysis. |
| 394 | Caillouet | 2013 | Nesting Bird “Host Funnel” Increases Mosquito-Bird Contact Rate | JOURNAL OF MEDICAL ENTOMOLOGY | 10.1603/ME12183 | Vector-only study without a dengue-related outcome or context | The study focused on mosquito biology, ecology, abundance, distribution, or control without a substantive dengue-related outcome or analysis. |
| 395 | Rosser | 2022 | Reemergence of yellow fever virus in southeastern Brazil, 2017-2018: What sparked the spread? | PLOS NEGLECTED TROPICAL DISEASES | 10.1371/journal.pntd.0010133 | Not dengue-specific | Neither the title nor the abstract treated dengue as a study subject or reported a dengue-related outcome or analysis. |
| 396 | Wooldridge | 2020 | Pediatric Critical Care and the Climate Emergency: Our Responsibilities and a Call for Change | FRONTIERS IN PEDIATRICS | 10.3389/fped.2020.00472 | Dengue mentioned only as background or an example | Dengue was mentioned only in the background, discussion, or as an example, without an identifiable dengue-specific analysis. |
| 397 | Calzolari | 2012 | Mosquito, Bird and Human Surveillance of West Nile and Usutu Viruses in Emilia-Romagna Region (Italy) in 2010 | PLOS ONE | 10.1371/journal.pone.0038058 | Vector-only study without a dengue-related outcome or context | The study focused on mosquito biology, ecology, abundance, distribution, or control without a substantive dengue-related outcome or analysis. |
| 398 | Russell | 2022 | Both consumptive and non-consumptive effects of predators impact mosquito populations and have implications for disease transmission | ELIFE | 10.7554/eLife.71503; 10.7554/eLife.71503.sa0; 10.7554/eLife.71503.sa1; 10.7554/eLife.71503.sa2 | Vector-only study without a dengue-related outcome or context | The study focused on mosquito biology, ecology, abundance, distribution, or control without a substantive dengue-related outcome or analysis. |
| 399 | Shammi | 2020 | COVID-19 pandemic, socioeconomic crisis and human stress in resource-limited settings: A case from Bangladesh | HELIYON | 10.1016/j.heliyon.2020.e04063 | Dengue mentioned only as background or an example | The article discussed climate and health or another disease; dengue appeared only in the background or a disease list and was not analyzed separately. |
| 400 | Bello | 2023 | Comprehensive risk management of hydrometeorological disaster: A participatory approach in the metropolitan area of Puerto Vallarta, Mexico | INTERNATIONAL JOURNAL OF DISASTER RISK REDUCTION | 10.1016/j.ijdrr.2023.103578 | Dengue mentioned only as background or an example | Dengue was mentioned only in the background, discussion, or as an example, without an identifiable dengue-specific analysis. |
| 401 | Dionne | 2024 | Climate Change and Meningoencephalitis in the Americas: A Brewing Storm | CURRENT INFECTIOUS DISEASE REPORTS | 10.1007/s11908-024-00843-x | Not dengue-specific | Neither the title nor the abstract treated dengue as a study subject or reported a dengue-related outcome or analysis. |
| 402 | Ebi | 2018 | Health risks of warming of 1.5°C, 2°C, and higher, above pre-industrial temperatures | ENVIRONMENTAL RESEARCH LETTERS | 10.1088/1748-9326/aac4bd | Not dengue-specific | Neither the title nor the abstract treated dengue as a study subject or reported a dengue-related outcome or analysis. |
| 403 | Bergman | 2021 | Wolbachia prevalence in the vector species Culex pipiens and Culex torrentium in a Sindbis virus-endemic region of Sweden | PARASITES & VECTORS | 10.1186/s13071-021-04937-6 | Not dengue-specific | Neither the title nor the abstract treated dengue as a study subject or reported a dengue-related outcome or analysis. |
| 404 | de Castro | 2025 | Carry-over effects in Culex species along a land use gradient with differences in microclimatic conditions | PARASITES & VECTORS | 10.1186/s13071-025-06903-y | Vector-only study without a dengue-related outcome or context | The study focused on mosquito biology, ecology, abundance, distribution, or control without a substantive dengue-related outcome or analysis. |
| 405 | Su | 2022 | EVALUATION ON THE ACTIVITY AND EFFICACY OF OMNIPRENE™ WSP AND XWSP AGAINST THE SOUTHERN HOUSE MOSQUITO CULEX QUINQUEFASCIATUS IN SIMULATED CATCH BASINS | JOURNAL OF THE AMERICAN MOSQUITO CONTROL ASSOCIATION |  | Vector-only study without a dengue-related outcome or context | The study focused on mosquito biology, ecology, abundance, distribution, or control without a substantive dengue-related outcome or analysis. |
| 406 | Rella | 2014 | Development of imaging techniques to study the pathogenesis of biosafety level 2/3 infectious agents | PATHOGENS AND DISEASE | 10.1111/2049-632X.12199 | Dengue mentioned only as background or an example | Dengue was mentioned only in the background, discussion, or as an example, without an identifiable dengue-specific analysis. |
| 407 | Jackson | 2009 | Culex Mosquitoes, West Nile Virus, and the Application of Innovative Management in the Design and Management of Stormwater Retention Ponds in Canada | WATER QUALITY RESEARCH JOURNAL OF CANADA | 10.2166/wqrj.2009.011 | Vector-only study without a dengue-related outcome or context | The study focused on mosquito biology, ecology, abundance, distribution, or control without a substantive dengue-related outcome or analysis. |
| 408 | Johnson | 2014 | The effects of forced-egg retention on the blood-feeding behavior and reproductive potential of Culex pipiens (Diptera: Culicidae) | JOURNAL OF INSECT PHYSIOLOGY | 10.1016/j.jinsphys.2014.05.014 | Vector-only study without a dengue-related outcome or context | The study focused on mosquito biology, ecology, abundance, distribution, or control without a substantive dengue-related outcome or analysis. |
| 409 | Suliman | 2025 | Cappable-Seq reveals the transcriptional landscape of stress responses in the bacterial endosymbiont Wolbachia | MICROBIAL GENOMICS | 10.1099/mgen.0.001542 | Vector-only study without a dengue-related outcome or context | The study focused on mosquito biology, ecology, abundance, distribution, or control without a substantive dengue-related outcome or analysis. |
| 410 | Sasmita | 2019 | Effects of larval diets and temperature regimes on life history traits, energy reserves and temperature tolerance of male Aedes aegypti (Diptera: Culicidae): optimizing rearing techniques for the sterile insect programmes | PARASITES & VECTORS | 10.1186/s13071-019-3830-z | Vector-only study without a dengue-related outcome or context | The study focused on mosquito biology, ecology, abundance, distribution, or control without a substantive dengue-related outcome or analysis. |
| 411 | Ghasemian | 2019 | Eucalyptus camaldulensis properties for use in the eradication of infections | COMPARATIVE IMMUNOLOGY MICROBIOLOGY AND INFECTIOUS DISEASES | 10.1016/j.cimid.2019.04.007 | Not dengue-specific | Neither the title nor the abstract treated dengue as a study subject or reported a dengue-related outcome or analysis. |
| 412 | Ben-Shachar | 2015 | Minimal within-host dengue models highlight the specific roles of the immune response in primary and secondary dengue infections | JOURNAL OF THE ROYAL SOCIETY INTERFACE | 10.1098/rsif.2014.0886 | Clinical, diagnostic, immunological, pathogenesis, or therapeutic dengue study | The study evaluated dengue clinical features, diagnosis, immune responses, molecular mechanisms, animal models, or treatment without using a climatic or meteorological factor as the exposure. |
| 413 | Alsaikhan | 2026 | Phytochemical-Based Immunomodulation: A Promising Therapeutic Approach for Viral Infections | PHYTOTHERAPY RESEARCH | 10.1002/ptr.70143 | Dengue mentioned only as background or an example | Dengue was mentioned only in the background, discussion, or as an example, without an identifiable dengue-specific analysis. |
| 414 | Ferreira | 2025 | Mosquito biodiversity patterns in a region of intense anthropogenic pressure in the Upper Madeira River, Western Amazonia | COMMUNITY ECOLOGY | 10.1007/s42974-025-00266-1 | Vector-only study without a dengue-related outcome or context | The study focused on mosquito biology, ecology, abundance, distribution, or control without a substantive dengue-related outcome or analysis. |
| 415 | Agampodi | 2016 | Redefining Gold Standard Testing for Diagnosing Leptospirosis: Further Evidence from a Well-Characterized, Flood-Related Outbreak in Sri Lanka | AMERICAN JOURNAL OF TROPICAL MEDICINE AND HYGIENE | 10.4269/ajtmh.16-0033 | Not dengue-specific | Neither the title nor the abstract treated dengue as a study subject or reported a dengue-related outcome or analysis. |
| 416 | Nobre Lamarao | 2023 | Pentaclethra macroloba: A Review of the Biological, Pharmacological, Phytochemical, Cosmetic, Nutritional and Biofuel Potential of this Amazonian Plant | PLANTS-BASEL | 10.3390/plants12061330 | Vector-only study without a dengue-related outcome or context | The study focused on mosquito biology, ecology, abundance, distribution, or control without a substantive dengue-related outcome or analysis. |
| 417 | Baak-Baak | 2014 | Urban Mosquito Fauna in Merida City, Mexico: Immatures Collected from Containers and Storm-water Drains/Catch Basins | SOUTHWESTERN ENTOMOLOGIST | 10.3958/059.039.0207 | Not dengue-specific | Neither the title nor the abstract treated dengue as a study subject or reported a dengue-related outcome or analysis. |
| 418 | Hong | 2025 | The relationship between ferroptosis and respiratory infectious diseases: a novel landscape for therapeutic approach | FRONTIERS IN IMMUNOLOGY | 10.3389/fimmu.2025.1550968 | Dengue mentioned only as background or an example | The article discussed climate and health or another disease; dengue appeared only in the background or a disease list and was not analyzed separately. |
| 419 | Alomari | 2024 | Assessing the antiviral potential of melatonin: A comprehensive systematic review | REVIEWS IN MEDICAL VIROLOGY | 10.1002/rmv.2499 | Dengue mentioned only as background or an example | Dengue was mentioned only in the background, discussion, or as an example, without an identifiable dengue-specific analysis. |
| 420 | Benskin | 2020 | A Basic Review of the Preliminary Evidence That COVID-19 Risk and Severity Is Increased in Vitamin D Deficiency | FRONTIERS IN PUBLIC HEALTH | 10.3389/fpubh.2020.00513 | General climate-health, disaster, or other-disease study without dengue-specific analysis | The study concerned general climate-health, disasters, multiple diseases, or another disease; dengue appeared only as background, in a disease list, or as a secondary context without an identifiable dengue-specific analysis. |
| 421 | Feng | 2019 | Effect of imidacloprid exposure on life history traits in the agricultural generalist predator Paederus beetle: Lack of fitness cost but strong hormetic effect and skewed sex ratio | ECOTOXICOLOGY AND ENVIRONMENTAL SAFETY | 10.1016/j.ecoenv.2019.03.003 | Not dengue-specific | Neither the title nor the abstract treated dengue as a study subject or reported a dengue-related outcome or analysis. |
| 422 | Harbison | 2009 | Evaluation of factors for rapid development of Culex quinquefasciatus in belowground stormwater treatment devices | JOURNAL OF VECTOR ECOLOGY |  | Vector-only study without a dengue-related outcome or context | The study focused on mosquito biology, ecology, abundance, distribution, or control without a substantive dengue-related outcome or analysis. |
| 423 | Buckner | 2011 | Weather and landscape factors associated with adult mosquito abundance in southwestern Georgia, USA | JOURNAL OF VECTOR ECOLOGY | 10.1111/j.1948-7134.2011.00167.x | Vector-only study without a dengue-related outcome or context | The study focused on mosquito biology, ecology, abundance, distribution, or control without a substantive dengue-related outcome or analysis. |
| 424 | Yang | 2008 | ENDOGENOUS AND EXOGENOUS FACTORS CONTROLLING TEMPORAL ABUNDANCE PATTERNS OF TROPICAL MOSQUITOES | ECOLOGICAL APPLICATIONS | 10.1890/07-1209.1 | Vector-only study without a dengue-related outcome or context | The study focused on mosquito biology, ecology, abundance, distribution, or control without a substantive dengue-related outcome or analysis. |
| 425 | Sy | 2019 | Effects of insecticide resistance on the reproductive potential of two sub-strains of the malaria vector Anopheles coluzzii | JOURNAL OF VECTOR BORNE DISEASES | 10.4103/0972-9062.289401 | Vector-only study without a dengue-related outcome or context | The study focused on mosquito biology, ecology, abundance, distribution, or control without a substantive dengue-related outcome or analysis. |
| 426 | Chaturvedi | 2021 | T-cell activation profiles distinguish hemophagocytic lymphohistiocytosis and early sepsis | BLOOD | 10.1182/blood.2020009499 | Not dengue-specific | Neither the title nor the abstract treated dengue as a study subject or reported a dengue-related outcome or analysis. |
| 427 | Yuan | 2021 | Quantification of Cytokine Storms During Virus Infections | FRONTIERS IN IMMUNOLOGY | 10.3389/fimmu.2021.659419 | Dengue mentioned only as background or an example | Dengue was mentioned only in the background, discussion, or as an example, without an identifiable dengue-specific analysis. |
| 428 | Zhao | 2025 | Microbiota Composition Associates With Mosquito Productivity Outcomes in Belowground Larval Habitats | MOLECULAR ECOLOGY | 10.1111/mec.17614 | Vector-only study without a dengue-related outcome or context | The study focused on mosquito biology, ecology, abundance, distribution, or control without a substantive dengue-related outcome or analysis. |
| 429 | Wai | 2012 | Community-centred eco-bio-social approach to control dengue vectors: an intervention study from Myanmar | PATHOGENS AND GLOBAL HEALTH | 10.1179/2047773212Y.0000000057 | Vector-control or intervention study without a climate–dengue association | The study evaluated Wolbachia, insecticides, vector control, health education, or mosquito habitats without directly estimating an association between climate/meteorological exposure and a dengue outcome. |
| 430 | Pires | 2010 | Mosquito fauna inhabiting water bodies in the urban environment of Cordoba city, Argentina, following a St. Louis encephalitis outbreak | JOURNAL OF VECTOR ECOLOGY | 10.1111/j.1948-7134.2010.00099.x | Vector-only study without a dengue-related outcome or context | The study focused on mosquito biology, ecology, abundance, distribution, or control without a substantive dengue-related outcome or analysis. |
| 431 | Duchet | 2020 | Effect of desiccation on mosquito oviposition site selection in Mediterranean temporary habitats | ECOLOGICAL ENTOMOLOGY | 10.1111/een.12821 | Vector-only study without a dengue-related outcome or context | The study focused on mosquito biology, ecology, abundance, distribution, or control without a substantive dengue-related outcome or analysis. |
| 432 | Thompson | 2025 | Mosquitoes then and now: culicine mosquito research in and around Ndumo Game Reserve, KwaZulu-Natal | AFRICAN JOURNAL OF WILDLIFE RESEARCH | 10.3957/056.055.0093 | Vector-only study without a dengue-related outcome or context | The study focused on mosquito biology, ecology, abundance, distribution, or control without a substantive dengue-related outcome or analysis. |
| 433 | Schierer | 2025 | An ecological and social approach to the distribution of vector and nuisance mosquito species across residential land use types | JOURNAL OF MEDICAL ENTOMOLOGY | 10.1093/jme/tjaf056 | Vector-only study without a dengue-related outcome or context | The study focused on mosquito biology, ecology, abundance, distribution, or control without a substantive dengue-related outcome or analysis. |
| 434 | Satyam | 2021 | miRNAs in SARS-CoV 2: A Spoke in the Wheel of Pathogenesis | CURRENT PHARMACEUTICAL DESIGN | 10.2174/1381612826999201001200529 | Dengue mentioned only as background or an example | The article discussed climate and health or another disease; dengue appeared only in the background or a disease list and was not analyzed separately. |
| 435 | Scheuch | 2018 | Detection of Usutu, Sindbis, and Batai Viruses in Mosquitoes (Diptera: Culicidae) Collected in Germany, 2011-2016 | VIRUSES-BASEL | 10.3390/v10070389 | Not dengue-specific | Neither the title nor the abstract treated dengue as a study subject or reported a dengue-related outcome or analysis. |
| 436 | Su | 2024 | Breeding sites, migration paths and phylogenetic relationships of mosquitoes in seven cities in northern and southern China | TROPICAL BIOMEDICINE | 10.47665/tb.41.4.008 | Vector-only study without a dengue-related outcome or context | The study focused on mosquito biology, ecology, abundance, distribution, or control without a substantive dengue-related outcome or analysis. |
| 437 | Dhakad | 2019 | Biological, nutritional, and therapeutic significance of Moringa oleifera Lam | PHYTOTHERAPY RESEARCH | 10.1002/ptr.6475 | Not dengue-specific | Neither the title nor the abstract treated dengue as a study subject or reported a dengue-related outcome or analysis. |
| 438 | Aboagye-Antwi | 2010 | Effects of larval growth condition and water availability on desiccation resistance and its physiological basis in adult Anopheles gambiae sensu stricto | MALARIA JOURNAL | 10.1186/1475-2875-9-225 | Vector-only study without a dengue-related outcome or context | The study focused on mosquito biology, ecology, abundance, distribution, or control without a substantive dengue-related outcome or analysis. |
| 439 | Popko | 2018 | Evaluation of Two Entomopathogenic Fungi for Control of Culex quinquefasciatus (Diptera: Culicidae) in Underground Storm Drains in the Coachella Valley, California, United States | JOURNAL OF MEDICAL ENTOMOLOGY | 10.1093/jme/tjx233 | Vector-only study without a dengue-related outcome or context | The study focused on mosquito biology, ecology, abundance, distribution, or control without a substantive dengue-related outcome or analysis. |
| 440 | Papadopoulos | 2021 | Age and genotype dependent erythropoietin protection in COVID-19 | WORLD JOURNAL OF STEM CELLS | 10.4252/wjsc.v13.i10.1513 | General climate-health, disaster, or other-disease study without dengue-specific analysis | The study concerned general climate-health, disasters, multiple diseases, or another disease; dengue appeared only as background, in a disease list, or as a secondary context without an identifiable dengue-specific analysis. |
| 441 | Al Kadi | 2025 | Cytokine storm and vascular leakage in severe dengue: insights from single-cell RNA profiling | LIFE SCIENCE ALLIANCE | 10.26508/lsa.202403008 | Clinical, diagnostic, immunological, pathogenesis, or therapeutic dengue study | The study evaluated dengue clinical features, diagnosis, immune responses, molecular mechanisms, animal models, or treatment without using a climatic or meteorological factor as the exposure. |
| 442 | Wong | 2017 | Current and future developments in the treatment of virus-induced hypercytokinemia | FUTURE MEDICINAL CHEMISTRY | 10.4155/fmc-2016-0181 | Dengue mentioned only as background or an example | Dengue was mentioned only in the background, discussion, or as an example, without an identifiable dengue-specific analysis. |
| 443 | Meireles | 2025 | Do the microbiota of larval breeding site and the blood meal influence the composition and diversity of bacterial communities in the midgut of Mansonia humeralis (Diptera: Culicidae) from the western Amazon? | BRAZILIAN JOURNAL OF MICROBIOLOGY | 10.1007/s42770-025-01623-y | Vector-only study without a dengue-related outcome or context | The study focused on mosquito biology, ecology, abundance, distribution, or control without a substantive dengue-related outcome or analysis. |
| 444 | Ferguson | 2019 | Demographic risk factors for adverse birth outcomes in Puerto Rico in the PROTECT cohort | PLOS ONE | 10.1371/journal.pone.0217770 | Dengue mentioned only as background or an example | Dengue was mentioned only in the background, discussion, or as an example, without an identifiable dengue-specific analysis. |
| 445 | Tallman | 2024 | Waterscapes meet socio-ecological models: A relational framework to examine water insecurity and human health and well-being | PEOPLE AND NATURE | 10.1002/pan3.10635 | Dengue mentioned only as background or an example | Dengue was mentioned only in the background, discussion, or as an example, without an identifiable dengue-specific analysis. |
| 446 | Do Nascimento | 2025 | Culicidae Fauna (Diptera: Culicomorpha) of the Municipality of Mazagão, Amapá, in the Brazilian Amazon | INSECTS | 10.3390/insects16101036 | Vector-only study without a dengue-related outcome or context | Dengue was mentioned only to describe the vector importance of the mosquito; the measured outcomes concerned mosquito biology, ecology, or control. |
| 447 | Barik | 2026 | Mapping the Insecticide Resistance Landscape of Malaria Vectors in Odisha, India (1993-2024): A Scoping Review | JOURNAL OF TROPICAL MEDICINE | 10.1155/jotm/8879127 | Dengue mentioned only as background or an example | The article discussed climate and health or another disease; dengue appeared only in the background or a disease list and was not analyzed separately. |
| 448 | Menezes | 2021 | Natural biflavonoids as potential therapeutic agents against microbial diseases | SCIENCE OF THE TOTAL ENVIRONMENT | 10.1016/j.scitotenv.2021.145168 | Dengue mentioned only as background or an example | Dengue was mentioned only in the background, discussion, or as an example, without an identifiable dengue-specific analysis. |
| 449 | Edwards | 2021 | Patterns of serum immune biomarkers during elephant endotheliotropic herpesvirus viremia in Asian and African elephants | PLOS ONE | 10.1371/journal.pone.0252175 | Not dengue-specific | Neither the title nor the abstract treated dengue as a study subject or reported a dengue-related outcome or analysis. |
| 450 | Marietta | 2026 | Impact of Viral Infections on the Hemostatic System | SEMINARS IN THROMBOSIS AND HEMOSTASIS | 10.1055/a-2601-9302 | Not dengue-specific | Neither the title nor the abstract treated dengue as a study subject or reported a dengue-related outcome or analysis. |
| 451 | Caballero-Garcia | 2021 | The Immunomodulatory Function of Vitamin D, with Particular Reference to SARS-CoV-2 | MEDICINA-LITHUANIA | 10.3390/medicina57121321 | Dengue mentioned only as background or an example | The article discussed climate and health or another disease; dengue appeared only in the background or a disease list and was not analyzed separately. |
| 452 | Kounis | 2021 | COVID-19 Disease, Women's Predominant Non-Heparin Vaccine-Induced Thrombotic Thrombocytopenia and Kounis Syndrome: A Passepartout Cytokine Storm Interplay | BIOMEDICINES | 10.3390/biomedicines9080959 | Dengue mentioned only as background or an example | The article discussed climate and health or another disease; dengue appeared only in the background or a disease list and was not analyzed separately. |
| 453 | Zolfaghari | 2022 | SARS-CoV-2 vaccines: A double-edged sword throughout rapid evolution of COVID-19 | CELL BIOLOGY INTERNATIONAL | 10.1002/cbin.11903 | Not dengue-specific | Neither the title nor the abstract treated dengue as a study subject or reported a dengue-related outcome or analysis. |
| 454 | Vyas | 2021 | Vitamin D in Prevention and Treatment of COVID-19: Current Perspective and Future Prospects | JOURNAL OF THE AMERICAN COLLEGE OF NUTRITION | 10.1080/07315724.2020.1806758 | Dengue mentioned only as background or an example | The article discussed climate and health or another disease; dengue appeared only in the background or a disease list and was not analyzed separately. |
| 455 | Faull | 2016 | Desiccation survival time for eggs of a widespread and invasive Australian mosquito species, Aedes (Finlaya) notoscriptus (Skuse) | JOURNAL OF VECTOR ECOLOGY | 10.1111/jvec.12194 | Vector-only study without a dengue-related outcome or context | The study focused on mosquito biology, ecology, abundance, distribution, or control without a substantive dengue-related outcome or analysis. |
| 456 | Batra | 2021 | Role of IgG against N-protein of SARS-CoV2 in COVID19 clinical outcomes | SCIENTIFIC REPORTS | 10.1038/s41598-021-83108-0 | Not dengue-specific | Neither the title nor the abstract treated dengue as a study subject or reported a dengue-related outcome or analysis. |
| 457 | Chen | 2020 | Urban mosquito management administration: Mosquito (Diptera: Culicidae) habitat surveillance and questionnaire survey in Wuhan, Central China | PLOS ONE | 10.1371/journal.pone.0232286 | Vector-only study without a dengue-related outcome or context | The study focused on mosquito biology, ecology, abundance, distribution, or control without a substantive dengue-related outcome or analysis. |
| 458 | Nouveau, L | 2021 | Immunological analysis of the murine anti-CD3-induced cytokine release syndrome model and therapeutic efficacy of anti-cytokine antibodies | EUROPEAN JOURNAL OF IMMUNOLOGY | 10.1002/eji.202149181 | Not dengue-specific | Cytokine-release-syndrome mouse model; dengue is background context only. |
| 459 | Miranda-Massari, JR | 2021 | The Effects of Vitamin C on the Multiple Pathophysiological Stages of COVID-19 | LIFE-BASEL | 10.3390/life11121341 | Not dengue-specific | COVID-19/vitamin C review; dengue is background context only. |
| 460 | Kwon, JS | 2018 | Kinetics of viral load and cytokines in severe fever with thrombocytopenia syndrome | JOURNAL OF CLINICAL VIROLOGY | 10.1016/j.jcv.2018.01.017 | Not dengue-specific | Severe fever with thrombocytopenia syndrome study; dengue is an indexing/background term only. |
|  |  |  |  |  |  |  |  |
